# Stacked or Folded? Impact of Chelate Cooperativity on the Self-Assembly Pathway to Helical Nanotubes from Dinucleobase Monomers

**DOI:** 10.1021/jacs.3c04773

**Published:** 2023-08-02

**Authors:** Marina González-Sánchez, María J. Mayoral, Violeta Vázquez-González, Markéta Paloncýová, Irene Sancho-Casado, Fátima Aparicio, Alberto de Juan, Giovanna Longhi, Patrick Norman, Mathieu Linares, David González-Rodríguez

**Affiliations:** † Nanostructured Molecular Systems and Materials Group, Organic Chemistry Department, Universidad Autónoma de Madrid, 28049 Madrid, Spain; ‡ Department of Inorganic Chemistry, Universidad Complutense de Madrid, 28040 Madrid, Spain; § Division of Theoretical Chemistry and Biology, School of Engineering Sciences in Chemistry, Biotechnology and Health, KTH Royal Institute of Technology, SE-100 44 Stockholm, Sweden; ∥ Regional Centre of Advanced Technologies and Materials, Czech Advanced Technology and Research Institute (CATRIN), Palacký University Olomouc, 779 00 Olomouc, Czech Republic; ⊥ Department of Molecular and Translational Medicine, University of Brescia, Viale Europa 11, 25123 Brescia, Italy; # Laboratory of Organic Electronics and Scientific Visualization Group, ITN, Campus Norrköping; Swedish e-Science Research Centre (SeRC), Linköping University, 58183 Linköping, Sweden; ∇ Institute for Advanced Research in Chemical Sciences (IAdChem), Universidad Autónoma de Madrid, 28049 Madrid, Spain

## Abstract

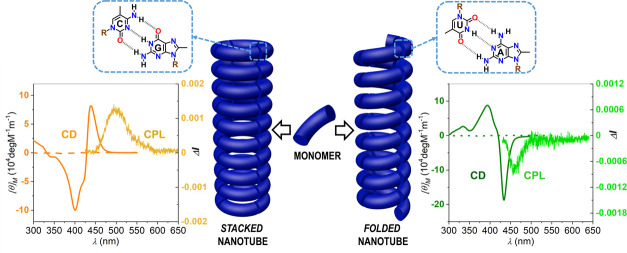

Self-assembled nanotubes exhibit impressive biological functions that have always inspired supramolecular scientists in their efforts to develop strategies to build such structures from small molecules through a bottom-up approach. One of these strategies employs molecules endowed with self-recognizing motifs at the edges, which can undergo either cyclization–stacking or folding–polymerization processes that lead to tubular architectures. Which of these self-assembly pathways is ultimately selected by these molecules is, however, often difficult to predict and even to evaluate experimentally. We show here a unique example of two structurally related molecules substituted with complementary nucleobases at the edges (*i*.*e*., G:C and A:U) for which the supramolecular pathway taken is determined by chelate cooperativity, that is, by their propensity to assemble in specific cyclic structures through Watson–Crick pairing. Because of chelate cooperativities that differ in several orders of magnitude, these molecules exhibit distinct supramolecular scenarios prior to their polymerization that generate self-assembled nanotubes with different internal monomer arrangements, either stacked or coiled, which lead at the same time to opposite helicities and chiroptical properties.

## Introduction

Tubular nanostructures are a fascinating class of nano-objects that arise an extraordinary interest in materials science and biological chemistry. The structural and electronic applications of inorganic and carbon nanotubes have revolutionized the field of nanotechnology,^1^ while the myriads of functions of biological micro- and nanotubules found in cells are essential to life.^2^ Besides their high aspect ratio, certainly the most appealing feature of tubular structures is their well-defined internal nanochannels, which can be potentially used to host and transport molecules with high selectivity, as impressively demonstrated by many transmembrane proteins.^3,4^

**Figure 1:**
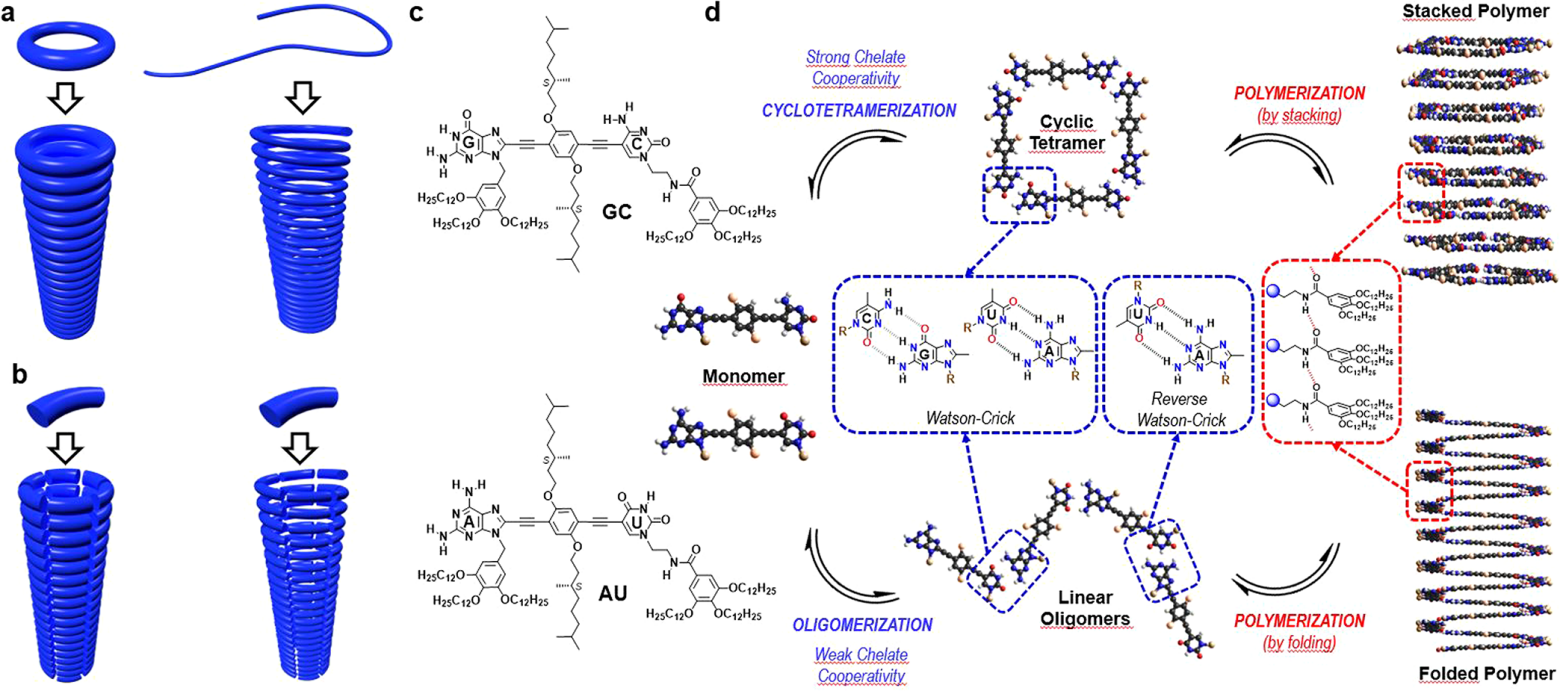
(a,b) Strategies to nanotube self-assembly through (a) the stacking of cyclic molecules or the folding of linear polymers or (b) the supramolecular polymerization of a molecule with two terminal binding sites that is able to assemble into cyclic entities or folded oligomers. (c) Chemical structure of dinucleobase monomers **GC** and **AU**. (d) Self-assembly of **GC**/**AU**. Watson–Crick pairing between complementary G:C and A:U nucleobases affords a mixture of H-bonded oligomers in equilibrium, among which an unstrained cyclic tetramer may be significantly stabilized if chelate cooperativity is strong enough. Polymerization through π–π stacking interactions and H-bonding between peripheral amides may then take place from these macrocycles (top) or from folded conformations of the linear oligomers (bottom), resulting, respectively, in stacked or coiled polymer nanotubes.

Inspired by the structure and performance of these natural systems, chemists have developed numerous approaches to nanotubes based on the self-assembly of small molecules.^5,6^ The use of amphiphilic molecules/block copolymers that aggregate in water through hydrophobic effects into cylindrical objects, instead of spherical vesicles, is certainly the simplest and most widely employed strategy when targeting relatively large tube cross sections (*i*.*e*., >10 nm).^7^ Reaching inner pore diameters that are smaller and better defined, more compatible with molecular dimensions, requires other strategies that make use of more sophisticated molecules and directional noncovalent interactions. For instance, covalent macrocycles can be made to orderly *stack* on top of each other, often aided by peripheral H-bonding units, to form cylindrical structures with well-defined pores (Figure 1a, left).^8−14^ Alternatively, relatively flexible linear oligomers can be made to *fold* intramolecularly into helical structures (*i*.*e*., foldamers; Figure 1a, right),^15^ which can also present available internal channels.

A drawback of employing these cyclic or linear oligomers for nanotube construction is that their covalent synthesis can be tedious and low-yielding. To circumvent this problem, diverse supramolecular strategies that can generate analogous architectures with lower synthetic effort have been explored. On one hand, supramolecular macrocycles^16,17^ can be assembled through diverse noncovalent interactions to define the cyclic nanotube sections (Figure 1b, left).^18^ For instance, H-bonded rosettes can be formed from molecules with suitable heterocyclic head groups,^19−24^ while cyclic structures leading to larger internal tube volumes can be accessed through diverse strategies exploiting metal–ligand,^25^ van der Waals,^26,27^ and H-bonding interactions.^28^ On the other hand, relatively short oligomers that are able to adopt helically folded conformations can also be extended *via* end-to-end noncovalent association into polymeric nanotubes (Figure 1b, right).^15,29,30^

In general, both of these supramolecular strategies rely on molecules with two noncovalent bindings sites or “sticky“ edges that undergo either intermolecular cyclization–stacking or folding–polymerization events, as shown in Figure 1b. A subtle interplay exists between these two aggregation pathways that largely depends on the structure of the monomeric molecule and on the binding interaction. Relatively flexible molecules/oligomers with some tendency to fold intramolecularly may prefer to yield helically coiled tubular structures, whereas rigid molecules with directional interactions that are able to generate unstrained cycles may opt to stack in the form of ring-shaped entities. At the same time, each of these pairs of events (*i*.*e*., cyclization+stacking and folding+polymerization) may be decoupled or coupled, meaning that the corresponding discrete supramolecular intermediate (*i*.*e*., macrocycle, folded conformer, etc.) may be detected or not prior to the completion of the aggregation process. In any case, for a monomer for which these two possible pathways are in principle available, it is difficult to predict and often to determine experimentally the kind of tubular architecture generated.

In this work, we provide an example that clearly demonstrates that the chosen pathway may largely depend on chelate cooperativity, that is, on the thermodynamic preference of a molecule to cyclize into a given ring size. Herein, we study in detail the self-assembly of two almost identical molecules (**GC** and **AU**; Figure 1c) having complementary nucleobases at their termini. We discovered that despite this structural resemblance, they assemble into supramolecular nanotubes through two rather distinct mechanisms depending on the cooperativity displayed toward the formation of a Watson–Crick H-bonded macrocycle. The molecule endowed with guanine (G) and cytosine (C) nucleobases (**GC**) enjoys high cyclization cooperativities that result in the formation of very robust cyclic tetramers (*c*(**GC**)_4_) prior to the stacking process. On the contrary, the same molecule with 2-aminoadenine (hereafter abbreviated as A) and uracil (U) complementary bases (**AU**) shows a weak tendency to cyclize into *c*(**AU**)_4_ tetramers and instead aggregates into linear polymers with folded conformations. Such distinct pathways result in tubular structures with identical diameters but opposite helicities, as experimentally demonstrated by circular dichroism (CD) and circularly polarized luminescence (CPL), and corroborated by a combination of molecular dynamics (MD) and density functional theory (DFT) calculations, and they are formed through distinct polymerization mechanisms with different nucleation-growth cooperativities.

## Results and Discussion

### **GC** and **AU**: Two Dinucleobase Monomers with a Similar Structure but Showing Important Self-Assembly Differences

Dinucleobase derivatives **GC** and **AU** (Figure 1c) share a common structure designed to yield self-assembled nanotubes based on our own previous experience.^31−34^ The complementary nucleobases were connected through a rigid and linear *p-*phenylene block attached at the purine 8- and pyrimidine 5-positions. In this way, association through Watson–Crick pairing provides a 90° angle between monomers, which can favor the formation of unstrained rectangular assemblies (*i*.*e*., *c*(**GC**)_4_ or *c*(**AU**)_4_ cyclic tetramers; Figure 1d).^35,36^ Such π-conjugated central block was at the same time substituted with *S-*chiral tails, so as to bias the helical chirality of the final tubular assemblies. On the other hand, benzylic wedges substituted with long alkyl tails were installed at the *N*-9 and *N*-1 positions of the purine and pyrimidine bases, where the deoxyribose units are located in DNA, in order to enhance π–π stacking interactions along the nanotube axis and provide solubility to the final assemblies in organic solvents. In addition, a peripheral amide group was installed at the pyrimidine nucleobase, so as to guide stacking/folding by establishing H-bonding interactions parallel to the tube’s axis (see Figure 1). In short, the only structural difference between **GC** and **AU** molecules is the exchange of carbonyl and amino groups at the *C*-6 of purines and *C*-4 of pyrimidines (see Figure 1c), but all peripheral substituents and chiral groups are exactly the same. Synthetic and characterization details can be found in our previous work (for **GC**)^31^ and in the S.I. accompanying this paper.

Both **GC** and **AU** compounds can be molecularly dissolved in their monomeric state in relatively polar solvents like THF at low concentrations. H-bonding through Watson–Crick interactions can be triggered at higher concentrations or by the addition of less polar (co)solvents, like CHCl_3_, toluene, cyclohexane, or heptane. The strength of such H-bonding interactions is greater and thus occurs at lower concentrations or in more polar solvents for the G:C pair than for the A:U pair. This is a well-known fact explained by the Jorgensen model of secondary interactions:^37^ the *DAD*:*ADA* H-bonding pattern of the A:U pair affords lower association constants (*K*) than the *DDA*:*AAD* pattern of the G:C pair (*i*.*e*., in CHCl_3_: *K*_G:C_ ∼ 2·10^4^ M^–1^; *K*_A:U_ ∼ 3·10^2^ M^–1^),^38,39^ despite both Watson–Crick pairs binding through three H-bond contacts.

**Figure 2:**
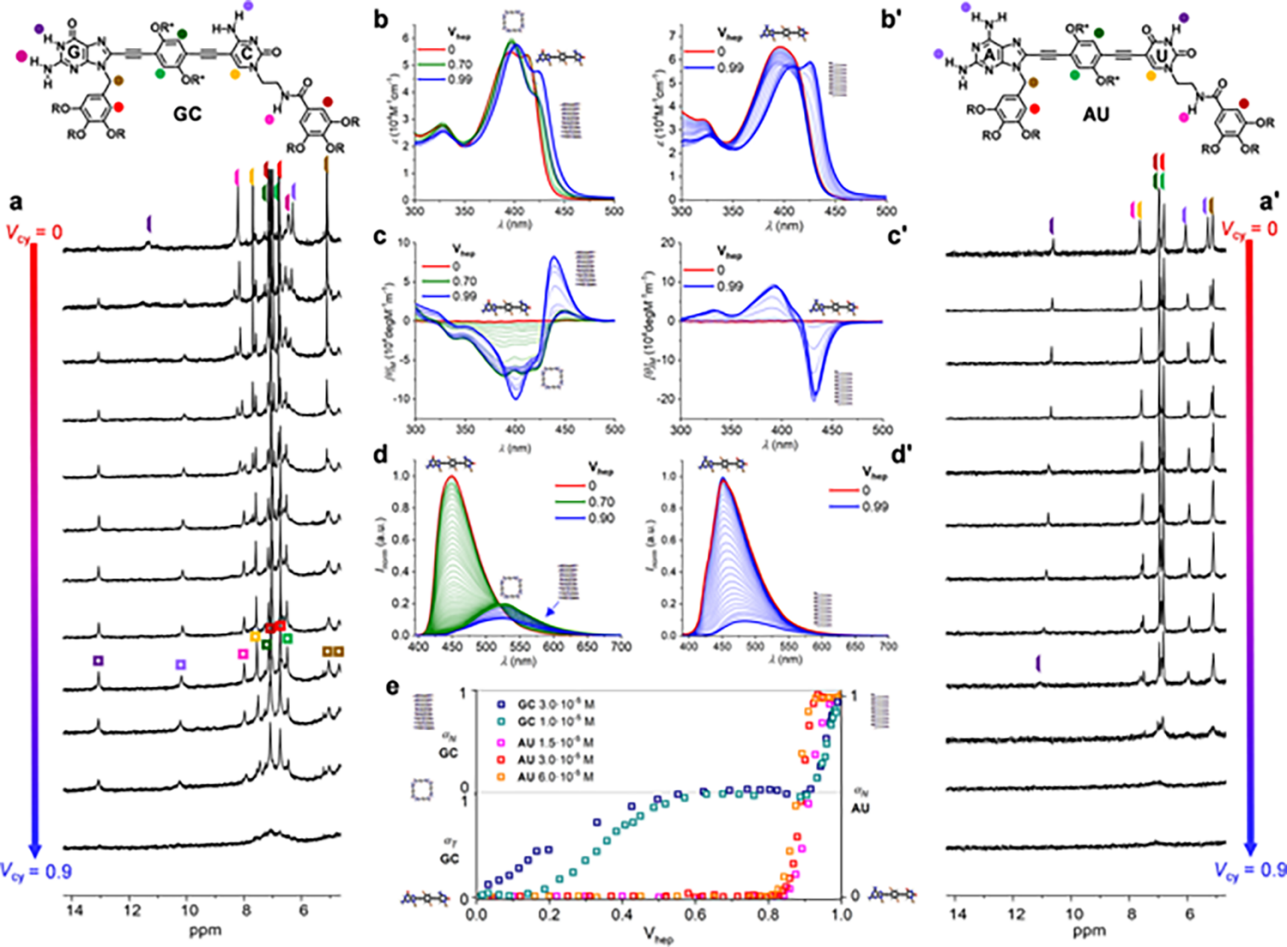
Complete self-assembly of **GC** and **AU**. Self-assembly of **GC** (**a–d**) and **AU** (**a′–d′**) by progressively increasing the volume fraction of cyclohexane-*D*_12_ (*V*_cy_; for ^1^H NMR studies) or heptane (*V*_hep_) in mixtures with THF-*D*_8_ or THF, respectively, as monitored by (**a**,**a′**) ^1^H NMR at 5.0·10^–4^ M (please, see also Figure S1B), (**b**,**b′**) absorption, (**c**,**c′**) CD, or (**d**,**d′**) emission spectroscopies at 3.0·10^–5^ M. In the ^1^H NMR signal assignments, rod-shaped marks correspond to monomers or linear oligomeric species, while square-shaped marks correspond to cyclic tetramers. (**e**) Normalized CD changes at 429 nm at several concentrations as a function of *V*_hep_ for **GC** and **AU** at 298 K (α_*T*_ = fraction of cyclotetramers, α_*N*_ = fraction of nanotubes).

Decreasing solvent polarity even further, by increasing the volume fraction of cyclohexane (*V*_cy_) or heptane (*V*_hep_) for instance, leads to higher degrees of aggregation due to an enhancement of the strength of π–π interactions between the π-conjugated segments on one hand and of H-bonding interactions between the peripheral amides purposely placed at the pyrimidine bases (C or U) on the other. Ultimately, in pure cyclohexane/heptane, we observed precipitation of the samples within the 10^–3^–10^–7^ M concentration range used in the spectroscopic measurements, which is an indication of the formation of large aggregates. Therefore, a small amount ≥1% *v/v* of a good solvent (preferably THF but also CHCl_3_) was always required to dissolve the aggregates formed by **GC** and **AU** in these apolar aliphatic environments. In this way, the whole self-assembly process could be monitored by moving from the monomer in THF to polymeric aggregates in cyclohexane-*D*_12_ (for ^1^H NMR measurements at 10^–2^–10^–4^ M) or heptane (for optical spectroscopy measurements at 10^–4^–10^–6^ M). Figure 2a–d,a′–d′, respectively, show the changes observed for **GC** and **AU** in ^1^H NMR, CD, absorption, and emission spectroscopy upon increasing *V*_cy_/*V*_hep_ in THF:cyclohexane-*D*_12_/heptane mixtures. Figure 2e displays the CD trends recorded at a fixed wavelength along the whole *V*_hep_ sweep range. Further information and the corresponding spectra and curves at other concentrations can be found in the S.I. Careful inspection of the spectroscopic data recorded along the aggregation of **GC** and **AU** allowed us to remark the following differences:

#### 1

() *In contrast to c(**GC**)_4_, c(**AU**)_4_ cyclic tetramers were not detected by any experimental technique.* Not only the strength of the intermolecular association but also the kind of Watson–Crick-bound oligomers obtained from such interactions differ for **GC** and **AU** compounds.

Monomer **GC** shows a strong tendency to cyclize into tetrameric rings, which display the typical Watson–Crick H-bonded G-amide and C-amine proton signals at *ca*. 13.5 and 10.0 ppm. As shown in Figures 2a and S1B, the ^1^H NMR spectra recorded for **GC** in THF-*D*_8_ at different *V*_cy_ values show an equilibrium between monomer **GC** and cyclic tetramer *c*(**GC**)_4_ in slow exchange at the NMR timescale, which is shifted to the macrocycle side as *V*_cy_ increases. The observation of this H-bonded species in slow NMR exchange is a solid proof for the formation of *c*(**GC**)_4_, as determined in our previous work.^31^ A similar picture is clearly observed in variable temperature experiments in THF-*D*_8_ (Figure S1A), where the whole transition from the **GC** monomer at high temperatures to the *c*(**GC**)_4_ cyclic tetramer at low temperatures can be monitored. At higher amounts of *V*_cy_, the *c*(**GC**)_4_ signals broaden and eventually vanish due to, as will be explained below, supramolecular polymerization (bottom NMR spectra in Figure 2a).

However, in the same conditions, **AU** just shows a single set of proton signals along the whole *V*_cy_ (Figures 2a′ and S1B) or temperature (Figures S1A and S1C) sweep range. The H-bonded A and U proton signals, the latter found within the 14–10 ppm window, shift downfield upon increasing *V*_cy_ or decreasing T due to their higher involvement in H-bonded species in fast NMR exchange, but a cyclic species in slow exchange is not detected in any of the experiments performed as a function of solvent, temperature, or concentration. Just like for **GC**, at very high *V*_cy_, the **AU** signals broaden and then disappear due to polymeric aggregation.

These qualitative observations are in agreement with previous studies with related G-C and A-U dinucleosides substituted with bulky lipophilic ribose groups, so as to prevent stacking interactions,^35,36^ which disclosed chelate cooperativities in their cyclotetramerization processes that can be 5 to 8 orders of magnitude higher for *c*(G-C)_4_ than for *c*(A-U)_4_. Since chelate cooperativity is quantified by the product *K·EM*, where *EM* stands for effective molarity, one of the reasons stems of course from the mentioned fact that the association constant *K* of the G:C pair is stronger than the A:U pair. However, another, even more important difference comes from the magnitude of the *EM* associated to the cyclization process. *EM* values were calculated as high as 10^2^–10^3^ M for the *c*(G-C)_4_ rings, which is an extraordinarily high value for cyclic assemblies.^40^ As a result, these kind of systems have been employed by us for manifold purposes: to study in detail chelate cooperativity,^41−43^ to self-sort fluorescent dyes,^44,45^ to produce 2D networks with well-defined nanocavities,^46^ or to efficiently disperse carbon nanotubes through a clasping mechanism.^47^ On the contrary, *EM* values as low as 10^–2^–10^–3^ M have been calculated for *c*(A-U)_4_ macrocycles.^36^ This huge difference, that spans over 4–6 orders of magnitude, was ascribed to entropic effects related to the number of degrees of freedom that are lost upon cyclization, depending on the symmetric (*DAD*:*ADA*) or unsymmetric (*DDA*:*AAD*) nature of H-bonding pattern between nucleobases (for further details, please see our previous work).^36^ For the systems studied in the current work, an *EM* value of 1.2·10^2^ M was calculated for *c*(**GC**)_4_ in THF.^32^ On the other hand, a higher *EM* limit of 10^–2^ M was estimated for *c*(**AU**)_4_, when compared to other A-U dinucleosides studied by us,^36^ but this value is probably considerably lower in view of the impossibility to detect this species even in solvents of low polarity.

#### 2

() ***GC** and **AU** displayed, respectively, 2-step and 1-step self-assembly processes*. In line with these NMR results and as noted in the spectra and curves respectively shown in Figure 2b–e, **GC** displays two transitions with clear isosbestic points in the whole *V*_hep_ range. The first one, found between *V*_hep_ ∼ 0 and 0.5 (at *ca*. 10^–4^–10^–5^ M), corresponds to the cyclotetramerization process. Then, a plateau is reached between *ca*. *V*_hep_ ∼ 0.5 and 0.9 where this highly stable *c*(**GC**)_4_ macrocycle becomes the dominant supramolecular species, and then, above *V*_hep_ ∼ 0.9, a supramolecular polymerization process is triggered. In sharp contrast, when inspecting the behavior of **AU** under the same conditions (Figure 2b′,d′,e; see also Figures S2A–B), a single transition above *V*_hep_ ∼ 0.8 attributed to a polymerization process is recorded by all techniques, and no distinct self-assembled intermediates are detected at lower polarities, which is in line with the observations made in the previous point.

A quite remarkable difference in the self-assembly of both monomers was also seen in aromatic solvents. In toluene, **GC** formed very robust *c*(**GC**)_4_ cyclic tetramers that can persist even at high temperatures at relatively low concentrations (see Figures S1C and S2G, for example).^31^ However, these macrocycles did not polymerize in this solvent even at NMR concentrations, in the mM range (see Figure S1C) and required the addition of an alkane cosolvent to induce stacking interactions. In sharp contrast, **AU** was seen to undergo the complete polymerization process in 100% toluene within the 5–80 °C range above 10^–4^ M (see Figures S1C and S2H). The isosbestic points and spectral features recorded by decreasing temperature in toluene match those seen by increasing *V*_hep_ in THF:heptane mixtures: the relevant H-bonded protons shift downfield and then they broaden and disappear (Figure S1C), absorption is red-shifted, fluorescence emission is considerably quenched, and a negative Cotton effect arises (please compare Figures S2F and S2H). Further details about the distinctive self-assembly of these molecules and their mixtures in toluene will be shown below.

#### 3

**Figure 3:**
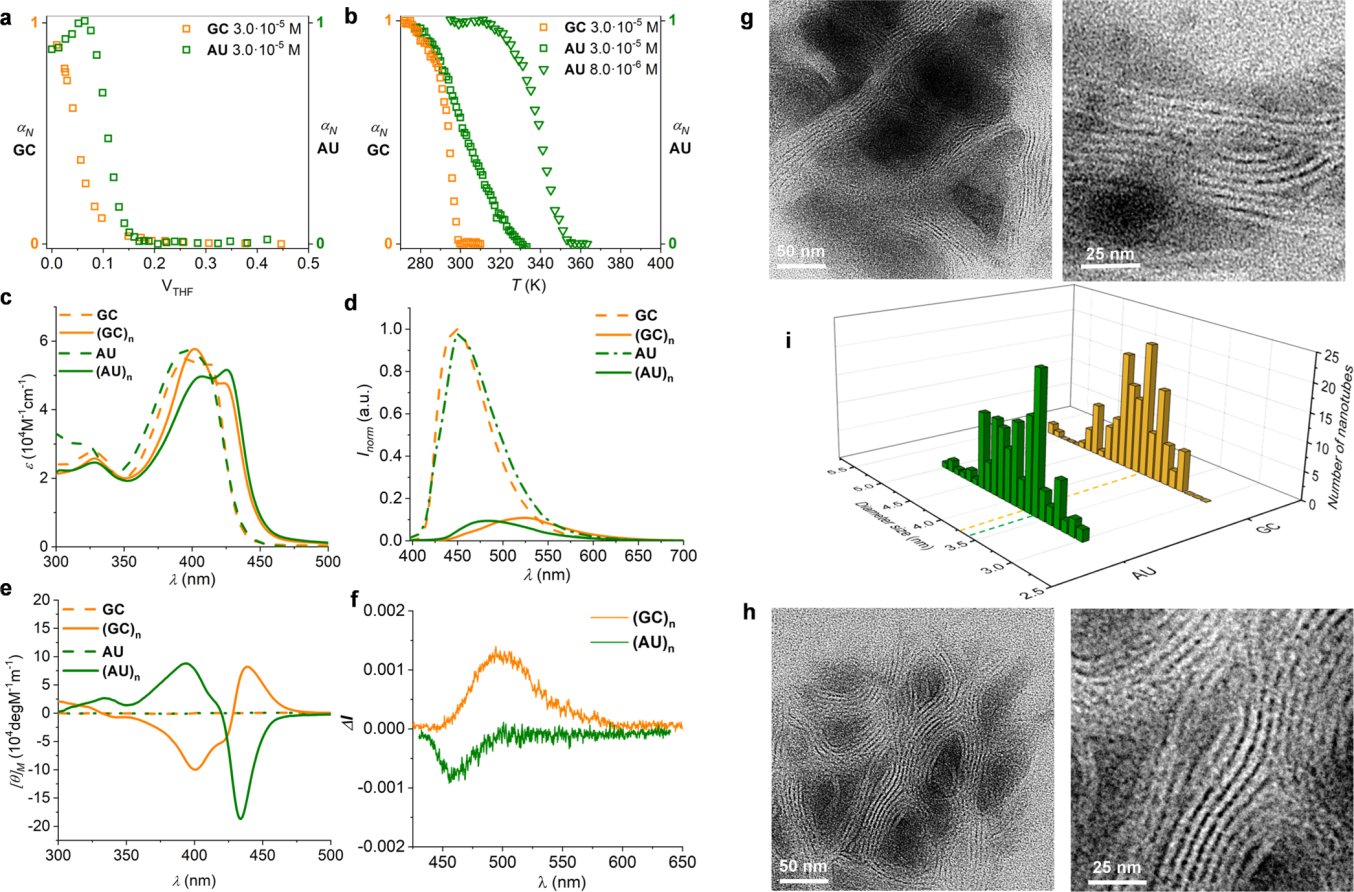
Spectroscopic and morphological differences in the self-assembly of **GC** and **AU**. (a, b) CD trends recorded at 429 nm (**GC**) or 430 nm (**AU**) as a function of (**a**) the volume fraction of THF (*V*_THF_ = 1 – *V*_hep_) in THF:heptane mixtures at 298 K or (**b**) the temperature in THF:heptane mixtures at *V*_hep_ = 0.97 ([**GC**] = 3.0·10^–5^ M (orange squares); [**AU**] = 8.0·10^–6^ M (green triangles)) or *V*_hep_ = 0.90 ([**AU**] = 3.0·10^–5^ M (green squares)). (c) Absorption, (d) emission, (e) CD, and (f) CPL spectra of the (**GC**)_n_ and (**AU**)_n_ polymers (solid lines) at *V*_hep_ = 0.99 compared to the **GC** and **AU** monomers (dashed lines) at *V*_hep_ = 0. (g, h) TEM images of the assemblies formed by (g) **GC** and (h) **AU** drop-cast from diluted solutions of high *V*_hep_. (i) Nanotube diameter distributions measured by TEM.

() ***AU** exhibited a much stronger aggregation tendency but lower nucleation-growth cooperativities than **GC.*** The supramolecular polymerization/depolymerization transitions of **GC** and **AU** were recorded in both solvent- and temperature-dependent experiments and analyzed by their corresponding mathematical models (see S.I. Section S2 for details). The degree of cooperativity (σ), defined as the ratio between the nucleation and elongation equilibrium constants (σ = *K*_n_/*K*_e_), was calculated and compared for these two molecules. From the denaturation curves as a function of solvent composition at different overall concentrations, obtained by increasing the volume fraction of THF (*V*_THF_) in mixtures with heptane, moderate and comparable σ values in the order of 10^–1^ (Figures 3a and S2B and Table S1) were obtained for both molecules. An additional observation obtained from these experiments is that the polymerization of **AU** occurs at considerably lower heptane contents than the polymerization of **GC**, which is sequestered as *c*(**GC**)_4_ cycles, at the same concentration (see Figures 2e or 3a).

Experiments carried out at a fixed solvent composition (Figures 3b and S2D–I), in which temperature is varied at low rates with <0.1 K accuracy and a global fitting can be made from measurements at different concentrations, are generally preferred for a precise thermodynamic control of the nucleation and elongation events and to supply more accurate and reliable thermodynamic parameters.^48−52^ Once again and as shown in Figure 3b, the propensity of **AU** to aggregate in polymeric tubes was observed to be much higher in the same solvent conditions than that of **GC** (or, more exactly, *c*(**GC**)_4_). For instance, at the same concentration (3.0·10^–5^ M) **AU** and **GC** required 10:90 and 3:97 THF–heptane solvent mixtures, respectively, to record the whole polymerization process within our experimental temperature window. Likewise, in the same 3:97 THF–heptane solvent mixture, the elongation temperature (*T*_e_) determined for **AU** was more than 50 K higher than the one measured for **GC**, even if the concentration of the former compound had to be decreased from 3.0·10^–5^ M to 8.0·10^–6^ M to detect the nucleation event (see Figures 3b and S2D,E). Unfortunately, the cyclotetramerization and the polymerization events of **GC** occur within very different experimental windows and, due to technical limitations, we could not monitor both processes consecutively as a function of the temperature in a single solvent system (like we did in the solvent-dependent experiments).

Moreover, in these temperature-dependent experiments, we observed some marked differences in the cooperative polymerization of both molecules, as graphically revealed in Figure 3b. The polymerizations of **GC** enjoyed higher cooperativities (σ = 3.0·10^–4^ at *V*_hep_ = 0.97) than those of **AU** (σ = 1.0·10^–2^ at *V*_hep_ = 0.97, σ = 2.6·10^–1^ at *V*_hep_ = 0.90 and σ = 1.1·10^–1^ in toluene), as shown in Figure S2I and Table S2. Both compounds displayed at *V*_hep_ = 0.97 with similar equilibrium constants for the elongation phase (*K*_e GC_ = 1.3·10^5^; *K*_e AU_ = 1.8·10^5^), and the differences in cooperativity stem from a smaller equilibrium constant of the nucleation stage (*K*_n GC_ = 4.0·10^1^; *K*_n AU_ = 1.8·10^3^).

In all of these solvent- or temperature-dependent experiments, we made sure that we worked under equilibrium conditions and that the observed deviations are not caused by time or solvent effects. The final spectroscopic features did not evolve with time or thermal annealing, and cooling and heating curves converting the monomer into the aggregate and *vice versa* perfectly overlapped at various concentrations and solvents (see Figures S2F and S2H).

#### 4

() *Despite their virtually identical structure, **GC** and **AU** molecules displayed quite different aggregate spectroscopic features.* A final, but most remarkable difference between the supramolecular polymers of **GC** and **AU** comes from the analysis of their basic spectroscopic characteristics. As already noted, the final absorption, emission, and CD spectra of (**GC**)*_n_* and (**AU**)*_n_* polymeric aggregates is considerably different. For the sake of comparison, we display the absorption, emission, CD, and CPL spectra for both compounds in their monomeric (*V*_hep_ = 0) and polymeric (*V*_hep_ = 0.99) form in Figure 3c–f (see also Figure S2C). First of all, both molecules develop a red-shifted absorption band upon polymerization (Figure 3c), but the one disclosed by **AU** is significantly more pronounced and shifted (Δλ = 12 nm) than the one of **GC** (Δλ = 6 nm). On the other hand, both molecules experience a significant decrease in emission intensity (83% quenching for **GC** and 87% for **AU**; Figure 3d) and a notable red shift in emission maxima when comparing polymer and monomer samples, but this shift is now smaller for **AU** (Δλ = 36 nm) than for **GC** (Δλ = 75 nm). It is important to remark, as can be appreciated in Figure 2b–d, that the cyclotetramerization process experienced by **GC**, just like all G-C dinucleosides studied by the group (please, see our previous work),^40^ already brings about important spectroscopic changes that are attributed to the loss of degrees of freedom and planarization of the π-conjugated phenylene–ethynylene system. For instance, fluorescence emission is quenched and red-shifted upon cyclization (red to green lines in Figure 2d), whereas stacking of these cyclotetramers into polymeric tubes results in a further reduction of fluorescence emission and a slight blue shift (green to blue lines in Figure 2d).

Nevertheless, the spectroscopic differences between (**GC**)*_n_* and (**AU**)*_n_* polymers are even more pronounced in the CD and CPL spectra in THF:heptane solutions at *V*_hep_ = 0.99. As shown in the CD spectra in Figure 3e, (**AU**)*_n_* polymers display a negative Cotton effect with maxima at 333(+), 394(+), and 433(−) nm, whereas (**GC**)*_n_* polymers show a positive Cotton effect with maxima at 341(−), 402(−), and 440(+) nm. On the other hand, Figures 3f and S2K,L show the CPL spectra of (**GC**)*_n_* and (**AU**)*_n_*. In both cases, the CPL band is in correspondence with the high energy side of the fluorescent feature, and this is particularly evident for (**AU**)*_n_*, which presents a blue shift of about 25 nm. The CPL sign is the same as the sign for the lowest-energy CD band, as expected and observed in most cases, so the information about the opposite chiral helicities can be obtained both through CD and/or CPL. Indeed, comparing (**GC**)*_n_* and (**AU**)*_n_*, they display almost, but not exactly (due to an evident wavelength shift), mirror image CD and CPL features, despite bearing the same *S-*chiral center (Figure 3e,f). In line with all other spectroscopic techniques, the CPL spectrum recorded for (**AU**)*_n_* in toluene (Figure S2L) is similar, although slightly more intense, to the one found in THF:heptane. Furthermore, one may notice that, in toluene, CPL and fluorescence bands are centered at the same wavelength.

In short, we would like to emphasize that the chiroptical response of (**GC**)*_n_* and (**AU**)*_n_* nanotubes are *almost* a mirror image but not exactly due to the differences noted in absorption and emission maxima. This suggests that the differences in the internal organization of each dinucleobase molecule in the polymeric aggregates must also go beyond a simple mirror image relationship.

On the other hand, the CPL spectra observed for the two aggregation states of **GC** (*c*(**GC**)_4_ and (**GC**)*_n_*), measured, respectively, at *V*_hep_ = 0.40 and *V*_hep_ = 0.99, are quite similar in shape and wavelength (see Figure S2K). The only difference comes from a decrease of the CPL signal from *c*(**GC**)_4_ to (**GC**)*_n_*, which is different from the changes observed in the CD spectra (see Figure 2c). However, we should consider that CPL originates from the lowest energy transition, while several transitions contribute to CD and may partially cancel each other.

#### 5

() *Both **GC** and **AU** form self-assembled nanotubes with similar diameters that coincide with a tetrameric cross section.* Dried samples drop-cast from solvent mixtures at high *V*_hep_ were analyzed by means of scanning and transmission electron microscopy (SEM and TEM) experiments, so as to compare the morphology and dimensions of the final aggregates formed by **GC** and **AU** molecules. A general conclusion from all microscopy experiments performed is that the nanotubes formed by both dinucleobase compounds displayed a large tendency to bundle in solution and that the longer the time before deposition, the higher the degree of nanotube bundling. As a matter of fact, when **GC** or **AU** was left for several days in solutions of high *V*_hep_, a precipitate emerged, especially in concentrated samples. Bundling was beneficial for a successful detection of the nanotubes onto the grid but detrimental for the study of isolated nanotubes. The best electron microscopy images were obtained when the solution was prepared 2–3 days before deposition. SEM measurements of Cu-metallized samples deposited onto glass substrates (Figures S3A–B), as well as low-voltage TEM measurements carried out on stained samples (Figures S3C–D), revealed the formation of dense networks of large longitudinal entangled aggregates, but the diameter of the individual constituents could not be determined. Working at higher voltages with nonstained samples deposited onto copper grids coated with carbon, conditions that provided higher contrast and resolution, allowed us in contrast to image individual (**GC**)*_n_* and (**AU**)*_n_* nanotubes, as shown in Figures 3g,h and S3C–D.

The analysis of multiple TEM images (Figure 3i) afforded a mean nanotube diameter of 3.9 ± 0.7 nm for (**GC**)*_n_*, dimensions that are in agreement with previous SAXS and DLS measurements^31^ and that match the aromatic hard section of the *c*(**GC**)_4_ cyclic tetramers. For the (**AU**)*_n_* nanotubes, a slightly narrower mean diameter of 3.8 ± 0.4 nm was calculated. Aside from these minor differences, the dimensions of the nanotubes formed by **GC** and **AU** dinucleobase molecules were virtually indistinguishable, and their internal stacked/folded helical structure could not be elucidated by means of any microscopy technique we utilized in this work. This included the use of aberration-corrected high-resolution TEM techniques, which unfortunately resulted in rapid nanotube decomposition under the high-power electron beam.

### **GC** and **AU** Narcissistically Self-Sort along Their Self-Assembly Processes

Points 1–3 above support the notion that the overall aggregation mechanism diverges for *S*-chiral dinucleobase molecules **GC** and **AU**, despite their almost identical structure. The first difference comes at the early stages of the aggregation process, where **GC** fully associates in cyclic species, which are nonetheless not detected for **AU**. However, one might argue that most examples of nanotube aggregation from molecules forming, for instance, H-bonded rosette-type assemblies also occur without the detection of cyclic intermediates,^19−24^ so this could just be the same case. This means that, as soon as small (nondetected) amounts of *c*(**AU**)_4_ cyclic species are formed in solution, they may act as (pre)nuclei for the coexisting monomer and short H-bonded oligomers to trigger polymerization. In other words, cyclotetramerization and polymerization would be decoupled for **GC** due to the extraordinary stability of the *c*(**GC**)_4_ macrocycles but could be strongly *coupled* for **AU**. However, if this was the case, we would expect that both final (**AU**)*_n_* and (**GC**)*_n_* polymer products, both composed of comparable stacked macrocycles, should show similar spectroscopic properties. Although the images recorded by TEM, as disclosed in point 5 above, reveal nanostructures of matching morphology and diameter for (**AU**)*_n_* and (**GC**)*_n_*, the spectroscopic observations exposed in point 4 clearly show that the internal structure of each final nanotube is markedly different. Moreover, as noted in point 3, **AU** reveals a notably stronger propensity to polymerize than **GC**, although with poorer cooperativity, which also disagrees with the idea that tiny amounts of *c*(**AU**)_4_ could act as nucleation seeds for polymerization.

**Figure 4:**
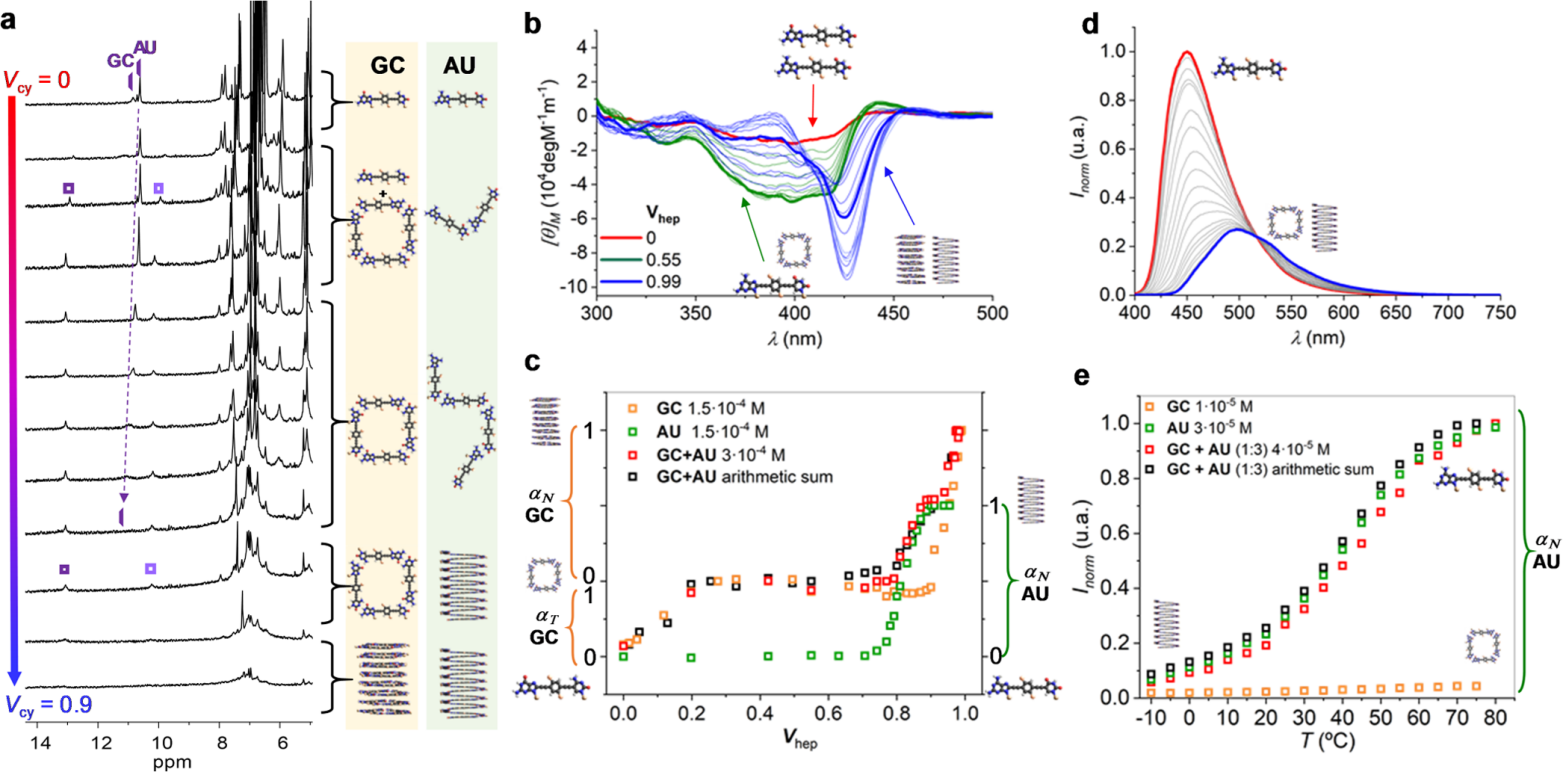
Self-sorting experiments. (a) Self-assembly of a 1:1 mixture of **GC** + **AU** by progressively increasing the volume fraction of cyclohexane-*D*_12_ (*V*_cy_) in mixtures with THF-*D*_8_ monitored by ^1^H NMR ([**GC**] = [**AU**] = 2.0·10^–3^ M; *T* = 298 K; see also Figures S4A). The pictures at the right indicate approximately the distribution of supramolecular **GC** and **AU** species as *V*_cy_ is increased. Please compare with Figure 2, where the individual evolution of **GC** and **AU** is displayed. (b) Self-assembly of a 1:1 mixture of **GC** + **AU** by progressively increasing the volume fraction of heptane (*V*_hep_) in mixtures with THF monitored by CD ([**GC**] = [**AU**] = 1.5·10^–4^ M; *T* = 298 K; see also Figures S4B). (c) Normalized CD changes at 435 nm as a function of *V*_hep_ for **GC**, **AU**, their mixture (spectra shown in panel (b)), and the arithmetic sum of **GC**+**AU** CD intensity taken from the isolated samples (α_*T*_ = fraction of cyclotetramers, α_*N*_ = fraction of nanotubes). (d) Self-assembly of a 1:3 mixture of **GC** + **AU** monitored by emission spectroscopy by progressively decreasing temperature in a THF:heptane mixture at *V*_hep_ = 0.9 ([**GC**] = 1.0·10^–5^ M; [**AU**] = 3.0·10^–5^ M; see also Figures S4C). (e) Normalized emission changes at 450 nm as a function of *T* for **GC**, **AU**, their mixture (spectra shown panel (d)), and the arithmetic sum of **GC**+**AU** emission intensity taken from the isolated samples.

In order to fully discard the fact that the polymerization of **AU** does not proceed *via* stacking of *c*(**AU**)_4_ macrocycles, we carried out self-sorting experiments^53−57^ (Figure 4) by mixing both **GC** and **AU** molecules. We know from recent studies that a strong narcissistic self-sorting process operates along the cyclotetramerization of these kinds of dinucleobase monomers, which is mainly driven by a strong chelate cooperativity, in addition to the different complementary H-bonding patterns of the G:C and A:U pairs.^45^ However, the supramolecular picture during polymerization may be very different. We hypothesized that if **AU** polymerized in the form of *c*(**AU**)_4_ cycles and/or required nuclei formed by such cyclic entities, then *c*(**AU**)_4_ and *c*(**GC**)_4_ should costack along polymerization, thus leading to statistically mixed assemblies. In other words, we would expect the absence of self-sorting effects during the polymerization of *c*(**AU**)_4_ and *c*(**GC**)_4_ macrocycles due to their strong structural resemblance and to the fact that they are endowed with exactly the same peripheral groups and chiral tails.

Thus, from all of the data gathered so far, we planned a set of experiments that could discern if the aggregation pathways of **GC** and **AU** are independent or if, on the other hand, these molecules mix in the polymeric assemblies. This is not trivial, in view of the identical absorption and emission windows of these two chromophores and the related experimental conditions under which they polymerize. However, by playing with relative monomer concentration and solvent composition, we were able to find conditions in which we could (1) scan the whole aggregation landscape and record consecutively **GC** cyclotetramerization, **AU** polymerization, and *c*(**GC**)_4_ polymerization and (2) monitor **AU** polymerization in the presence of *c*(**GC**)_4_ macrocycles.

The first case was realized by increasing *V*_cy_/*V*_hep_ in THF:cyclohexane-D_12_/heptane mixtures, and could be respectively monitored by ^1^H NMR and by optical spectroscopy under experimental conditions that are similar to those shown in Figure 2. In the ^1^H NMR experiments, as shown in Figures 4a and S4A, our starting situation at *V*_cy_ = 0 and 2.0·10^–3^ M concentration in each compound (top spectrum) reveals a mixture of dissociated **AU** and **GC** monomers. By gradually increasing *V*_cy_, we basically observe the same changes shown independently by each compound (Figure 2): the H-bonding protons of **AU** experience a downfield shift that indicates the formation of a fast exchanging mixture of H-bonded oligomers, whereas the *c*(**GC**)_4_ proton signals appear and increase in intensity at the expense of the **GC** monomer signals. Further decrease in solvent polarity within the 0.3 < *V*_cy_ < 0.7 range kept downshifting the H-bonded proton signals of **AU**, indicating the formation of larger oligomers, while the *c*(**GC**)_4_ signals remained unchanged. Above *V*_cy_ > 0.75, the **AU** proton signals begin to broaden significantly and then disappear, indicating the formation of polymer nanotubes. In the 0.75 < *V*_cy_ < 0.85 solvent composition window, the (**AU**)*_n_* polymers and *c*(**GC**)_4_ macrocycles coexist, but the supramolecular behavior of each species does not seem to influence the other. Finally, above *V*_cy_ > 0.85, the *c*(**GC**)_4_ signals start to broaden and then disappear, revealing the polymerization of this species as well.

While these NMR experiments are very clear and highly informative, a quantitative comparison between the supramolecular behavior of *S-*chiral **AU** and **GC** molecules when isolated and in mixtures could be more accurately obtained from optical spectroscopy experiments and in particular from CD and emission spectroscopy due to the distinct signatures of *c*(**GC**)_4_, (**GC**)*_n_*, and (**AU**)*_n_* (see Figures 2 and 3). A screening of multiple concentrations and [**GC**]/[**AU**] ratios allowed us to choose [**GC**] = [**AU**] = 1.5·10^–4^ M as the best conditions to record consecutively all self-assembly processes by increasing *V*_hep_ in THF/heptane mixtures. As revealed in Figures 4b,c and S4B, the cyclotetramerization process of **GC** is first monitored within the *V*_hep_ = 0–0.3 range. Then, the polymerization of each compound is monitored successively at higher *V*_hep_ values: **AU** polymerizes first at *V*_hep_ > 0.8, while (**GC**)*_n_* polymerization is activated just after *V*_hep_ > 0.9. Interestingly, the trends observed for the supramolecular processes of *S-***AU** and *S-***GC** in these mixtures match quite reasonably those seen by the same molecules independently (see Figures 4c and S4B), suggesting again a narcissistic self-sorting behavior.

Inferring from the presented spectroscopic data, one might argue that the reason behind the opposite chirality of (**AU**)*_n_* with respect to (**GC**)*_n_* might reside in that the fragile *c*(**AU**)_4_ species also presents opposite chiral with regards to *c*(**GC**)_4_. In this scenario, the clear independent aggregation pathways observed in the **GC**+**AU** mixtures could be originated from *chiral self-sorting*, which has been demonstrated to play a crucial role in self-assembly.^58−62^ We believe this is an improbable situation since **GC** and **AU** share the same *S-*chiral groups and because the most strikingly different feature in the polymerization of **AU** is not the opposite CD sign generated but probably its much stronger predisposition to polymerize, compared to **GC**, even if the *c*(**AU**)_4_ species is far less stabilized. In any case, in order to discard chiral self-sorting events, the same solvent-dependent experiments shown in Figure 4c,d by mixing *S-***GC** and *S-***AU** were now performed by mixing *R-***GC**(31) and *S-***AU** molecules under the same conditions. The results, shown in Figure S4C, reveal the same strong narcissistic self-sorting process with almost identical transitions for the heterochiral and the homochiral mixtures, thus discarding the existence of chiral self-sorting mechanisms that would generate (**AU**)*_n_* from oppositely chiral *c*(**AU**)_4_ macrocycles.

Lastly, the robustness of the *c*(**GC**)_4_ macrocycle enabled us to explore several conditions in which **AU** polymerization can be recorded as a function of the temperature in the presence of this cyclic species. This can be done, for instance, using THF:heptane mixtures at *V*_hep_ = 0.9 and [**GC**] = 1.0·10^–5^ M and [**AU**] = 3.0·10^–5^ M concentrations. As displayed in Figures 4d,e and S4D using fluorescence emission, a temperature variation between 373 and 263 K does not affect significantly the integrity of the *c*(**GC**)_4_ cycle in this solvent environment, while the whole **AU** polymerization process is recorded. The same conclusion was derived by monitoring the aggregation processes of **AU** + **GC** mixtures by CD spectroscopy in toluene (Figure S4E), a solvent in which, as noted above, **AU** polymerizes but not **GC**, which remains sequestered as *c*(**GC**)_4_ cycles even at low temperatures and/or high concentrations. Once again, the fact that **AU** polymerization follows the same transitions without or with comparable amounts of the **GC** molecule confirms that these molecules self-sort narcissistically forming their own aggregates.

### Simulations of Supramolecular Structures and Spectra

To reveal and analyze the underlying microscopic reasons for the different spectroscopic features of the supramolecular assemblies of **GC** and **AU**, we performed molecular dynamics (MD) simulations of the respective nanotube formations. We adopted a bottom-up approach and started by performing a study of the structure and conformational preferences of the monomers in a bulk solvent (99% heptane and 1% THF), as shown in Figures S5A,5B and Table S3. Next, we studied the adopted assemblies in small stacked cyclic systems. We prepared models of *c*(**GC**)_4_ and *c*(**AU**)_4_ by arranging four monomers into squares and placing them on top of each other in two layers (2SQ) or eight layers (8SQ). The 2SQ simulations showed that **GC** preferred to stay in the form of two separate squares (Figure S5C). In the case of **AU**, on the other hand, the squares did not stay intact but instead broke and finally all eight monomers were interconnected in helix-like structures. A similar observation was valid also for the 8SQ systems (Figure S5D and Table S4): we observed stable squares in the case of **GC** but the disruption of several squares and the formation of a helix in the case of **AU**.

**Figure 5:**
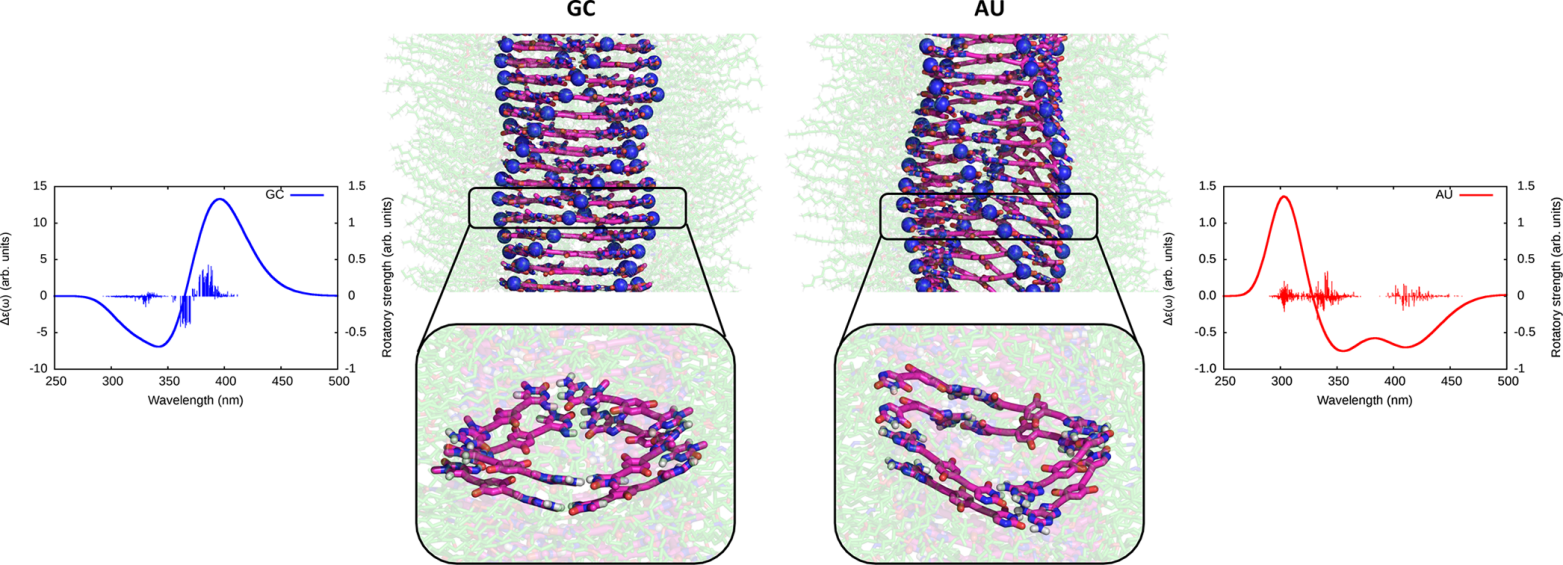
Simulations of supramolecular structures and spectra. Final structures of **GC** and **AU** nanotube models with molecular cores shown in magenta, A and G nitrogens in blue, and side chains as semitransparent green sticks. The nanotube models have opposite twist. The insets show the details of the nanotube core conformation. At each side, the CD spectra calculated as ensemble average over 10 snapshots extracted from the MD simulations is shown. The thin vertical lines show rotatory strengths from individual excited states. Units are arbitrary but comparable in between the two systems.

Based on the 8SQ systems, we built larger assemblies, resulting in simulations of infinite nanotubes with periodic boundary conditions (for details, see SI Section S5.2.3). Building and simulating periodic **GC** and **AU** nanotubes resulted in stable systems with different twists and geometries. Short segments of these helical structures are illustrated in Figure 5, where selected nitrogen atoms in the nucleobases are depicted as spheres to better appreciate the helicity. Macroscopically, both **GC** and **AU** formed nanotubes with a diameter of about 3–4 nm, considering the rigid core, and about 7–8 nm, including the peripheral chains (Figure S5I), which agrees with the TEM analyses. Microscopically, however, we observed some differences between these two systems. The squared intact **GC** system was more compact, showing an interlayer π-stacking distance of 0.36 nm (Figure S5H). In contrast, the layers in the **AU** system were interconnected in a spiraling structure and separated by a π-stacking distance of 0.40 nm (Figure S5F). Both systems were able to establish stabilizing H-bonds between peripheral amides, although the average number of these amide–amide bonds was slightly higher for the **AU** nanotube (Table S5). However, the overall number of H-bonds in the **GC** nanotube is higher (15.6 per four molecules) than that in **AU** (14.3, Table S5). This difference does not come from base pairing or peripheral amide–amide interactions but mostly from the contribution of H-bonds between side chain oxygens and the exocyclic amine groups of purines, which are more abundant for guanine (Figure S5J). Also, the mutual orientation of **GC** molecules was mostly square-like (∼90° between the residues), whereas **AU** edges were not so regular, corresponding to an overall helix organization (Figure S5L). We believe that the different orientations originate from the different preferential monomer conformations (Figure S5B). We also observed that while the **GC** molecules preferred a planar conformation, the **AU** molecules were mostly rotated and therefore preferred a helical reorganization (Figures 5 and S5L).

Finally, we put the supramolecular model structures to test by calculating the associated CD spectra and compare them against the experimental results. In our experience,^63−68^ CD spectra are very sensitive to the (supra)molecular structure and even a successful qualitative comparison between theoretical and experimental spectra can provide strong evidence for the structure model to be sound. Supramolecular systems in general, and our nanotubes are no exception to this rule, are sufficiently dynamic at room temperature to mandate a configuration space sampling when performing CD spectrum simulations. Therefore, we extracted 10 snapshots from the MD simulations of the periodic **GC** and **AU** systems and calculated the associated ensemble averaged CD spectra with use of an exciton coupling model that took four nanotube layers into account. The decision to use four layers in the calculation was made after performing a large-scale benchmark calculation for a 10-layer system (Figure S5M). The calculated CD spectra confirmed the relevance of the presented model, as their shapes were in full agreement with experimentally observed CD spectra (compare Figures 3 and 5). In the case of the **AU** system, the procedure of ensemble averaging turned out to be of vital importance as several of the individual spectra showed to qualitatively differ from the averaged one (Figure S5O). In the case of the **GC** system, on the other hand, the individual spectra were in close agreement, which, in turn, resulted in an averaged CD spectrum of higher intensity compared to **AU** (Figure S5N). The trace origin of the CD signal turned out not to be the π-stacked nucleobases but rather the central phenylene cores bearing the chiral tails. This could be established after performing spectrum simulations for a system where the nucleobases had been removed and which showed a preserved band shape (Figures S5P and S5Q). As far as the CD responses are concerned, the role of the nucleobases is thus to dictate the formation of the supramolecular structure of the nanotubes and thereby also the 3D orientation of the central cores, which in turn give rise to the characteristic bands by means of induced CD though the effect of the exciton coupling mechanism.

In short, theoretical simulations, combining MD and DFT calculations, are able to explain the differences in the CD spectrum of (**AU**)*_n_* and (**GC**)*_n_* nanotubes from structural preferences of these dinucleobase molecules upon self-assembly at the nanoscale: while **GC** tends to associate in planar squares that stack in a right-handed helical organization, **AU** forms oligomers that fold into a spiral with a left-handed helical twist.

### Impact of Chelate Cooperativity on the Aggregation Process

**Figure 6:**
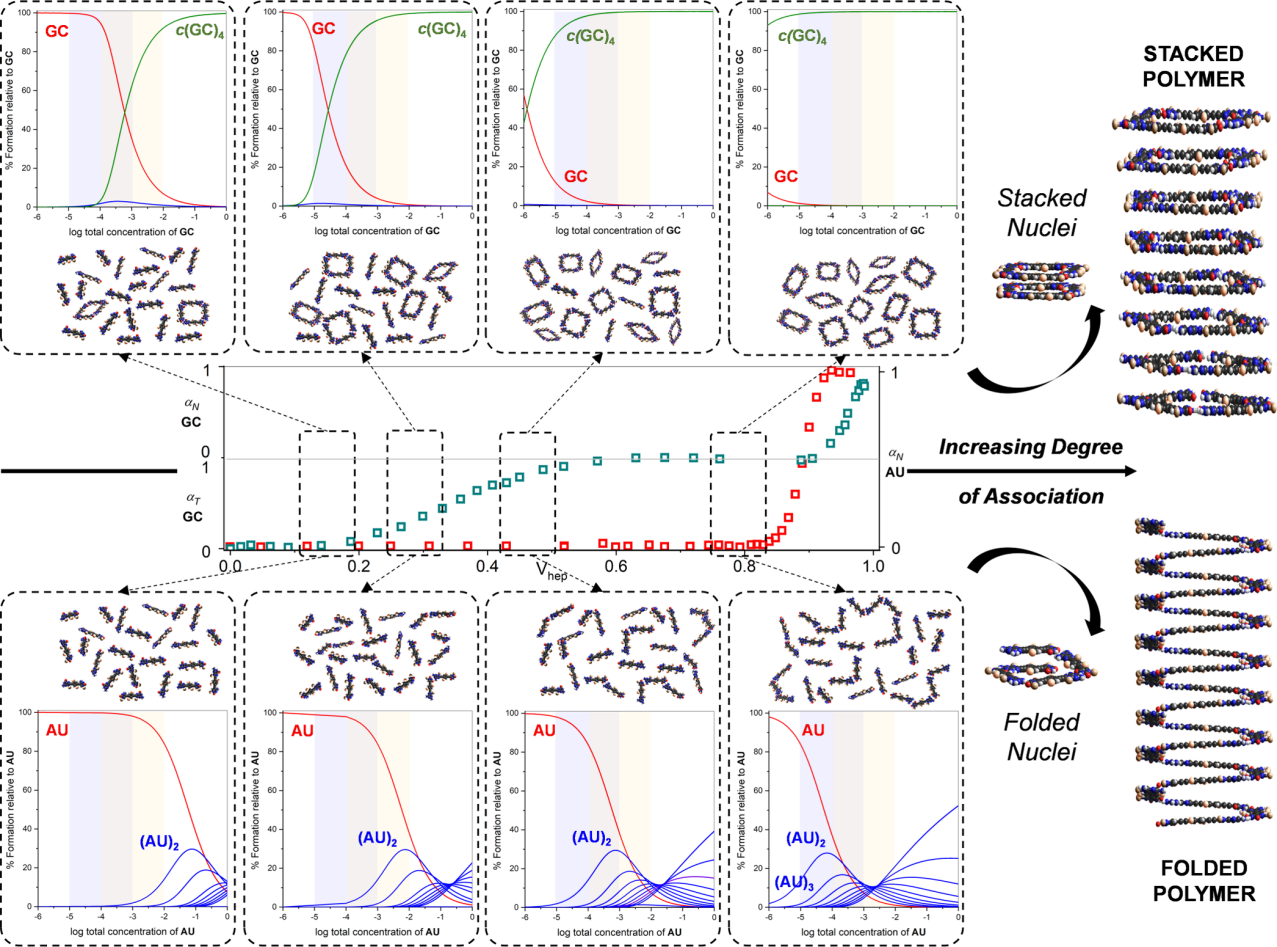
Impact of chelate cooperativity on the self-assembly pathway and nanotube structure. Schematic representation of the whole supramolecular self-assembly process leading to nanotubes with the proposed stacked or folded internal structures. We simulate here the supramolecular scenarios encountered by **GC** (top panel) or **AU** (bottom panel) as *V*_hep_ is increased in THF:heptane mixtures and hence the intermolecular association strength, when going from left to right. In the middle, a simplified version of the panel shown in Figure 2e is reproduced, which shows the experimental evolution of the **GC** cyclotetramerization, **AU** polymerization, and **GC** polymerization with increasing *V*_hep_. Each of the dashed frameworks at the top and the bottom provides a “snapshot” of the distribution of supramolecular species present in solution at 4 selected *V*_hep_ ranges before polymerization is triggered. Each of these frameworks contains the corresponding speciation curves in which the distribution of Watson–Crick H-bonded oligomers, which includes open oligomers from the dimer to the decamer (in blue), the cyclic tetramer (in green), and the monomer (in red), is simulated as a function of the total concentration. Orange and purple bands indicate, respectively, the concentration range employed in the ^1^H NMR and optical spectroscopy experiments performed in this work. Chelate cooperativity is several orders of magnitude higher for *c*(**GC**)_4_ than for *c*(**AU**)_4_. As a result, **GC** undergoes an “all-or-nothing” association process in which the cyclic tetramer is in equilibrium with the monomer, while **AU** mostly self-associates in a mixture of open (non-cyclic) species. As *V*_hep_ increases from left to right, the population of Watson–Crick H-bonded species increases until polymerization can be triggered at very high heptane contents (*V*_hep_ > 0.8). At this point, the *c*(**GC**)_4_ macro*c*ycles are formed quantitatively in solution, whereas **AU** oligomers are long enough to become stabilized through folding interactions. Polymers originating from these two different situations can have a tubular structure with stacked or coiled molecular arrangements.

In view of all experimental data exposed so far, our own experience with dinucleoside macrocycles, and the revealing picture derived from computational studies, we propose a scenario in which (**GC***)_n_* and (**AU**)*_n_* tubular polymers grow with very different mechanisms that ultimately lead to similar nanomorphologies, though different internal structures. In order to understand the pathway that each of these molecules take prior to polymerization, we are showing in Figure 6 diverse “supramolecular scenes” along the aggregation process of **GC** (top) and **AU** (bottom) that would be obtained as we enhance, from left to right, the degree of association by, for instance, increasing the volume fraction of apolar solvent (*V*_hep_) or decreasing temperature (*T*). Higher *V*_hep_ or lower *T* values lead in general to stronger interactions between molecules and, in particular, before polymerization takes place, to larger Watson–Crick H-bonding association constants for both G:C and A:U pairs. In order to simulate the “scenes” before polymerization, we built speciation curves that show the distribution of H-bonded species (from the **GC**/**AU** monomer (in red) to linear oligomers up to the decamer (**GC**)_10_/(**AU**)_10_ (in blue), including *c*(**GC**)_4_/*c*(**AU**)_4_ cyclic tetramers (in green)) as a function of concentration. The two variables employed to build these profiles are the association constant between nucleobases (*K*_G:C_ and *K*_A:U_) and the effective molarities for cyclotetramerization (*EM*_GC_ and *EM*_AU_). We started with the association constants calculated in THF at 298 K (*K*_G:C_ = 4.1·10^2^ M^–1^^32^ and *K*_A:U_ = 1.7·10^1^ M^–1^), and then, these values were progressively increased from left to right, thus simulating the effect of having higher *V*_hep_ or a decrease in *T*. On the other hand, *EM* values were kept constant along the whole association process since this parameter does not vary strongly with solvent composition.^35,69^ We thus employed the calculated *EM* value for *c*(**GC**)_4_ in THF (*EM*_GC_ = 1.2·10^2^ M), and an estimated value for *c*(**AU**)_4_ (*EM*_AU_ = 5·10^–4^ M), as explained above. The shadowed areas shown in each distribution profile correspond to the experimental concentration range employed in the ^1^H NMR (*ca*. 10^–2^–10^–4^ M; in light orange) and optical spectroscopy (*ca*. 10^–3^–10^–5^ M; in light violet) experiments. As it can be appreciated in Figure 6 by comparison of top and bottom horizontal panels, two very different supramolecular evolutions are obtained for **GC** and **AU** preceding the polymerization event.

For **GC** (top panel), *EM* values are extraordinarily large and the association between G:C pairs is relatively strong, which leads to a very strong chelate cooperativity and to an “*all-or-nothing*” situation: either a robust cyclic tetramer is formed or nothing else but the monomer survives in solution, so the participation of open G:C H-bonded oligomers is insignificant. As *K*_G:C_ is increased at higher *V*_hep_/lower *T*, the *c*(**GC**)_4_ population increases until this ring is formed quantitatively, as also evidenced experimentally. If polymerization is triggered at this point, the corresponding nuclei would be formed by stacked cyclic tetramers and the polymer is fed from these very stable, both thermodynamically and kinetically, macrocycles. This scenario would lead to *stacked* polymer nanotubes. This organization, as theory suggests, can reliably reproduce the experimental CD spectrum recorded for (**GC**)*_n_*.

For **AU** (bottom panel), on the contrary, *EM* values are more than 4 orders of magnitude smaller, whereas *K*_A:U_ is also significantly lower, which leads to a weak chelate cooperativity. Hence, as we increase *K*_A:U_ at higher *V*_hep_/lower *T*, open A:U H-bonded oligomers compete strongly with the *c*(**AU**)_4_ cyclic tetramer, and the latter is only formed in small amounts. Together with the **AU** monomer, these open oligomers are not active in CD nor provide ^1^H NMR signals in slow exchange, but their formation was experimentally proven by the downfield shift experienced by the relevant H-bonded ^1^H NMR probes. As we further increase *V*_hep_/decrease *T*, the population of relatively long A:U bound oligomers becomes larger. We thus assume that these oligomers might be able to adopt folded conformations, aided by additional π–π stacking and H-bonding interactions between peripheral amides. Please note that this extra stabilization enjoyed by folded oligomers is however not considered in the simulations of the speciation curves. Some of these stabilized folded species may then grow by incorporation of more **AU**, thus shifting all equilibria toward the formation of *folded* or *coiled* polymer nanotubes before cyclic species are even formed in significant amounts.

## Conclusions

We provided here an example of two structurally related molecules that exhibit very different noncovalent association scenarios prior to their supramolecular polymerization and, as a result, generate self-assembled nanotubes with distinct monomer arrangements: either stacked or coiled. These internal structures can, at the same time, define different helicities that result in opposite chiroptical properties, as determined by CD and CPL.

The molecules comprise a *S*-chiral π-conjugated central block substituted with complementary nucleobases at its termini: either guanine and cytosine (**GC**) or 2-aminoadenine and uracil (**AU**). The establishment of Watson–Crick interactions between the edges of these monomers led to a distribution of H-bonded oligomers among which a cyclic tetramer stands out as a significantly stabilized species, as long as chelate cooperativity is high enough. Such high cyclization cooperativities are attained by the **GC** monomer, which can quantitatively form cyclic tetramers, but not by the **AU** monomer, which instead associates preferentially in open oligomers. The evolution of these two distinct supramolecular scenarios as the association strength is increased by, for instance, increasing the volume fraction of apolar solvent can offer a solid explanation of the pathway, followed by each dinucleobase monomer to arrive to stacked or folded nanotubes. However, it must be remarked that we have also demonstrated that the final (**GC**)*_n_* and (**AU**)*_n_* aggregates do not show any further transformation under any conditions, meaning that the stacked or folded organizations are not kinetic intermediates, but actually thermodynamic products. Computational simulations strongly support this notion, and clearly show the resilience of **GC** to maintain the cyclic assemblies, and the tendency of **AU** to disrupt them and instead develop coiled structures. Therefore, the entropic factors that rule chelate cooperativity in these systems and hence the preference to associate as cyclic species (please, see our previous work) must also operate when the molecules aggregate in the final nanotube assemblies and make them remain associated as stacked macrocycles or as polymeric spirals. The results and main findings of this work can be helpful in the design of novel strategies aiming to control the structure and the function of synthetic self-assembled nanotubes that can mimic biological analogues.

## Supporting Information Available

Experimental procedures and compound characterization data, along with the supramolecular study by ^1^H NMR, supramolecular study by optical spectroscopy, microscopy characterization of the self-assembled nanotubes, self-sorting experiments, and theoretical calculations (Figures S1–S5) (PDF)
