# Supplementary material for: Stacked or Folded? Impact of Chelate Cooperativity on the Self-Assembly Pathway to Helical Nanotubes from Dinucleobase Monomers
Source: J Am Chem Soc. 2023 Aug 2;145(32):17805–18. doi: 10.1021/jacs.3c04773 (PMC10436278; doi:10.1021/jacs.3c04773)
Supplement: Supplementary file 1 — ja3c04773_si_001.pdf [file ja3c04773_si_001.pdf]

SUPPORTING INFORMATION

Stacked or Folded? Impact of Chelate Cooperativity on the Self-assembly Pathway to Helical Nanotubes from Dinucleobase Monomers

Marina González-Sánchez,[a] María J. Mayoral,[b] Violeta Vázquez-González,[a] Markéta Paloncýová,[c,d] Irene Sancho-Casado,[a] Fátima Aparicio,[a] Alberto de Juan,[a] Giovanna Longhi,[e] Patrick Norman,[c] Mathieu Linares,[f]* and David González-Rodríguez[a,g]*

[a] M. González-Sánchez, Dr. V. Vázquez-González, I. Sancho-Casado, Dr. F. Aparicio, Dr. A. de Juan, Prof. D. González-Rodríguez
Nanostructured Molecular Systems and Materials Group, Organic Chemistry Department, Science Faculty, Universidad Autónoma de Madrid, 28049 Madrid, Spain
E-mail: david.gonzalez.rodriguez@uam.es

[b] Dr. M. J. Mayoral
Department of Inorganic Chemistry, Facultad de Ciencias Químicas, Universidad Complutense de Madrid, 28040 Madrid, Spain

[c] Dr. M. Paloncýová, Prof. P. Norman
Division of Theoretical Chemistry and Biology, School of Engineering Sciences in Chemistry, Biotechnology and Health, KTH Royal Institute of Technology,SE-100 44 Stockholm, Sweden

[d] Dr. M. Paloncýová
Regional Centre of Advanced Technologies and Materials, Czech Advanced Technology and Research Institute (CATRIN), Palacký University Olomouc, 779 00 Olomouc, Czech Republic

[e] Prof. G. Longhi
Department of Molecular and Translational Medicine, University of Brescia, Viale Europa 11, 25123 Brescia, Italy

[f] Dr. M. Linares
Laboratory of Organic Electronics and Scientific Visualization Group, ITN, Campus Norrköping; Swedish e-Science Research Centre (SeRC), Linköping University, 58183 Linköping, Sweden
E-mail: mathieu.linares@liu.se

[g] Prof. D. González-Rodríguez
Institute for Advanced Research in Chemical Sciences (IAdChem), Universidad Autónoma de Madrid, 28049 Madrid, Spain

SUPPORTING INFORMATION

TABLE OF CONTENTS

S0. General Methods and Synthetic Procedures 2

S1. Supramolecular study by ^{1}H NMR 11

S2. Supramolecular Study by Optical Spectroscopy 16

- **S2.1. Denaturation Experiments in Solvent Mixtures** 16
- **S2.2. Temperature-dependent Experiments** 20
- **S2.3. Concentration-dependent Experiments** 26
- **S2.4. Circularly Polarized Luminescence (CPL) Experiments** 26

S3. Microscopy Characterization of the Self-assembled Nanotubes 28

S4. Self-sorting Experiments 31

S5. Theoretical Calculations 37

S0. General Methods and Synthetic Procedures

General Methods

Mass Spectrometry (MS) and **High Resolution-Mass Spectrometry (HRMS)** MALDI-TOF spectra were obtained from a BRUKER ULTRAFLEX III instrument equipped with a nitrogen laser operating at 337 nm. **NMR** spectra were recorded with a *BRUKER AVANCE-II* 300 MHz or a *BRUKER DRX* 500 MHz instrument. The temperature was actively controlled at 298 K. Chemical shifts are measured in ppm using the signals of the deuterated solvent as the internal standard [$CDCl_3$ calibrated at 7.26 ppm (^1H) and 75.0 ppm (^{13}C), DMSO-d_6 calibrated at 2.50 ppm (^1H) and 39.5 ppm (^{13}C), cyclohexene-d_{12} calibrated at 1.38 ppm (^1H) and 26.4 ppm (^{13}C) and THF-d_8 calibrated at 3.58 (^1H) and 39.5 ppm (^{13}C)]. **Column chromatography** was carried out on silica gel *Merck-60* (230-400 mesh, 60 Å), and TLC on aluminium sheets precoated with silica gel 60 F254 (Merck). **UV-Visible** experiments were conducted using a *JASCO V-660* apparatus. **Emission spectra** were recorded in a *JASCO FP-8600* equipment using excitation and emission bandwidths of 5 nm in both cases, and a 50 ms response. **CD spectra** were recorded with a *JASCO* J-815 equipment (measurement information: data pitch = 1 nm; sensitivity = standard; D.I.T. = 2 sec; slit width = 1000 um; data interval = 1 nm; scanning speed = 200 nm/min). In all these three instruments the temperature was controlled using a *JASCO* Peltier thermostatted cell holder with a range of 263–383 K, adjustable temperature slope, and accuracy of ± 0.1 K. **CPL spectra** have been recorded on a home-made apparatus[1] with 90° setup employing a fluorescence quartz cell with 2 mm-pathlength in the excitation side and with 10 mm-pathlength in the emission side. The excitation radiation was brought to the cell from a Jasco FP8200 fluorimeter through a water filled optical fiber, the incident radiation has been polarized parallel to the collection direction. **Transmission electron microscopy** (**TEM**) images were obtained with a *JEM 1400 K PLUS* instrument operating at 100 kV for non-stained samples. **Scanning Emission Microscopy** (**SEM**) images were obtained with a Philips XL30 S-FEG instrument. The **Hyss** (Hyperquad Simulation and Speciation) program, developed by http://www.hyperquad.co.uk, was used to generate the speciation plots shown in Figure 6 in the text. Effective molarity values of $EM_{GC} = 1.2 \cdot 10^2$ M and $EM_{AU} = 5 \cdot 10^{-4}$ M, and association constant values of $K_{G:C} = 4.1 \cdot 10^2$ M^{-1} and $K_{A:U} = 1.7 \cdot 10^1$ M^{-1} were employed in these simulations for $V_{hep} = 0$. To simulate the effect of increasing V_{hep}, the association constants were gradually increased arbitrarily, whereas the *EM* values remained constant.

Starting materials

Chemicals were purchased from commercial suppliers and used without further purification. Solid hygroscopic reagents were dried in a vacuum oven before use. Reaction solvents were thoroughly dried before use using standard methods. The synthetic procedures and characterization data of the dihalogenated central block *S*-**P**,[2] the *S*-**GC**[3] monomer (Scheme S0C), as well as intermediates **G**[4]

---

[1] Castiglioni, E.; Abbate, S.; Longhi, G. Revisiting with updated hardware an old spectroscopic technique: circularly polarized luminescence. *Appl. Spectrosc*. **2010**, *64*, 1416-1419.

[2] Montoro-García, C.; Mayoral, M. J.; Chamorro, R.; González-Rodríguez, D. How Large Can We Build a Cyclic Assembly? Impact of Ring Size on Chelate Cooperativity in Noncovalent Macrocyclizations. *Angew. Chem. Int. Ed.* **2017**, *56*, 15649-15653.

[3] Vázquez-González, V.; Mayoral, M. J.; Chamorro, R.; Hendrix, M. M. R. M.; Voets, I. K.; González-Rodríguez, D. Noncovalent Synthesis of Self-Assembled Nanotubes through Decoupled Hierarchical Cooperative Processes. *J. Am. Chem. Soc*. **2019**, *141*, 16432-16438.

[4] Bilbao, N.; Vázquez-González, V.; Aranda, M. T.; González-Rodríguez, D. Synthesis of 5-/8- Halogenated Lipophilic Nucleobases as Potential Synthetic Intermediates for Supramolecular Chemistry. *Eur. J. Org. Chem.* **2015**, *32*, 7160-7175.

(Scheme S0A), **C.1** (Scheme S0B),[4] **A.2**[5] (Scheme S0D) and *tert*-butyl(2-bromoethyl)carbamate[6] have been reported.

[5]Romero-Pérez, S.; López-Martín, I.; Martos-Maldonado, M. C.; Somoza, A.; González-Rodríguez, D. Synthesis of phosphoramidite monomers equipped with complementary bases for solid-phase DNA oligomerization. *Org. Lett.* **2020**, *22*, 41-45

[6] Borbas, K. E.; J. I. Bruce. Synthesis of asymmetrically substituted cyclen-based ligands for the controlled sensitisation of lanthanides *Org. Biomol. Chem.* **2007**, *5*, 2274–2282.

Synthetic Procedures

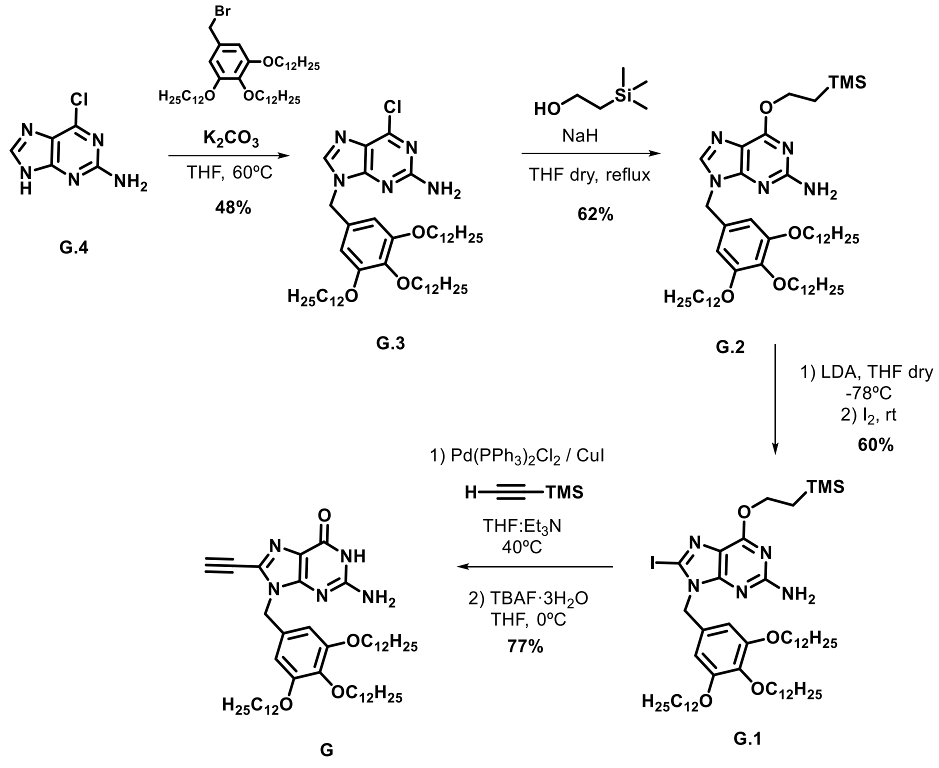

Scheme S0A. Synthesis of the 8-ethynyl-substituted purine nucleobase **G**.

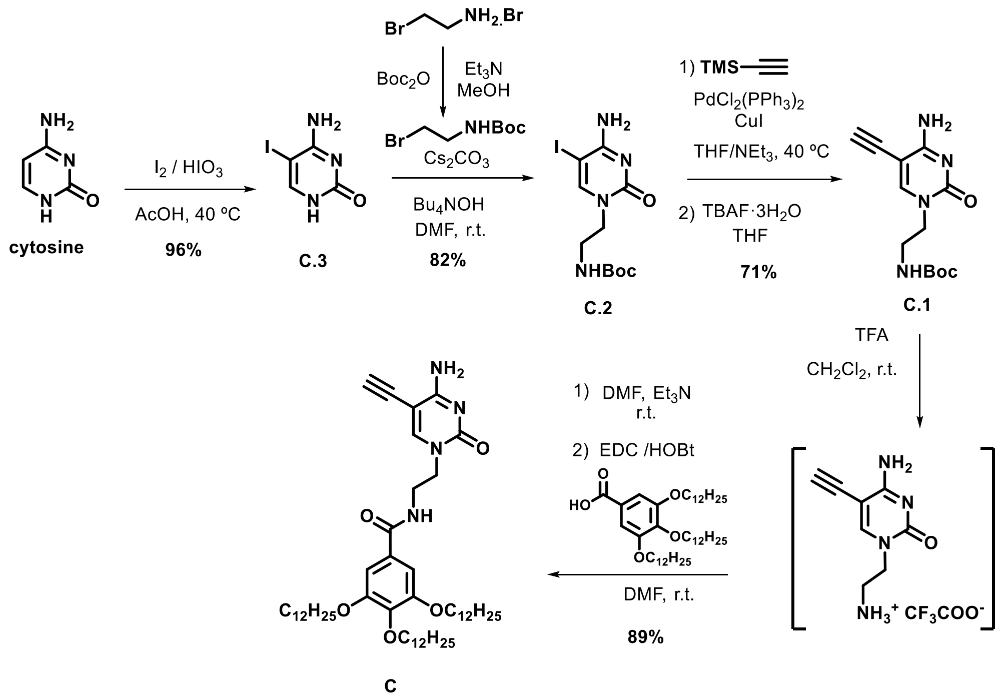

Scheme S0B. Synthesis of the 5-ethynyl-substituted pyrimidine nucleobase **C.**

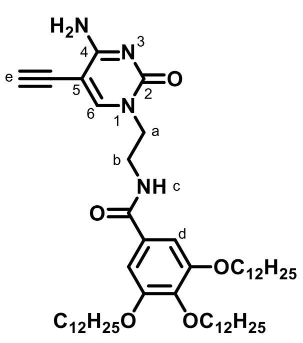

C. To a suspension of **C.1**[3] (500 mg, 1.8 mmol) in dry CH_2Cl_2 (20 mL), TFA (7.0 mL, 90 mmol) was added. The solution was stirred for 3 h at room temperature, then TFA was removed by coevaporation with the solvent and the crude product was re-dissolved in dry DMF (20 mL) and Et_3N (9.0 mL, 66 mmol) was added. In another flask, 3,4,5-tris(dodecyloxy)benzoic acid[7] (1.7 g, 2.5 mmol), EDC (964 mg, 5.0 mmol), HOBt (849 mg, 6.3 mmol) and DIPEA (0.9 mL, 5.0 mmol) were dissolved in dry CH_2Cl_2 (20 mL) and stirred at r.t. for 2 h. Then, the solvent was removed and DMF (20 mL) was added. The resulting solution was then added *via* cannula to the previous solution of the cytosine derivative. The mixture was stirred at r.t. overnight. Once the reaction was completed, the solvent was removed and the crude product was purified by column chromatography, using a mixture of $CHCl_3$:MeOH (50:1) as eluent. The product was precipitated in MeCN. **C** was obtained as a white solid (960 mg, 79%). **^{1}H NMR** (300 MHz, $CDCl_3$): δ= 7.66 (m, 1H, N*H*), 7.56 (s, 1H, H^6), 7.06 (s, 2H, H^d), 5.96 (s (broad), 1H, NH-*H*), 5.55 (s (broad), 1H, NH-*H*), 4.09 (t, *J* = 5.7 Hz, 2H, CH_2^b), 3.98 (m, 6H, OCH_2), 3.77 (t, *J* = 5.7 Hz, 2H, CH_2^a), 3.29 (s, 1H, H^e), 1.85-1.25 (m, 60H, $OCH_2(CH_2)_{10}CH_3$), 0.88 (t, *J* = 6.4 Hz, 9H, $O(CH_2)_{11}CH_3$) ppm. **^{13}C NMR** (75 MHz, $CDCl_3$): δ = 168.3, 164.9, 155.5, 153.1, 150.1, 141.1, 128.9, 106.0, 89.8, 84.2, 77.3, 74.6, 73.6, 69.3, 49.8, 45.0, 39.6, 32.0, 30.4, 29.8, 29.5, 26.3, 26.2, 22.8, 14.2 ppm. **HRMS** (MALDI$^+$: matrix: DCTB+PPG790+NaI): Calculated for $C_{51}H_{87}N_4O_5$ $[M+H]^+$ = 835.6671; Found: 835.6661

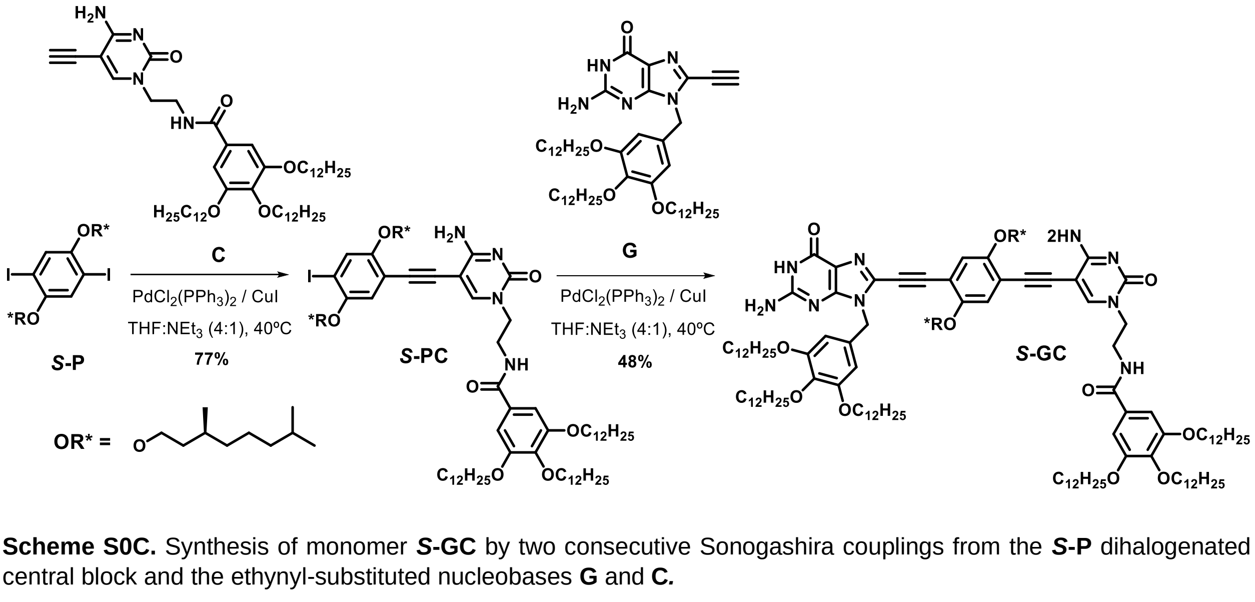

Scheme S0C. Synthesis of monomer ***S*-GC** by two consecutive Sonogashira couplings from the ***S*-P** dihalogenated central block and the ethynyl-substituted nucleobases **G** and **C.**

[7] C. V. Yelamaggad, R. Prabhu, D. S. S. Rao, S. K. Prasad, *Tetrahedron Lett.* **2010**, 51, 4579-4583.

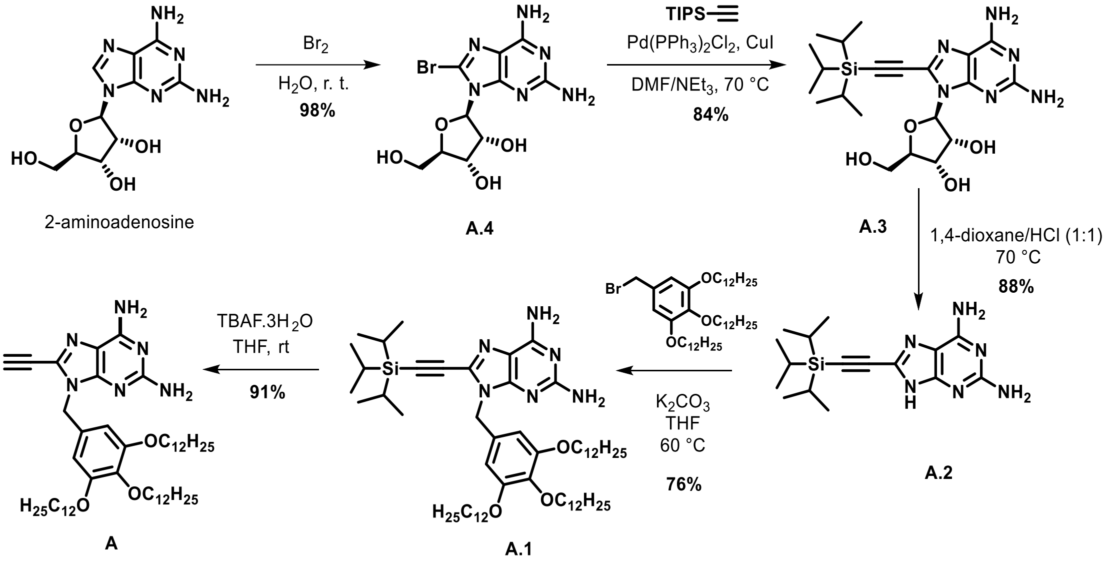

Scheme S0D. Synthesis of the 8-ethynyl-asubstituted purine nucleobase **A.**

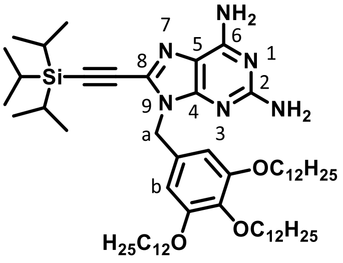

A.1. A solution of **A.2**[5] (700 mg, 2.1 mmol) and K_2CO_3 (292 mg, 2.1 mmol) in dry THF (20 mL) was stirred at r.t. for 1 h, then the 3,4,5-tris(dodecyloxy)benzyl bromide[8] (463 mg, 2.1 mmol) was added. The resulting mixture was stirred at 60 °C under argon atmosphere overnight. Once the reaction was completed, the solvent was evaporated under reduced pressure and the resulting solid was purified by column chromatography, using $CHCl_3$:MeOH (100:1) as eluent. **A.1** was obtained as a pale solid (1.57 g, 76%). **^{1}H NMR** (300 MHz, $CDCl_3$): δ = 6.47 (s, 2H, H^b), 5.61 (s (broad), 2H, N*H$_2$*), 5.22 (s, 2H, CH_2^a), 4.85 (s (broad), 2H, N*H$_2$*), 3.86 (m, 6H, OC*H$_2$*), 1.72 (m, 6H, OCH_2CH_2), 1.45-1.25 (m, 54H, $OCH_2CH_2(CH_2)_9CH_3$), 1.15–1.00 (m, 21H, $Si(CH(CH_3)_2)_3$), 0.95–0.80 (m, 9H, $O(CH_2)_{11}CH_3$) ppm. **^{13}C NMR** (75 MHz, $CDCl_3$): δ = 160.9, 156.0, 153.4, 152.0, 137.9, 132.2, 131.2, 114.3, 106.0, 98.5, 95.6, 73.5, 69.3, 46.6, 36.5, 32.0, 30.4, 29.9, 29.84, 29.82, 29.79, 29.77, 29.75, 29.72, 29.54, 29.52, 29.49, 29.47, 26.3, 26.2, 22.8, 18.7, 14.2, 11.3. **MS** (FAB^+): Calculated for $C_{59}H_{105}N_6O_3Si$ $[M+H]^+$: 972.5; Found: 973.5

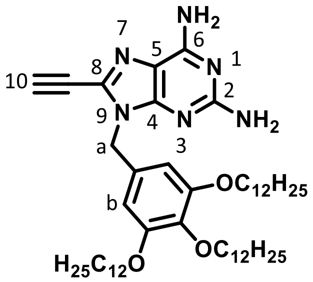

A. To a solution of **A.1** (1.50 g, 1.5 mmol) in THF (25 mL) at r.t., TBAF·3H_2O (729 mg, 2.3 mmol) was added. After 3 h, the reaction was completed. The solvent was evaporated under reduced pressure and the residue was purified by column chromatography, using as eluent a mixture of $CHCl_3$:MeOH (100:1). The product was precipitated in MeCN to obtain **A** as a white solid (1.15 g, 91%). **^{1}H NMR** (300 MHz, $CDCl_3$): δ = 6.59 (s, 2H, H^b), 5.35 (s (broad), 2H, N*H$_2$*), 5.18 (s, 2H, CH_2^a), 4.74 (s (broad), 2H, N*H$_2$*), 3.90 (m, 6H, OC*H$_2$*), 3.41

[8] L. Lee, Y. Zao, *J. Am. Chem. Soc.* **2014**, 136, 5579–5582.

(s, 1H, C≡CH^{10}), 1.85–1.60 (m, 6H, OCH$_2$CH_2), 1.50–1.20 (m, 54H, OCH$_2$CH$_2$(CH_2)$_9$CH$_3$), 0.88 (t, *J* = 7.0, 6.3 Hz, 9H, O(CH$_2$)$_{11}$CH_3) ppm. **^{13}C NMR** (75 MHz, CDCl$_3$): δ = 160.9, 155.9, 153.3, 152.0, 138.1, 131.1, 114.5, 106.8, 82.6, 73.9, 73.5, 69.3, 46.8, 32.0, 30.4, 29.9, 29.8, 29.8, 29.8, 29.7, 29.6, 29.5, 26.2, 22.8, 14.2 ppm. **HRMS** (MALDI$^+$, matrix: DCTB+NaI): calculated for $C_{50}H_{84}N_6NaO_3$ [M+Na]$^+$: 839.6497; Found 839.6478

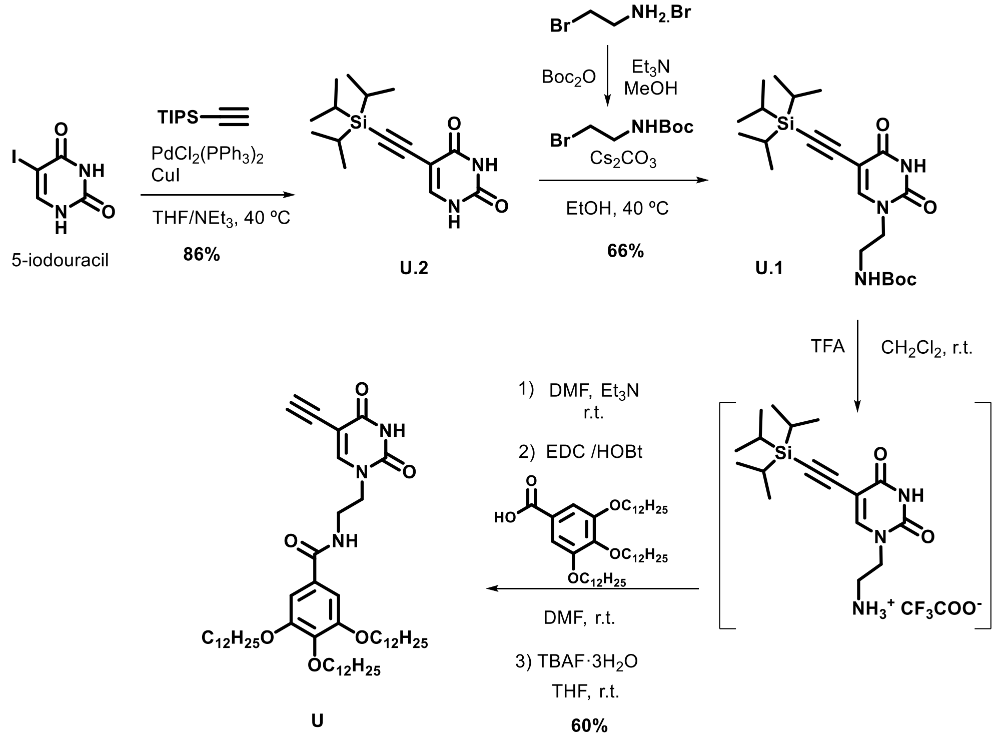

Scheme S0E. Synthesis of the 5-ethynyl-substituted pyrimidine nucleobase **U.**

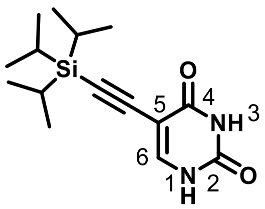

U.2 was prepared dissolving 5-iodouracil (20.0 g, 84.0 mmol), Pd(PPh$_3$)$_2$Cl$_2$ (1.18 g, 1.7 mmol) and CuI (160 mg, 0.8 mmol) in the solvent mixture THF/NEt$_3$ (4:1, 60 mL), which was previously subjected to deoxygenation by three freeze-pump-thaw cycles with argon. TIPSA (21.6 mL, 84.04 mmol) was then added and the reaction mixture was stirred at 40 °C under argon atmosphere overnight. Once the reaction was complete, after solvent removal, was added CHCl$_3$ and the solid was filtered and washed with cold H$_2$O and cold CHCl$_3$ to obtain a white solid (21.93 g, 86%). **^{1}H NMR** (300 MHz, DMSO-*d^6*): *δ* = 11.26 (m, 2H, NH^3, NH^1), 7.75 (s, 1H, H^6), 1.10–1.05 (m, 21H, Si(CH(CH_3)$_2$)$_3$) ppm. **^{13}C-NMR** (75 MHz, DMSO-*d^6*) δ = 162.9, 150.8, 146.8, 100.2, 97.6, 92.9, 18.9, 11.2 ppm. **HRMS** (MALDI$^+$, matrix: DCTB+NaI): calculated for $C_{15}H_{24}N_2O_2SiNa$ [M+Na]$^+$: 315.43; Found 315.20

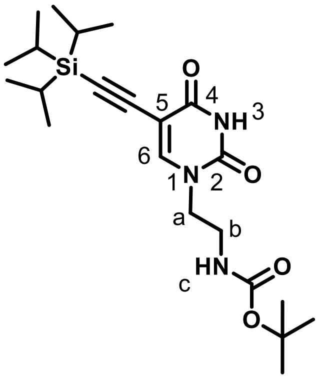

U.1. A solution of **U.2** (1.80 g, 6.1 mmol) and Cs_2CO_3 (2.41 g, 7.4 mmol) in EtOH (60 mL) was stirred at 40 ºC for 30 min. Later, *tert*-butyl(2-bromoethyl)carbamate (1.38 g, 6.1 mmol) was added and stirred at 40 ºC overnight. After the reaction was completed, the solvent was removed under reduced pressure. The crude was re-dissolved in ethyl acetate and was neutralised with HCl 2M until pH=7. The organic phase was separated and was removed under reduced pressure. The resulting crude was purified by column chromatography eluted with $CHCl_3$/MeOH a mixture (100:1), affording the product as a white solid (1.77 g, 66%). **^{1}H NMR** (300 MHz, $CDCl_3$): δ = 7.35 (s, 1H, H^6), 5.22 (m, 1H, NH^c), 3.83 (m, 2H, CH_2^b), 3.32 (m, 2H, CH_2^a), 1.32 (m, 9H (CH_3)$_3$), 1.05–1.00 (m, 21H, Si(CH(CH_3)$_2$)$_3$) ppm. **^{13}C NMR** (75 MHz, $CDCl_3$) δ = 162.1, 156.6, 150.5, 148.3, 100.9, 97.3, 80.7, 77.7 49.4, 39.6, 28.8, 21.2, 13.6 ppm. **HRMS** (MALDI$^+$, matrix: DCTB+NaI): calculated for $C_{22}H_{37}N_3O_4SiNa$ $[M+Na]^+$: 458.6; Found 458.4

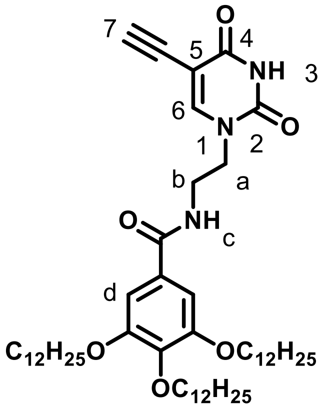

U To a suspension of **U.1** (1.0 g, 2.3 mmol) in CH_2Cl_2 (35 mL), TFA (10 mL) was added. The solution was stirred for 3 h at room temperature. Then, TFA was removed by several co-evaporations with the solvent and the crude product was re-dissolved in dry DMF (15 mL) and Et_3N (5 mL) was added. In another flask, the 3,4,5-tris(dodecyloxy)benzoic acid (2.17 g, 3.2 mmol) was dissolved in CH_2Cl_2 (40 mL) was stirred at 0ºC. Then was added EDC (998 mg, 6.4 mmol), HOBt (1.09 g, 8.0 mmol) and DIPEA (1.1 mL, 6.4 mmol) and stirred at 0 ºC for 10 min and later stirred at r.t for 1.5 h. Then, the solvent was removed and DMF (25 mL) was added, the resulting solution was then added *via cannula* to the previous solution of the deprotected **U.2**. The resulting mixture was stirred at r.t. overnight. Once the reaction was completed, the solvent was removed and the resulting crude in THF (25 mL) at r.t., TBAF·3H_2O (715 mg, 2.3 mmol) was added. After 3 h, the reaction was completed. The solvent was evaporated under reduced pressure and the residue was purified by column chromatography, using as eluent a mixture of $CHCl_3$:MeOH (100:1). The product was precipitated as a white solid (1.14 g, 60%). **^{1}H NMR** (300 MHz, $CDCl_3$): δ = 10.55 (s (broad), 1H, NH^3), 7.66 (s (broad), 1H, NH^c), 7.50 (s, 1H, H^6), 7.07 (s, 2H, CH_2^d), 3.95 (m, 8H, OCH_2, CH_2^b), 3.70 (m, 2H, CH_2^a), 2.88 (s, 1H, C≡CH^7), 1.90–1.70 (m, 6H, OCH$_2$CH_2), 1.50–1.15 (m, 54H, OCH$_2$CH$_2$(CH_2)$_9$CH$_3$), 0.86 (t, J = 6.6 Hz, 9H, O(CH$_2$)$_{11}$CH_3) ppm. **^{13}C NMR** (75 MHz, $CDCl_3$): δ = 168.5, 162.9, 153.11, 153.06, 150.8, 149.4, 141.3, 128.6, 106.1, 98.9, 82.6, 74.2, 73.6, 69.4, 49.2, 39.1, 32.1, 32.0, 30.5, 29.9, 29.85, 29.80, 29.75, 29.6, 29.5, 29.4, 26.3, 26.2, 22.8, 22.7, 14.2, 14.1 ppm. **MS** (FAB$^+$): Calculated for $C_{51}H_{86}N_3O_6$ $[M+H]^+$: 836.7; Found: 836.0

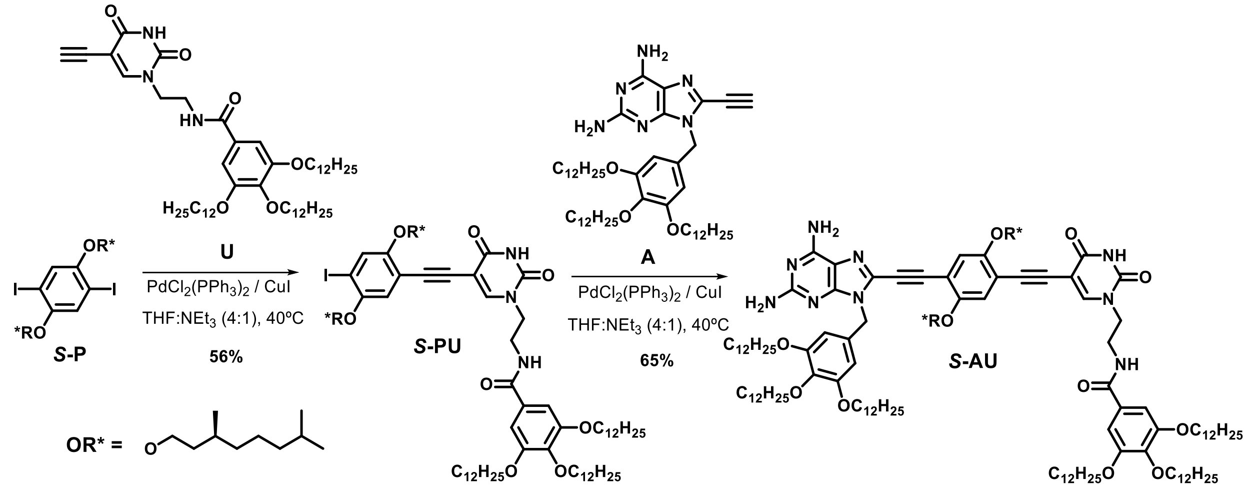

Scheme S0F. Synthesis of monomer ***S*-AU** by two consecutive Sonogashira couplings from the ***S*-P** dihalogenated central block and ethynyl-substituted nucleobases **A** and **U*****.***

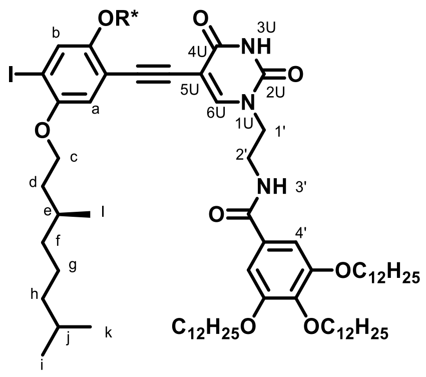

***S*-PU. U** (200 mg, 0.2 mmol), ***S*-P** (629 mg, 1.0 mmol), $Pd(PPh_3)_2Cl_2$ (4 mg, 0.01 mmol) and CuI (1 mg, 0.005 mmol) were dissolved in a mixture of THF:Et_3N (4:1) (25 mL), which was previously subjected to deoxygenation by three freeze-pump-thaw cycles with argon. The resulting solution was stirred under argon at 40 ºC overnight. Then, the solvent was removed under reduced pressure. The crude product was purified by column chromatography, using as eluent a $CHCl_3$:MeOH (20:1) mixture, affording the product as a yellow oil (182 mg, 56%). **^{1}H NMR** (300 MHz, $CDCl_3$) δ = 8.52 (s (broad), 1H, NH^{3U}), 7.46 (s, 1H, H^{6U}), 6.89 (m, 3H, $H^{4'}$, H^a/H^b), 6.89 (s, 1H, H^b/H^a), 6.74 (s (broad), 1H, N$H^{3'}$) 3.91 (m, 12H, OCH_2, N-$CH_2{}^{1'}$), 3.65 (m, 2H, $CH_2{}^{2'}$-NH), 1.85 – 1.05 (m, 80H, OCH_2CH_2, $CH_2{}^d$, $OCH_2CH_2(CH_2)_9CH_3$, $CH_2{}^{f,g,h}$, $H^{e,j}$), 0.90-0.75 (m, 27H, $O(CH_2)_{11}CH_3$, $CH_3{}^{i,k,l}$). **^{13}C NMR** (75 MHz, $CDCl_3$) δ = 180.8, 169.8, 165.9, 153.5, 152.2, 150.7, 150.2, 145.2, 144.5, 141.5, 138.2, 129.4, 129.0, 128.6, 123.0, 118.1, 106.3, 105.9, 105.4, 101.9, 73.7, 71.9, 69.2, 66.1, 53.3, 50.6, 50.1, 32.5, 31.1, 30.2, 30.0, 29.8, 26.6, 23.7, 14.6 ppm. **HRMS** ($MALDI^+$, matrix: DCTB+NaI): Calculated for $C_{77}H_{128}IN_3O_8Na$ $[M+Na]^+$: 1372.8597; Found: 1372.8654

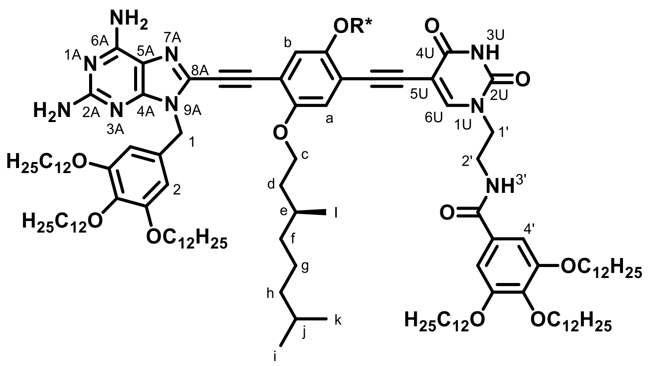

***S*-AU.** ***S*-PU** (100 mg, 0.07 mmol), **A** (63 mg, 0.07 mmol), $Pd(PPh_3)_2Cl_2$ (1 mg, 0.003 mmol) and CuI (0.3 mg, 0.001 mmol) were dissolved in a THF:Et_3N mixture (15 mL) and stirred at 40 ºC overnight. Once the reaction was completed, the solvent was removed under reduced pressure. The residue was purified by column chromatography, a $CHCl_3$:MeOH (100:1) mixture using as eluent. The product was precipitated in MeCN as a yellow solid (98 mg, 65%). **^{1}H NMR** (300 MHz, $CDCl_3$): δ = 10.85 (s, 1H, H^{3U}), 7.61 (s, 1H, H^{6U}), 7.05 (s, 1H, H^a/ H^b), 7.01 (s, 1H, H^b/ H^a), 6.94 (s, 2H, H^2), 6.86 (s, 2H, $H^{4'}$), 6.52 (s (broad), 1H, $NH^{3'}$), 5.87 (s (broad), 2H, NH_2), 5.25 (m, 4H, CH_2^{1}, NH_2), 4.0-3.75 (m, 20H, CH_2^{c}, OCH_2, C$H_2^{1'}$, C$H_2^{2'}$), 1.75-0.8 (m, 176H, OCH_2CH_2, CH_2^{d}, $OCH_2CH_2(CH_2)_9CH_3$, C$H_2^{f,g,h}$, $H^{e,j}$, $O(CH_2)_{11}CH_3$, C$H_3^{i,k,l}$) ppm. **^{13}C NMR** (75 MHz, $CDCl_3$) δ = 168.1, 162.9, 162.8, 157.9, 155.2, 155.1, 154.5, 154.4, 153.3, 152.1, 149.1, 142.5, 139.3, 133.6, 131.8, 131.0, 118.2, 116.9, 116.3, 116.2 113.3, 110.7, 108.8, 107.3, 100.6, 91.6, 90.5, 88.9, 87.3, 74.1, 73.9, 70.3, 69.9, 69.3, 69.0, 49.8, 47.6, 40.7, 39.6, 38.7, 37.8, 37.6, 33.3, 31.8, 31.2, 30.9, 30.8, 29.4, 29.3, 27.6, 24.0, 23.6, 23.5, 20.6, 20.3 ppm. **HRMS** (MALDI$^+$, matrix: DCTB): Calculated for $C_{127}H_{211}N_9O_{11}$ $[M]^+$: 2038.6; Found: 2038.6

S1. Supramolecular Study by ^{1}H NMR

The self-assembly of molecule **GC** was preliminarily studied in recent work,[3] and it is examined here in more detail. Qualitatively, **GC** showed the same *"all-or-nothing"* self-association characteristics than previous G-C monomers studied in the group,[9,10] and cyclic tetramers could be formed quantitatively in apolar solvents. As a consequence of the extraordinarily high chelate cooperativities attained for these monomers, the macrocycle is detected in slow NMR exchange with the monomer, without detectable participation of any other associated species (Figure S1Aa).[10] THF was the most adequate solvent to observe this monomer cyclic tetramer equilibrium within the 10^{-2}-10^{-4} M concentration range (Figure S1Aa). The association constant between G and C nucleobases was calculated in this solvent as: $K_{G:C}$ = 1.5·10^3 M^{-1}, while *EM* for *c*(**GC**)$_4$ was calculated as: *EM* = 1.2·10^2 M.[11]

Due to the lower binding constant between A and U nucleobases, as specified in the main text, less polar solvents, like $CHCl_3$ or toluene, are required to detect A:U association within the NMR concentration range. Here, in contrast to **GC**, ^{1}H NMR temperature-dependent experiments of **AU** in $CDCl_3$ (Figure S1Ab) revealed that the monomer established an equilibrium with open H-bonded oligomers, and no sign of the cyclic tetramer in slow NMR exchange was found, even if the concentration was increased (no higher than 10^{-2} M, due to solubility problems) or decreased (no lower than 10^{-4} M due to the detection limit of the ^{1}H NMR technique). As shown in Figure S1Ab, the spectra recorded at high temperatures revealed a single set of sharp proton signals and a U-imide proton at around 10 ppm, that is consistent with the presence of monomeric species. As the temperature is reduced, the U-imide proton shifted downfield, indicating a higher involvement in H-bonding as open oligomers are formed. At even lower temperatures, all proton signals became broader and eventually disappeared, suggesting the formation of larger aggregated species.

The fact that we could not detect the *c*(**AU**)$_4$ species in these temperature-dependent experiments clearly confirmed that the chelate cooperativity of this macrocycle is low enough so as to not assemble in significant amounts in $CDCl_3$. In order to try to increase the stability of this cyclic species, and thereof its population in solution, chelate cooperativity must be enhanced. We could do this by increasing *EM*, but this parameter depends mainly on molecular structure. Alternatively, we can increase $K_{A:U}$ between nucleobases by reducing solvent polarity. This could be done, as shown in the main text, by progressively increasing the volume fraction of cyclohexane-D_{12} (V_{cy}) in THF-D_8 (Figure S1B). For **GC** (Figure S1Ba), an equilibrium between monomer **GC** and cyclic tetramer *c*(**GC**)$_4$, in slow exchange at the NMR timescale, is detected. As V_{cy} increases, this equilibrium is shifted to the macrocycle side until the *c*(**GC**)$_4$ signals broaden and disappear due to supramolecular polymerization. For **AU** (Figure S1Bb), in contrast, a single set of proton signals was again detected along the whole V_{cy} range. As seen in temperature-dependent experiments in $CDCl_3$, the H-bonded A and U protons shift downfield upon increasing V_{cy}, due again to their

[9] Montoro-García, C.; Camacho-García, J.; López-Pérez, A. M.; Mayoral, M. J.; Bilbao, N.; González-Rodríguez, D. Role of the Symmetry of Multipoint Hydrogen Bonding on Chelate Cooperativity in Supramolecular Macrocyclization Processes. *Angew. Chem. Int. Ed.* **2016**, *55*, 223-227.

[10] Montoro-García, C.; Camacho-García, J.; López-Pérez, A. M.; Bilbao, N.; Romero-Pérez, S.; Mayoral, M. J.; González-Rodríguez, D. High-fidelity Noncovalent Synthesis of Hydrogen-bonded Macrocyclic Assemblies. *Angew. Chem. Int. Ed.* **2015**, *54*, 6780-6784.

[11] V. Vázquez-González, M. J. Mayoral, F. Aparicio, P. Martínez-Arjona, D. González-Rodríguez, The Role of Peripheral Amide Groups as Hydrogen-Bonding Directors in the Tubular Self-Assembly of Dinucleobase Monomers *ChemPlusChem* **2021**, 86, 1087–1096

higher involvement in H-bonding, but a cyclic species in slow exchange is not detected. Just like for **GC**, at very high V_{cy} the **AU** signals broaden and eventually disappear due to polymerization.

The association constant between nucleobases can also be increased using toluene as a solvent, as demonstrated recently.[12,13] Therefore, we next examined temperature-dependent experiments in toluene-D_8 at low concentrations ($6.0 \cdot 10^{-4}$ M; Figure S1C). In these conditions, **GC** is strongly associated as cyclic tetramers and only at high temperatures, signs of partial dissociation are noted (Figure S1Ca). The typical H-bonded G-amide and C-amine proton signals are found around 13.5 and 10.5 ppm, respectively, as also noted in THF-D_8 (Figure S1A) and in mixtures with cyclohexane-D_{12} (Figure S1B). On the other hand, as shown in Figure S1Cb for **AU** and as was also observed in $CDCl_3$, as the temperature decreases the protons involved in U-imide and A-amine protons involved in H-bonding shift downfield due to the oligomerization process. The peripheral amide proton at the U base also suffers a downfield shift at low temperatures, but less pronounced. Interestingly, and contrarily to GC, at the lowest temperatures the **AU** signals broaden and eventually disappear, which is attributed to polymerization into larger structures and is in line with optical spectroscopy experiments performed in the same conditions (see Section S2.2). In any case, no sign of cyclic *c*(**AU**)$_4$ species could be obtained in toluene, and the concentration could not be increased further due to sample precipitation.

In short, all NMR experiments performed in the 10^{-1}-10^{-4} M NMR regime in different solvent environments indicated a very different supramolecular behavior of **GC** and **AU** monomers. Specifically, for compound **AU**, we corroborated that THF is a too polar medium to detect any association between nucleobases, while $CHCl_3$ or toluene are more convenient solvents to observe monomer and associated species in equilibrium. However, the *c*(**AU**)$_4$ macrocycle could not be detected in any of the experiments.

[12] Mayoral, M. J.; Serrano-Molina, D.; Camacho-García, J.; Magdalena-Estirado, E.; Blanco-Lomas, M.; Fadaei, E.; González-Rodríguez, D. Understanding Complex Supramolecular Landscapes: Non-covalent Macrocyclization Equilibria Examined by Fluorescence Resonance Energy Transfer. *Chem. Sci*. **2018**, 9, 7809-7821.

[13] Mayoral, M. J.; Camacho-García, J.; Magdalena-Estirado, E.; Blanco-Lomas, M.; Fadaei, E.; Montoro-García, C.; González-Rodríguez, D. Dye-conjugated complementary lipophilic nucleosides as useful probes to study association processes by fluorescence resonance energy transfer. *Org. Biomol. Chem*. **2017**, 15, 7558-7565.

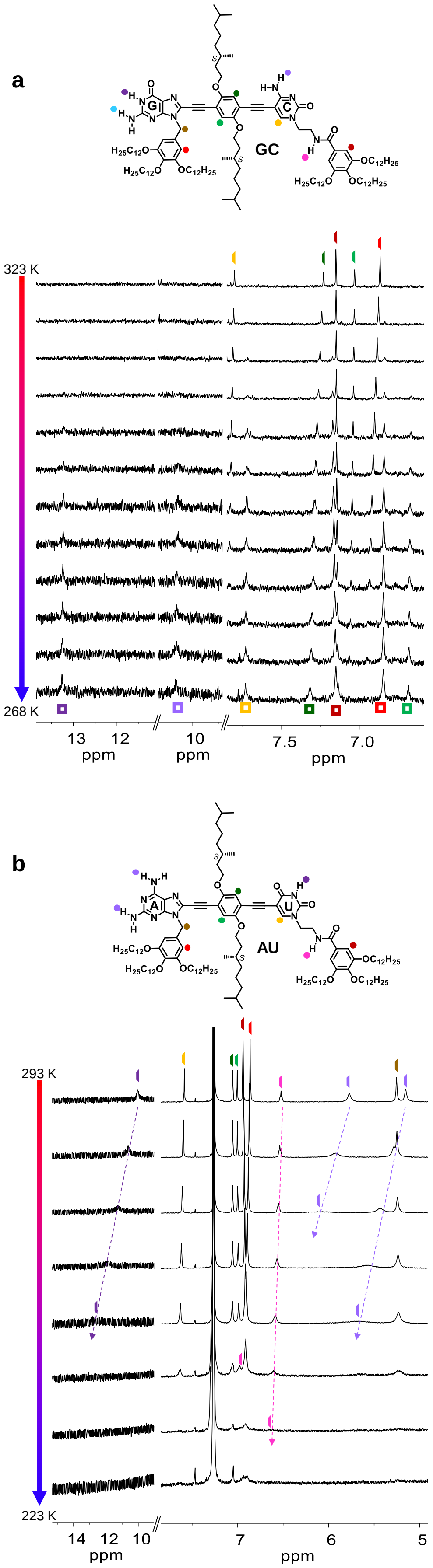

Figure S1A. ^{1}H-NMR temperature-dependent experiments of (**a**) **GC** in THF-*D*$_8$ at $2.0\cdot10^{-3}$ M and (**b**) **AU** in $CDCl_3$ at $1.0\cdot10^{-3}$ M.

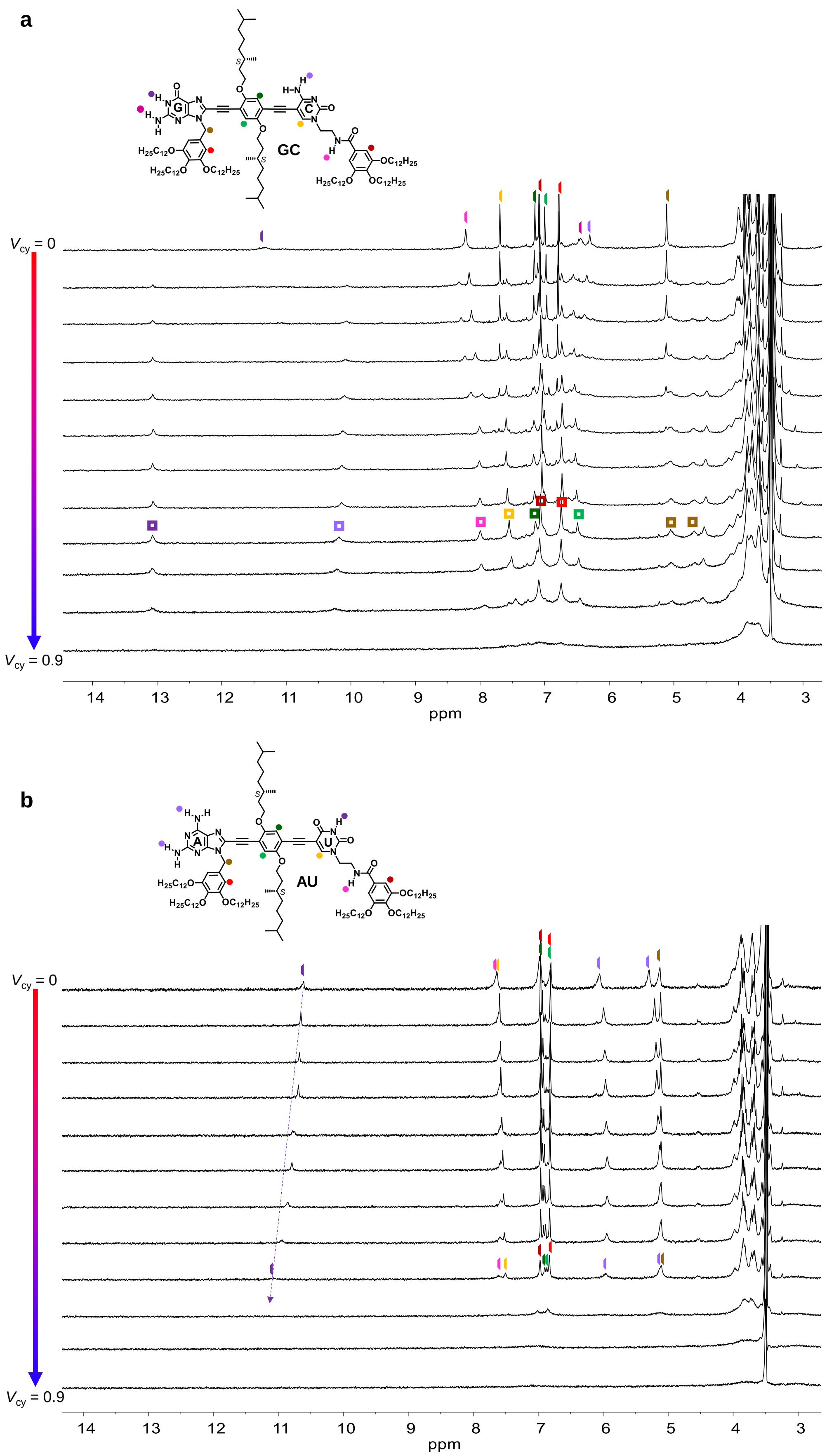

Figure S1B. Self-assembly of **GC** (**a**) and **AU** (**b**) by progressively increasing the volume fraction of cyclohexane-D_{12} (V_{cy}) in mixtures with THF-D_8 monitored by ^{1}H NMR. Rod-shaped marks correspond to monomeric or linear oligomeric species, while square-shaped marks correspond to cyclic tetramers.

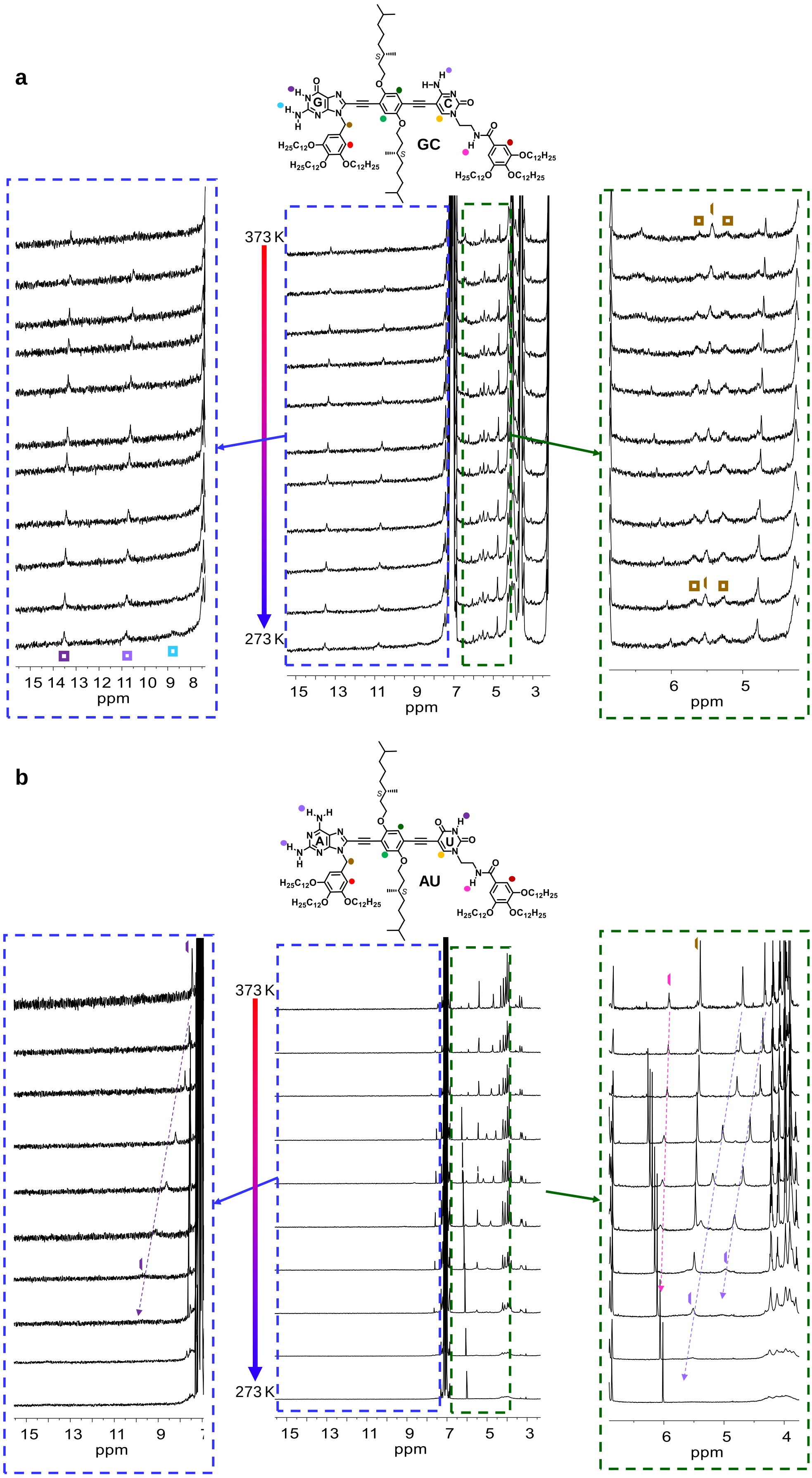

Figure S1C. ^{1}H-NMR temperature-dependent experiments of (**a**) **GC** and (**b**) **AU** in in toluene-D_8 at $6.0{\cdot}10^{-4}$ M.

S2. Supramolecular Study by Optical Spectroscopy

S2.1. Denaturation Experiments in Solvent Mixtures

We then proceeded to explore lower concentration ranges ($6.0 \cdot 10^{-4}$ – 10^{-6} M) and more sensitive optical spectroscopy techniques (CD, absorption or emission) to study the association of *S*-chiral compounds **GC** and **AU**. As already optimized in our previous work,[3] a convenient manner to enhance monomer association gradually is to work with THF:heptane solvent mixtures. In 100% THF both **GC** and **AU** are present in their monomer form at these low concentrations. Thus, increasing the volume fraction of the nonpolar heptane solvent (V_{hep}) in this mixture enhances intermolecular interactions until reaching the maximum degree of association at 100% heptane (V_{hep} = 1). In these strongly apolar conditions, however, both **GC** and **AU** solutions become turbid with time and eventually the aggregates precipitate, so a small amount of THF is always desirable to keep the aggregates in solution.

Let us first describe what happened when this gradual decrease in solvent polarity was applied to **GC**.[3] As V_{hep} is increased, the spectroscopic features that characterize the cyclotetramerization process are first observed (see for instance Figure 2b-d in the main text; red to green spectra): a CD signal appears, a red-shifted absorption shoulder, and also a red shift and decrease in emission intensity as the heptane content is increased. All these spectroscopic changes occurring along cyclotetramerization take place through clear isosbestic points and can also be observed as a function of temperature in 100% THF, thus matching the ^{1}H NMR spectra shown in Figure S1A (please see our previous work).[3] Further increasing V_{hep} up to 0.6-0.9 (depending on concentration; see below), produced no spectroscopic change, indicating that the *c*(**GC**)$_4$ species is quantitatively stabilized in solution, as it is complementarily demonstrated by ^{1}H NMR (see Figure S1B). However, at even higher V_{hep}, a distinct additional aggregation process is clearly detected (green to blue spectra in Figures 2b-d in the main text): an additional absorption red-shift to 425 nm is observed, while emission is further quenched and slightly blue-shifted. However, the most remarkable changes were detected in the CD measurements: at high V_{hep}, the *c*(**GC**)$_4$ CD features evolve with clear isosbestic points to a new signal exhibiting a Cotton effect at 428 nm, that is positive for *S*-**GC** and negative for *R*-**GC** (see our previous work).[3] This second sharp transition was attributed to the supramolecular polymerization process to yield (*c***GC**$_4$)$_n$, which would be driven by π–π stacking interactions between the large π-conjugated surface generated upon cyclization and by H-bonding interactions between the four peripheral amides.

A similar protocol was now applied to the *S*-chiral compound **AU**: solutions of **AU** in pure THF were titrated with solutions at the same concentration in a 0.01:0.99 THF-heptane mixture (see below), so we recorded the whole aggregation process (see for instance Figures 2b'-d' in the main text; red to blue spectra). The contrary additions were also executed, so as to record the disaggregation or denaturation process and to look for possible intermediates. Both experiments were performed at different overall concentrations (Figure S2A). In contrast to **GC**, the **AU** samples below V_{hep} = 0.8 are CD silent at all concentrations. No relevant absorption or emission changes were seen either within this range of solvent polarity, which corroborates that the cyclic tetramer is not formed in significant amounts. Now, above V_{hep} = 0.8, a bisignated CD signal with a negative Cotton effect is observed (Figure S2A). In addition, the absorption spectra revealed a marked red shift of about 36 nm and the emission band decreased as the

content of THF (V_{THF} = 1- V_{hep}) increased in the mixture (see Figures 2b'-d' in the main text). Moreover, both aggregation (increasing V_{hep}) and disaggregation (increasing V_{THF}) experiments are virtually superimposable. These results bring a first important difference between **GC** and **AU**: a single supramolecular process, where no self-assembled intermediates are detected by any technique, is always clearly observed for **AU**. Similar to what was seen with **GC**, the **AU** samples at (or very close to) V_{hep} = 1.0 tend to precipitate over time (in a timescale from hours to days, depending mostly on concentration), which, together with the NMR results (see Figure S1B), is a good indication for the formation of large polymeric aggregates at high V_{hep}.

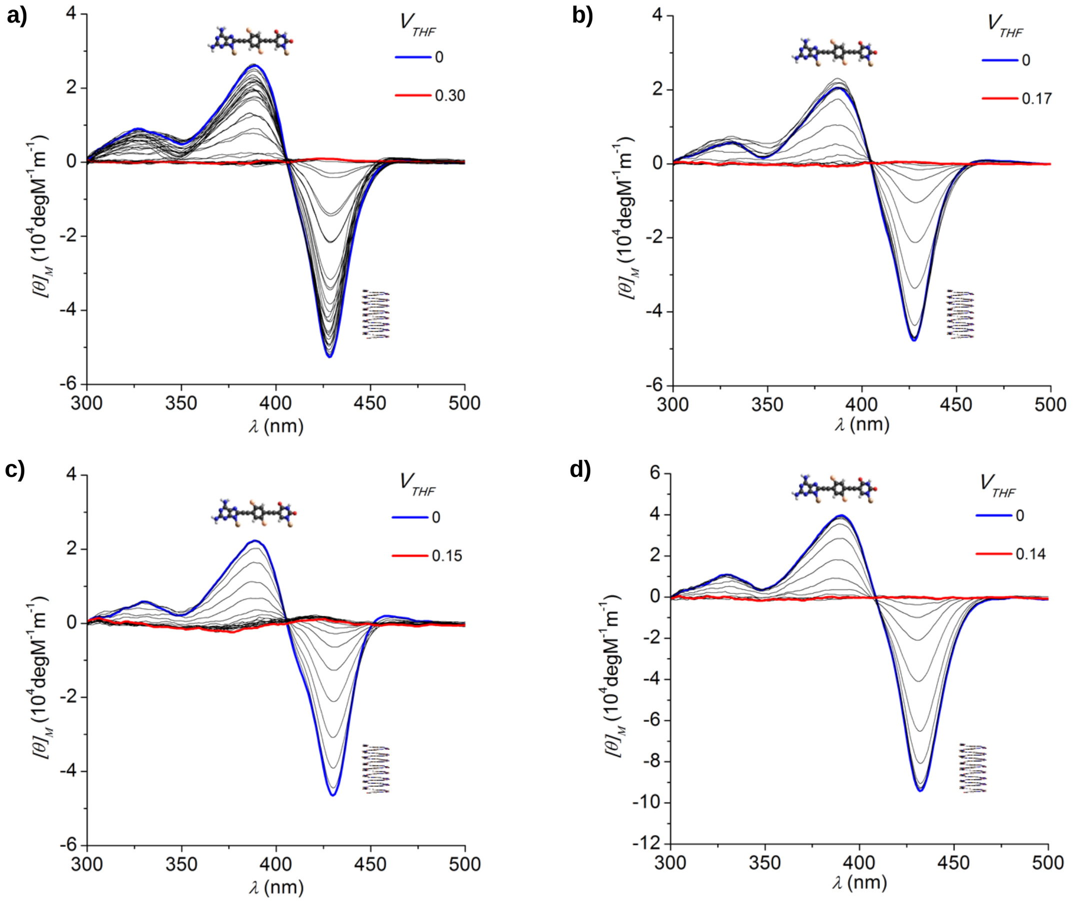

Figure S2A. CD changes experienced by **AU** upon increasing the denaturant volume fraction (V_{THF}) in THF-heptane mixtures at a constant concentration of (**a**) $6.0{\cdot}10^{-4}$ M, (**b**) $6.0{\cdot}10^{-5}$ M, (**c**) $3.0{\cdot}10^{-5}$ M and (**d**) $1.5{\cdot}10^{-5}$ M.

The trends recorded by monitoring the evolution of the **GC**/**AU** CD features as a function of V_{hep} at different concentrations are displayed in Figure 2e in the main text. For **GC**, both self-assembly stages, cyclotetramerization and polymerization, are clearly distinguished. It is clear that both processes are strongly dependent on the overall concentration. For instance, at $3.0{\cdot}10^{-4}$ M, we mainly start with a **GC**-*c*(**GC**)$_4$ equilibrium that is shifted to the cyclic species up to V_{hep} = 0.3, while the polymerization transition is detected above V_{hep} = 0.65. At the lower concentration of $1.0{\cdot}10^{-5}$ M, in contrast, we observe the whole two-step self-assembly process: from the monomer in pure THF, to the *c*(**GC**)$_4$ cycle within the V_{hep} = 0.5-0.9 plateau, and then to the polymer above V_{hep} = 0.95. In contrast to **GC**, the presence of a single polymerization process for **AU** is very clear in these representations.

Hence, the transition experienced by **AU** corresponds to a supramolecular polymerization process without participation (or without detection) of the *c*(**AU**)$_4$ macrocycle. The (**AU**)$_n$-**AU** depolymerization transitions as a function of solvent composition at different overall concentrations (Figure S2B) were analysed by the extended nucleation-elongation model developed by de Greef, Meijer and co-workers.[14] In this equilibrium model, the monomer addition steps in the nucleation regime are described by an equilibrium nucleation constant K_n with a cooperative parameter (σ):

$$\sigma = \frac{K_n}{K_e} < 1 \qquad (2)$$

The elongation equilibrium constant K_e is defined *via*:

$$K_e = e^{\left(\frac{-\Delta G^{0\prime}}{RT}\right)} \qquad (3)$$

Where $\Delta G^{0\prime}$ is the Gibbs free energy gain upon monomer addition. According to denaturation models, the Gibbs free energy ΔG^0 is assumed to be linearly dependent on the volume fraction of added solvent:

$$\Delta G^{0\prime} = \Delta G^0 + m \cdot f \qquad (4)$$

The changes observed by CD for the depolymerization of **GC**^{3} and **AU** (Figure S2A) as a function of the volume fraction of the denaturant (V_{THF}) were fitted by applying a global nonlinear least-squares procedure using this equilibrium model (Figure S2B), which leads to the ΔG^0 and σ values collected in Table S1.

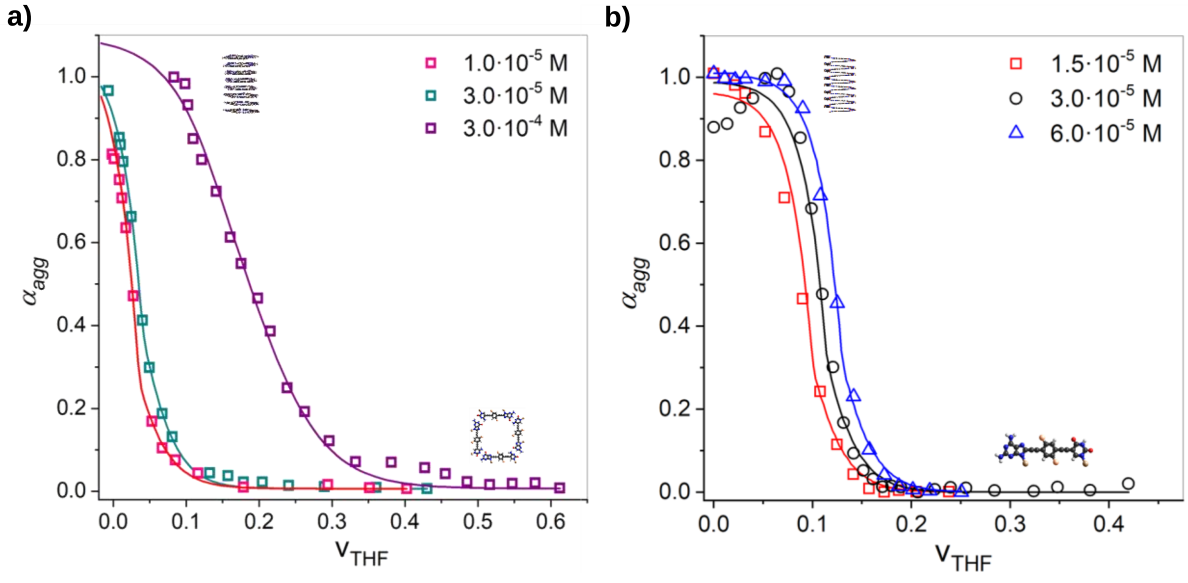

Figure S2B. (**a**) Fitting of the denaturation curves of **GC**^{3} at 429 nm and (**b**) global fitting of the denaturation curves of **AU** at 428 nm to the extended nucleation-elongation model.[14]

Table S1. Thermodynamic parameters obtained from the fitting of the solvent-dependent supramolecular depolymerization of **GC**^{3} and **AU**, depicted in Figure S2B.[14]

---

[14] Korevaar, P. A.; Schaefer, C.; de Greef, T. F. A.; Meijer, E. W. Controlling chemical self-assembly by solvent-dependent dynamics. *J. Am. Chem. Soc*. **2012**, *134*, 13482-13491.

monomer	Conc. (M)	ΔG^0 [a] ($kJmol^{-1}$)	m[b] ($kJmol^{-1}$)	σ[c]	χ^2[d]
**GC**	$3.0\cdot10^{-5}$ M	-34.9 ± 0.5	90.0 ± 9.0	0.19 ± 0.03	$2.1549\cdot10^{-3}$
	$6.0\cdot10^{-5}$ M				
**AU**	$3.0\cdot10^{-5}$ M	-40.1 ± 7.3	117.0 ± 6.7	0.17 ± 0.02	$7.5998\cdot10^{-2}$
	$1.5\cdot10^{-5}$ M				

[a]ΔG^0: Gibbs free energy of the process. [b] m-value: relationship with the volume fraction of denaturant. [c] σ: cooperativity of the process. [d] χ^2: fitting error.

A second difference is revealed at this point: the final absorption, emission and CD spectra of (**GC**)$_n$ and (**AU**)$_n$ polymeric aggregates is considerably different, despite both molecules are almost identical. The only difference between them is the exchange of carbonyl and amino groups at the *C*-6 of purines and *C*-4 of pyrimidines, but all peripheral substituents and chiral groups are the same. As shown in Figure S2C, both molecules experience a red-shift in absorption upon polymerization but the one shown by **AU** is somewhat larger. The disparity is more clearly noted in the CD spectra, where the aggregates (**GC**)$_n$ and (**AU**)$_n$, both coming from compounds having identical *S*-chiral peripheral substituents, display totally different (almost, but not exactly mirror image) CD features. This supports the idea that not only the polymerization mechanism is different for **GC** and **AU**, but the internal structure of the final polymers is different as well.

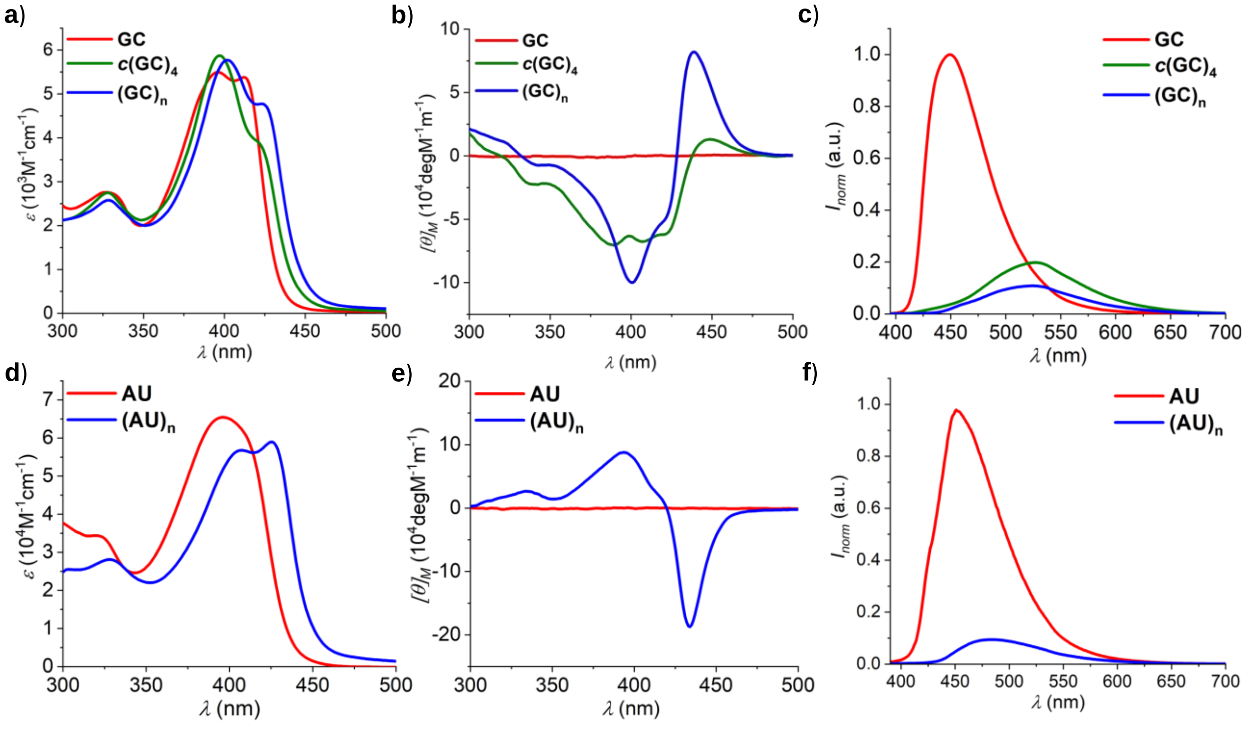

Figure S2C. Absorption (**a,d**), CD (**b,e**) and emission (**c,f**) spectra of monomer and aggregated of **GC** and **AU** species at $3.0\cdot10^{-5}$ M (**GC**: in THF; c(**GC**)$_4$: V_{hep} = 0.70; (**GC**)$_n$: V_{hep} = 0.99; **AU** in THF; (**AU**)$_n$ at V_{hep} = 0.99).

S2.2. Temperature-dependent Experiments

In order to obtain more (quantitative) information about the supramolecular polymerization process of **GC** and **AU**, a set of temperature-dependent experiments were performed in diverse media. We started analyzing THF-heptane mixtures of appropriate composition so that polymers are formed at low temperatures and the monomer is dissociated at high temperatures. This could be achieved in the V_{hep} = 0.90-0.97 range. As reported,[3] compound **GC** at V_{hep} = 0.97 displayed non-sigmoidal CD curves along cooling cycles within the 329-268 K and 10^{-4}-10^{-5} M range (Figure S2D).

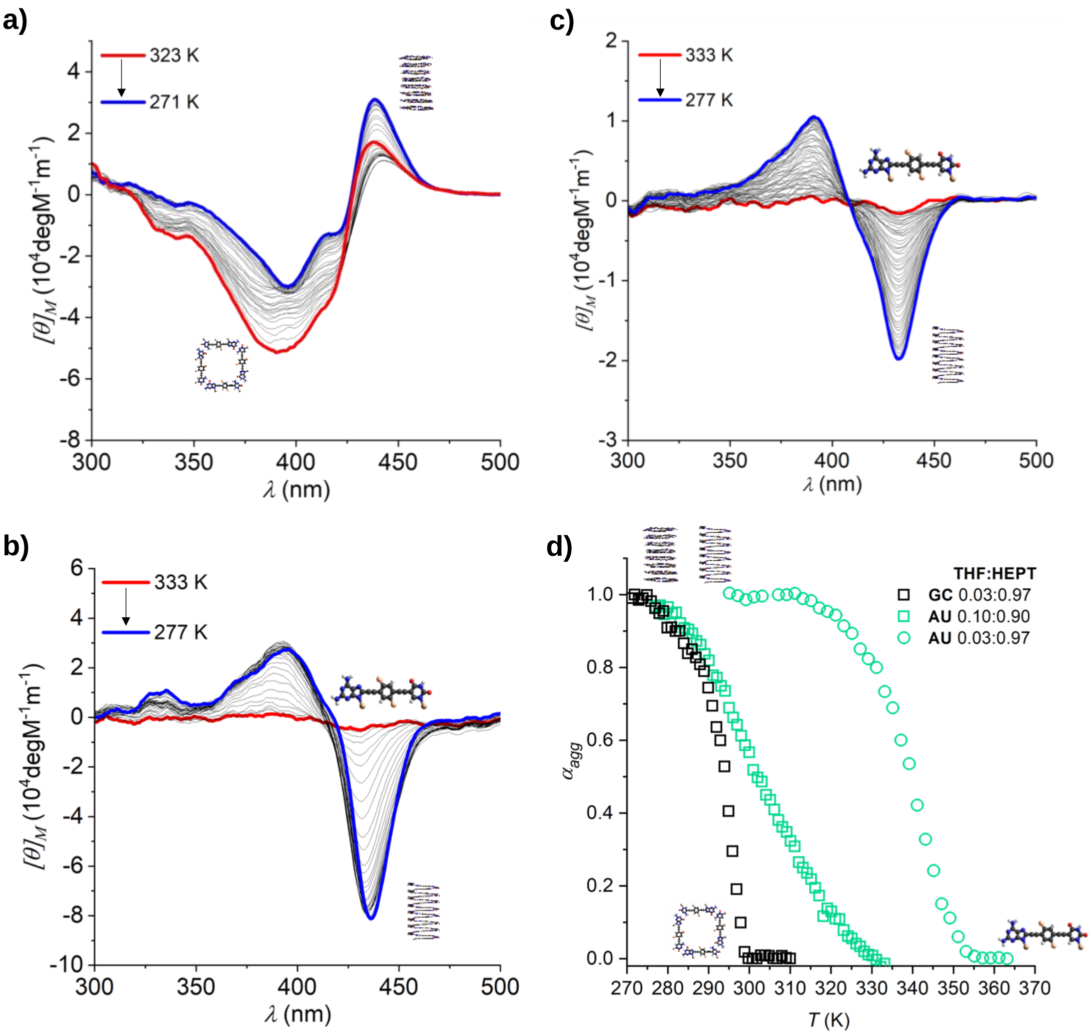

Figure S2D. CD changes experienced by (**a**) **GC**[3] at $3.0\cdot10^{-5}$ M and (**b**) **AU** at $8.0\cdot10^{-6}$ M upon decreasing temperature (1.0 Kmin^{-1}) in a 0.03:0.97 THF-heptane mixture and (**c**) **AU** at $3.0\cdot10^{-5}$ M in a 0.10:0.90 THF-heptane mixtures at $8.0\cdot10^{-6}$ M. (**d**) Normalized CD changes at 429 nm (**GC**) and 430 nm (**AU**) as a function of the temperature for **GC** and **AU** (α_{agg} = fraction of aggregated nanotubes).

In contrast, in the same solvent mixture at V_{hep} = 0.97 the (**AU**)$_n$ nanotubes displayed, in line with other experiments, much higher stability than the (**GC**)$_n$ nanotubes, and could not be depolymerized even at the highest temperatures at the same $3.0\cdot10^{-5}$ M concentration, as shown in Figure S2E. Only through the highly sensitive emission technique a tiny fraction of monomers could be detected at high temperatures in this mixture rich in heptane (Figure S2Ec). However, decreasing the concentration to $8.0\cdot10^{-6}$ M, just above the detection limit of our CD equipment for this sample, allowed us to record the whole polymerization

process in cooling experiments at V_{hep} = 0.97 (Figure S2Db), from the **AU** monomer at high temperatures to the (**AU**)$_n$ nanotubes at low temperatures (see the trend displayed in Figure S2Dd).

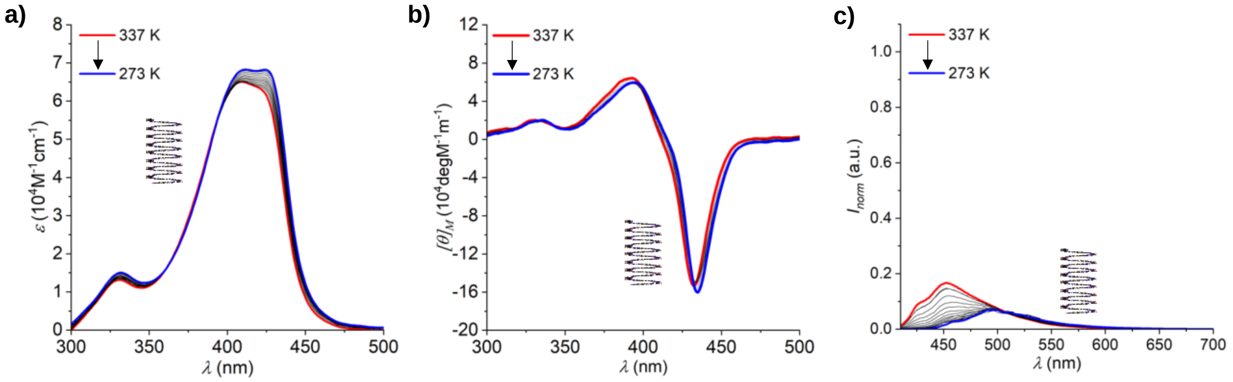

Figure S2E. (**a**) UV-vis, (**b**) CD and (**c**) emission changes experienced by **AU** upon decreasing temperature in a 0.03:0.97 THF-heptane mixture at a $3.0 \cdot 10^{-5}$ M.

Similar experiments performed for **AU** at V_{hep} = 0.90, where aggregation is slightly weaker, in order to monitor as much of the aggregation process as possible, displayed curves that look sigmoidal and are more consistent with a polymerization process of much lower cooperativity (Figure S2F). Obviously, the higher the concentration, the higher the degree of aggregation attained at low temperatures. In addition, heating and cooling curves virtually overlapped each other, which is consistent with a thermodynamically controlled process without lag phases.

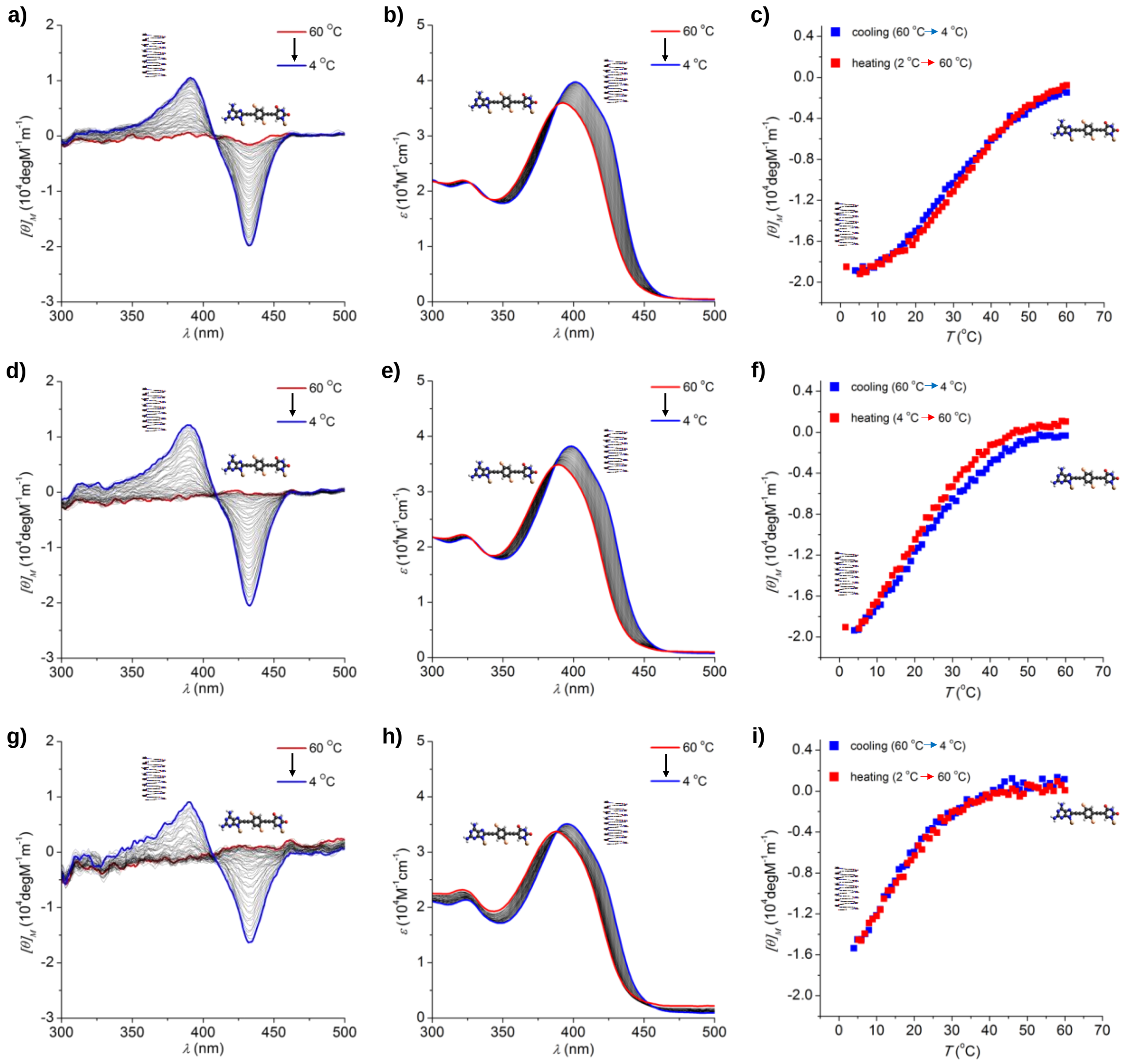

Figure S2F. (**a**,**d, g**) CD and (**b**,**e**,**h**) UV-vis changes experienced by **AU** upon decreasing temperature in a 0.10:0.90 THF-heptane mixture at a constant concentration of (**a,b**) $3.0 \cdot 10^{-5}$ M, (**d,e**) $2.0 \cdot 10^{-5}$ M and (**g,h**) $1.0 \cdot 10^{-5}$ M. Cooling and heating curves at 430 nm from the CD measurements: (**c**) $3.0 \cdot 10^{-5}$ M, (**f**) $2.0 \cdot 10^{-5}$ M and (**i**) $1.0 \cdot 10^{-5}$ M.

We then turned our attention to temperature-dependent experiments performed in a single apolar solvent, since the ^{1}H NMR experiments had already revealed the appearance of very broad features at low temperatures for 10^{-3} M solutions of **AU** in $CDCl_3$ and toluene-D_8 (see Figures S1A and S1C). In a first approach, **GC** and **AU** solutions in THF, $CHCl_3$ and toluene at relatively high concentration (just above 10^{-4} M; to compare with the ^{1}H NMR results) were measured. In these conditions, we described in our previous work[3] that **GC** formed cyclic tetramers quantitatively ($CHCl_3$ and toluene) or in equilibrium with the monomer (THF), and these cyclic species are characterized by distinct CD and well-defined ^{1}H NMR spectra (see Figures S1A and S1C). On the contrary, **AU** revealed monomer-like UV-vis spectra in THF or $CHCl_3$ and no CD Cotton effect was found even when the samples were cooled down to 0 ºC. However, in the less polar toluene solvent, a CD effect appeared with a zero crossing at 412 nm, which was very similar to the one found in THF:heptane mixtures of high V_{hep}. Such CD signal disappeared at high temperatures and increased in intensity at low temperatures. The aggregation was also clearly noted by UV-vis spectroscopy

in the form of a considerable red-shift and the appearance of a red-shifted shoulder. As a matter of fact, the aggregation state could be monitored by the naked eye: the monomer solution was virtually colorless, while the cold aggregated solutions presented a vivid yellow colour. It is notable that this vivid color change was not observed for the **GC** polymers.

Therefore, temperature-dependent experiments were next performed in toluene. For **GC**,[3] a monomer-cyclic tetramer equilibrium was recorded as a function of temperature in this solvent (Figure S2G). For **AU**, however, the same spectroscopic features attributed previously to the (**AU**)$_n$ polymers were detected in toluene at different concentrations above 10^{-4} M, indicating again the presence of a single supramolecular polymerization process (Figure S2H). Thus, this aromatic non-polar solvent provided probably the best scenario that differentiates **GC** and **AU** self-assembly, the former being associated in very stable discrete cyclic *c*(**GC**)$_4$ species, and the latter yielding supramolecular helical (**AU**)$_n$ polymers.

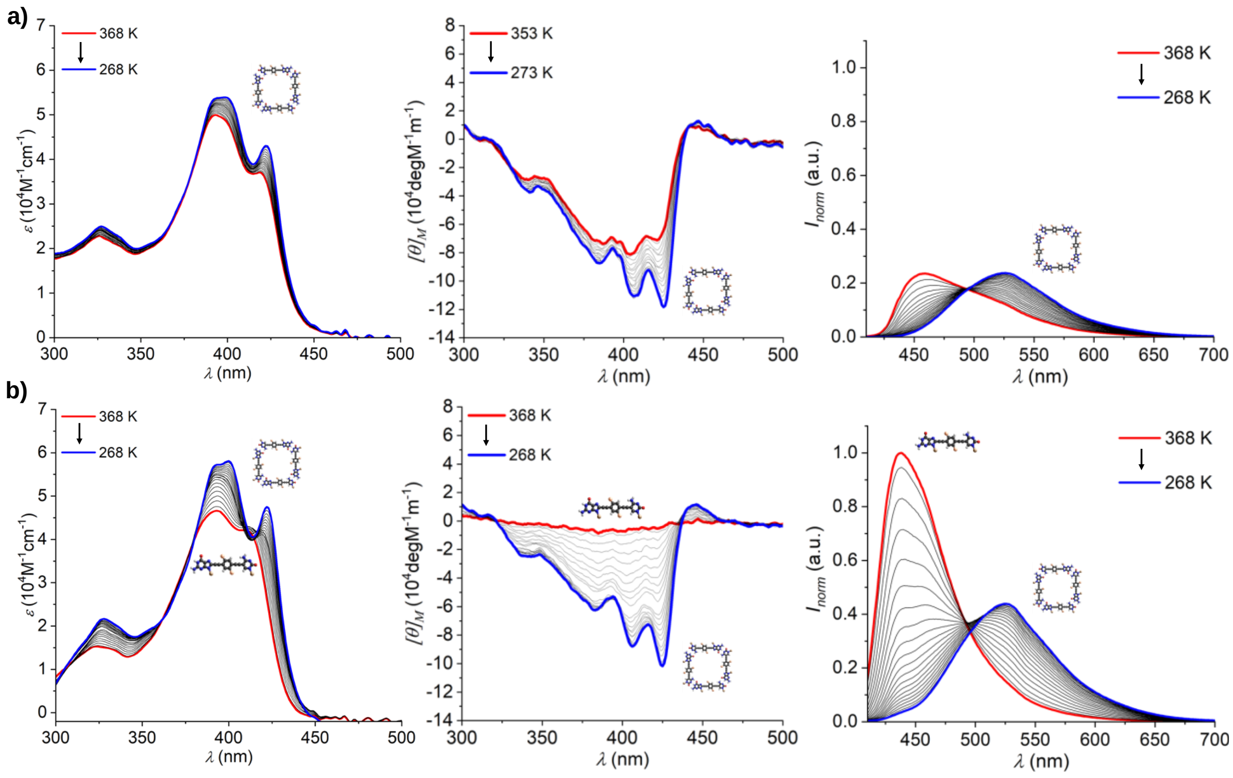

Figure S2G. UV-vis, CD and fluorescence changes (from left to right) observed in temperature-dependent experiments of **GC** in toluene at (**a**) 1.0·10^{-4} M and (**b**) 1.0·10^{-5} M.[3]

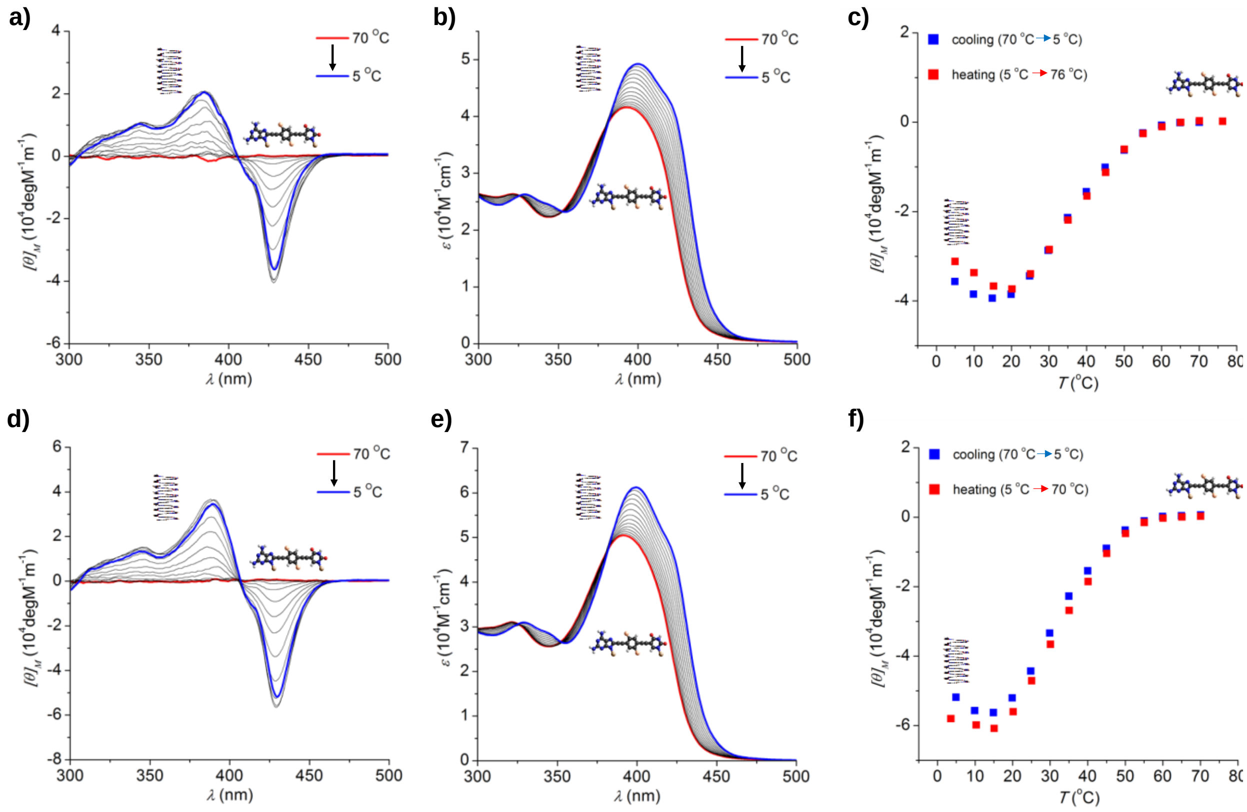

Figure S2H. (**a**,**d**) CD and UV-vis (**b**,**e**) changes experienced by **AU** upon decreasing temperature in toluene at (**a**,**b**) $6.0{\cdot}10^{-4}$ M and (**d**,**e**) $3.0{\cdot}10^{-4}$ M. Cooling and heating curves from the CD measurements at 430 nm: (**c**) $6.0{\cdot}10^{-4}$ M, (**f**) $3.0{\cdot}10^{-4}$ M.

The cooling curves obtained from the temperature-dependent experiments of **AU** in toluene as well as in THF-heptane mixtures (Figures S2F and S2H) were fitted to a cooperative nucleation–elongation model developed by Meijer and co-workers (Figure S2I).[15,16] According to this model, the polymerization process can be divided in a nucleation and an elongation phase. The magnitudes T_e (elongation temperature), K_n and K_e (nucleation and elongation constants), σ (cooperativity factor), $\Delta H°_n$ and $\Delta H°_e$ (nucleation and elongation enthalpies), and $\Delta S°$ (polymerization entropy) can be obtained from a non-linear least-square analysis of the experimental melting curves using the following equations:

$$K_n = e^{\left(\frac{-(\Delta H_e^0 - \Delta H_n^o) - T\Delta S^o}{RT}\right)} \qquad (5)$$

$$K_e = e^{\left(\frac{-(\Delta H_e^0 - T\Delta S^o)}{RT}\right)} \qquad (6)$$

$$\sigma = \frac{K_n}{K_e} = e^{\left(\frac{\Delta H_n^o}{RT}\right)} \qquad (7)$$

The thermodynamic parameters for the nucleation and for the elongation process of the supramolecular polymerization of **AU** are shown in Table S2.

---

[15] Maarkvort, A. J.; Ten Eikelder, H. M. M.; Hilbers, P. J. J.; De Greef, T. F. A.; Meijer, E. W. Theoretical models of nonlinear effects in two-component cooperative supramolecular copolymerizations. *Nat. Commun.* **2011**, *2*, 509.

[16] Ten Eikelder, H. M. M.; Markwoort, A. J.; De Greef, T. F. A.; Hilbers, P. A. J. An Equilibrium Model for Chiral Amplification in Supramolecular Polymers. *J. Phys. Chem. B* **2012**, *116*, 5291-5301.

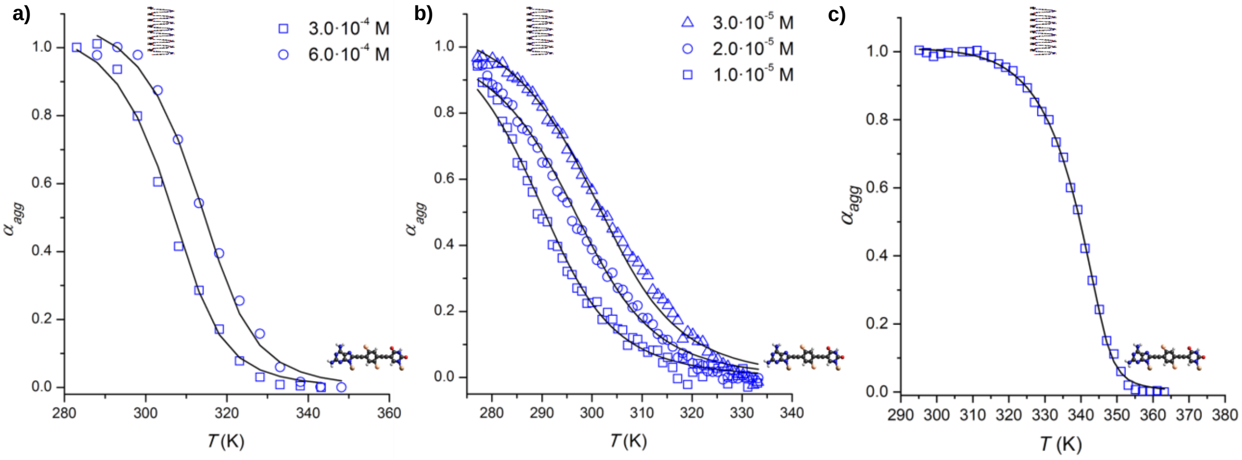

Figure S2I. Global fitting of the cooling curves of **AU** at 430 nm in (**a**) toluene, (**b**) 0.10:0.90 and (**c**) 0.03:0.97 in THF-heptane mixture to the nucleation-elongation model.[15,16]

Table S2. Thermodynamic parameters calculated from the global fitting of the cooling curves to the nucleation-elongation model of: **AU** at different concentrations in toluene and in a 0.10:0.90 THF:heptane mixtures, **AU** at 8.0·10^{-6} M in a 0.03:0.97 THF:heptane mixture and **GC3**[3] in a 0.03:0.97 THF:heptane mixture (Figure S2I).[15,16]

solvent	*Conc.* (M)	T_e [a] (*K*)	K_n [b] (M^{-1})	K_e [c] (M^{-1})	σ [d]	ΔH^0 [e] (*kJmol*$^{-1}$)	ΔS^0 [f] (*Jmol*$^{-1}$*K*$^{-1}$)	ΔH_n^0 [g] (*kJmol*$^{-1}$)
**AU** Toluene	6.0·10^{-4}	316.7 ± 1.9	279.7	2543.2	0.11	-81.2 ± 9.2	-195.7 ± 3.0	-5.7 ± 1.4
	3.0·10^{-4}	309.7 ± 1.2						
**AU** THF:hept 0.10:0.90	3.0·10^{-5}	302.6 ± 0.8	188.7	725.7	0.26	-70.8 ± 3.0	-147.4 ± 1.0	-4.0 ± 0.4
	2.0·10^{-5}	298.3 ± 0.7						
	1.0·10^{-5}	291.3 ± 0.5						
**AU** THF:hept 0.03:0.97	8.0·10^{-6}	345.4 ± 0.4	1811.2	1.8·10^{5}	0.01	-97.7 ± 3.5	-185.3 ± 0.1	-12.1 ± 0.8
**GC** THF:hept 0.03:0.97	2.0·10^{-5} [g]	295.7 ± 0.2	4.02·10^{1}	1.34·10^{5}	3.0·10^{-4}	-116.6 ± 3.0	-0.29 ± 0.01	-20.1 ± 3.0
	3.0·10^{-5} [g]	298.3 ± 0.2						

[a] T_e, elongation temperature. [b] K_n, nucleation constant. [c] K_e, elongation constant. [d] σ, cooperativity factor. [e] ΔH^0, elongation enthalpy [f] ΔS^0, entropy. [g] ΔH^0_n, nucleation enthalpy, [g] Since the tetramer is considered as the "supramonomer", the total concentration was divided by 4.[3]

A final difference between **GC** and **AU** is now clear after these calculations: <u>the polymerization transition of **AU** is significantly less cooperative than that of **GC**</u> (or, more correctly, *c*(**GC**)$_4$). For instance, the cooperative parameter for the supramolecular polymerization of **GC** was calculated as σ = 3.0·10^{-4} in THF-heptane 3:97, whereas those for the polymerization of **AU** were calculated as σ = 2.6·10^{-1} in THF-heptane 10:90, σ = 1.0·10^{-2} in THF-heptane 3:97 and σ = 1.1·10^{-1} in toluene. In all these solvent- or temperature-dependent experiments we made sure that we worked under equilibrium conditions, by applying slow rates

(1.0 K·min^{-1}) and that the observed deviations are not caused by time or solvent effects. The final spectroscopic features did not evolve with time or thermal annealing, and cooling and heating curves converting the monomer into the aggregate, and *vice versa*, perfectly overlapped at various concentrations, as shown previously in Figures S2F and S2H.

S2.3. Concentration-dependent Experiments

Finally, as an additional tool to characterize the supramolecular polymerization process and a last attempt to detect the formation of intermediate *c*(**AU**)$_4$ macrocycles, dilution experiments monitored by CD, UV-vis and emission spectroscopy in toluene were carried out (Figure S2J). At the highest 5.0·10^{-4} M concentration, the supramolecular polymer was formed, though not quantitatively, as could be clearly observed from the CD intensity and the shape of the emission band, which suggested that free monomer remained in solution. As the concentration is reduced, depolymerization takes place, which is best observed by the disappearance of the CD signals. For all the experiments the dissociation of the supramolecular polymers resulted in the characteristic monomer features and, again, no intermediate species could be detected.

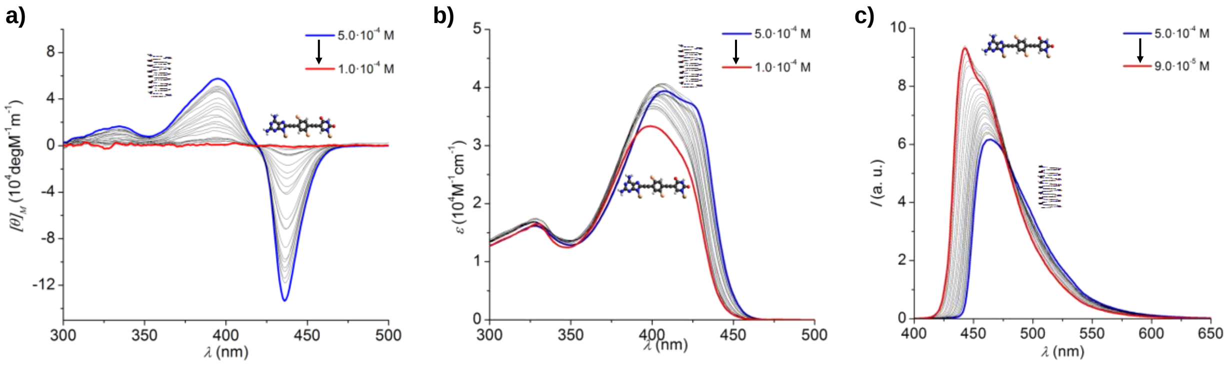

Figure S2J. Concentration-dependent (**a**) CD, (**b**) UV-vis and (**c**) emission experiments of **AU** in toluene at 298 K.

S2.4. Circularly Polarized Luminescence (CPL) Experiments

Figures S2K-L show the circularly polarized luminescence (CPL) and the linearly polarized luminescence (LPL) spectra of **GC** and **AU** in diverse solvents. For the (**GC**)$_n$ and (**AU**)$_n$ polymers obtained in THF:heptane solutions at V_{hep} = 0.99 or in toluene (only for **AU**), the CPL band is in correspondence with the high energy side of the fluorescent feature and the CPL sign is the same as the sign for the lowest energy CD band. The corresponding fluorescence band recorded on the same apparatus was normalized to 1 and consequently the *Δ*I value is related to the g_{lum} factor. To be more precise, due to the fact that the maximum in the CPL spectrum does not coincide with the fluorescence maximum for (**AU**)$_n$, the g_{lum} value at 450 nm is -1.1x10^{-3}, whereas for (**GC**)$_n$ at 500 nm is 1.6x10^{-3} in the chosen solution. We also checked that we do not have appreciable contributions from LPL, as documented in Figures S2K-La.

On the other hand, the fluorescence CPL spectra of the **GC** cyclic tetramer (*c*(**GC**)$_4$) was measured in THF:heptane solutions at V_{hep} = 0.40 and shown in Figure S2K for comparison.

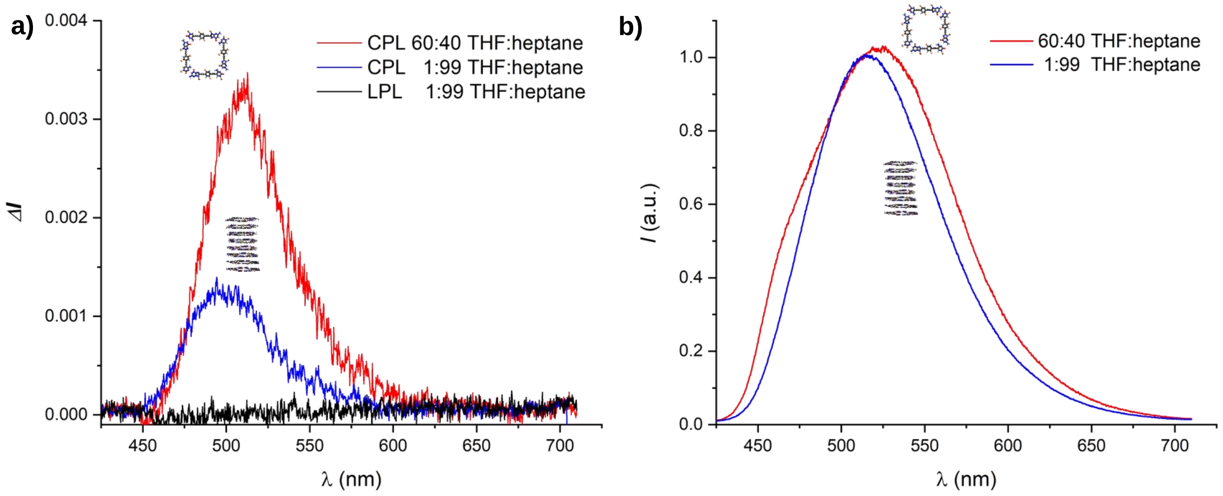

Figure S2K. (**a**) CPL and (**b**) fluorescence spectra of **GC** at 3.0·10^{-5} M in THF:heptane mixtures at V_{hep} = 0.40 and V_{hep} = 0.99 (λ_{exc} = 420 nm in both cases). For comparison purposes the LPL spectrum is also reported in (**a**).

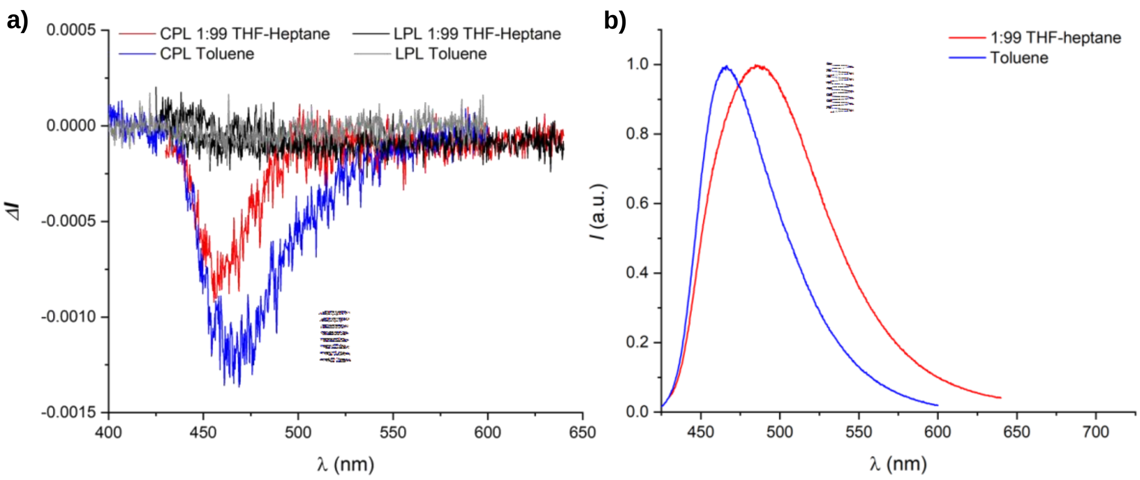

Figure S2L. (**a**) CPL and (**b**) fluorescence spectra of **AU** at 3.0·10^{-5} M in THF:heptane at V_{hep} = 0.99 (λ_{exc} = 420 nm) and in toluene (λ_{exc} = 400 nm). For comparison purposes the LPL spectra are also reported in (**a**).

S3. Microscopy Characterization of the Self-assembled Nanotubes

SEM measurements (Figure S3A,B) confirmed the formation of large unidimensional aggregates, consisting of nanotube bundles as revealed by TEM (see below). Solutions of **GC** or **AU** in a 0.10:0.90 THF-heptane solvent mixture were drop-casted onto a glass substrate. The samples were then metallized with copper and measured in a Philips XL30 S-FEG Scanning Electron Microscope.

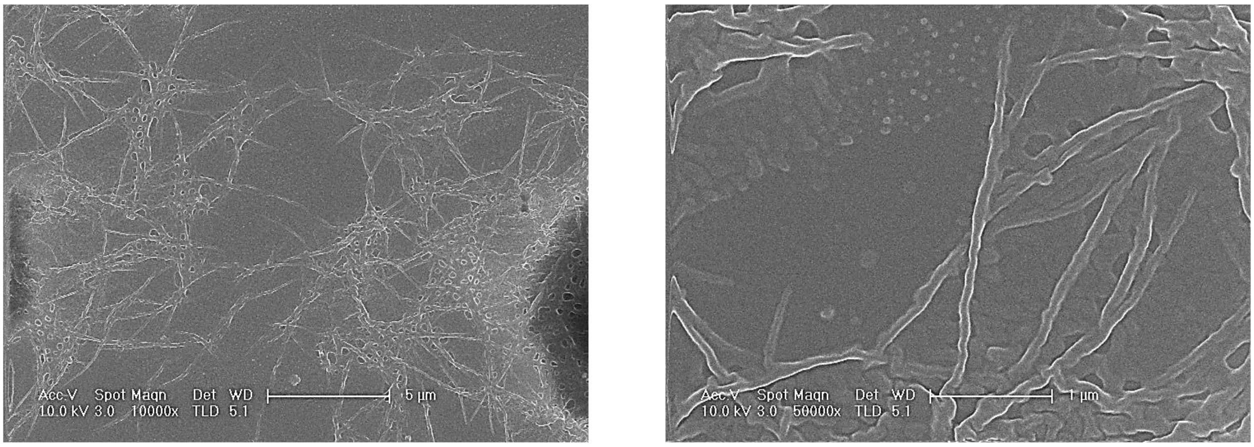

Figure S3A. SEM images at different scales of **GC** drop-cast from a $1.0{\cdot}10^{-4}$ M 0.10:0.90 THF-heptane solution.

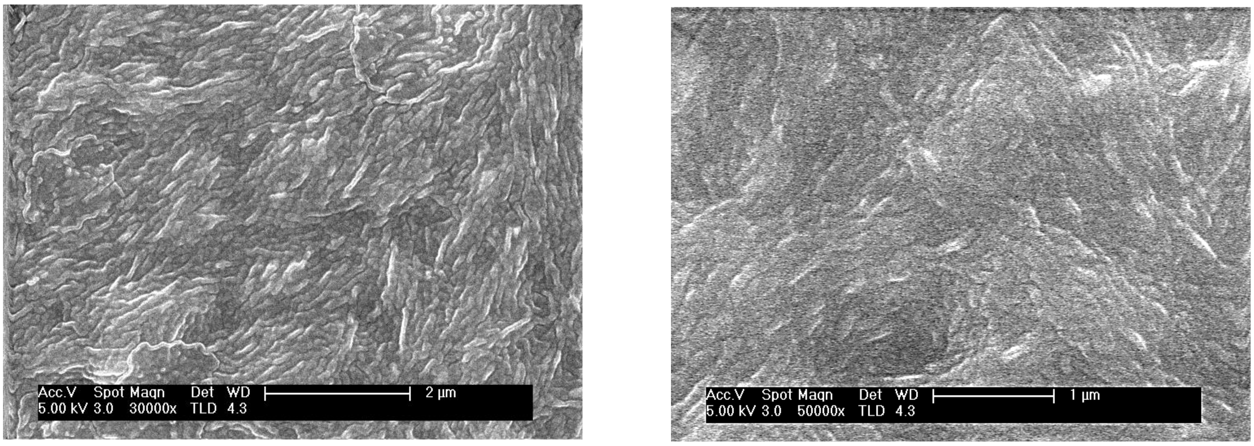

Figure S3B. SEM images at different scales of **AU** drop-cast from a $3.0{\cdot}10^{-4}$ M 0.10:0.90 THF-heptane solution.

TEM measurements were performed with a JEOL JEM 1400 PLUS equipment working at an accelerating voltage of 40 to 120 kV, which provide higher contrast and resolution (Figures S3C,D). Solutions of **GC** or **AU** in THF-heptane mixtures at high heptane content were drop-casted into 200-mesh formvar copper grids coated with carbon. TEM analysis revealed that **GC** aggregates consist of heavily bundled longitudinal objects with a measured diameter of 3.9 ± 0.7 nm, which coincides with the hard aromatic section of the cyclic tetramers (Figures S3C).[3] **AU** is able to form almost identical self-assembled nanotubes with a similar mean diameter of 4.3 ± 0.3 nm (Figures S3D).

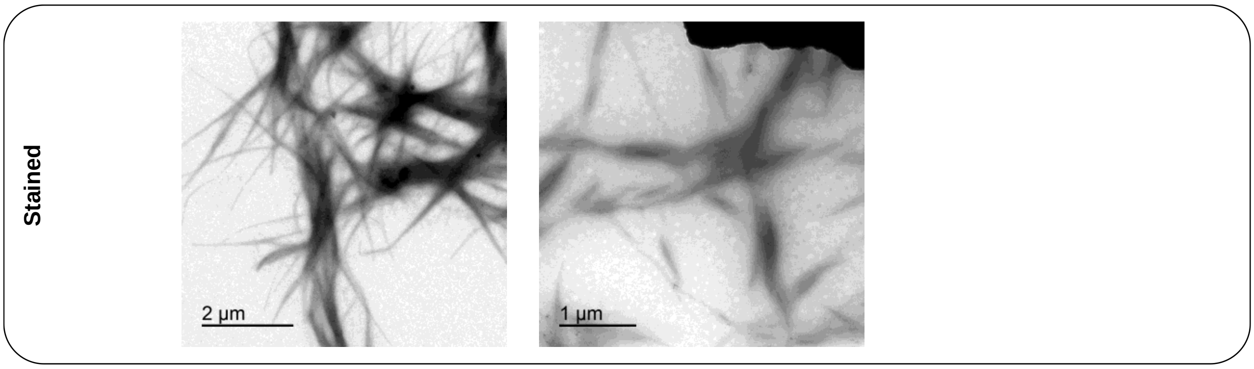

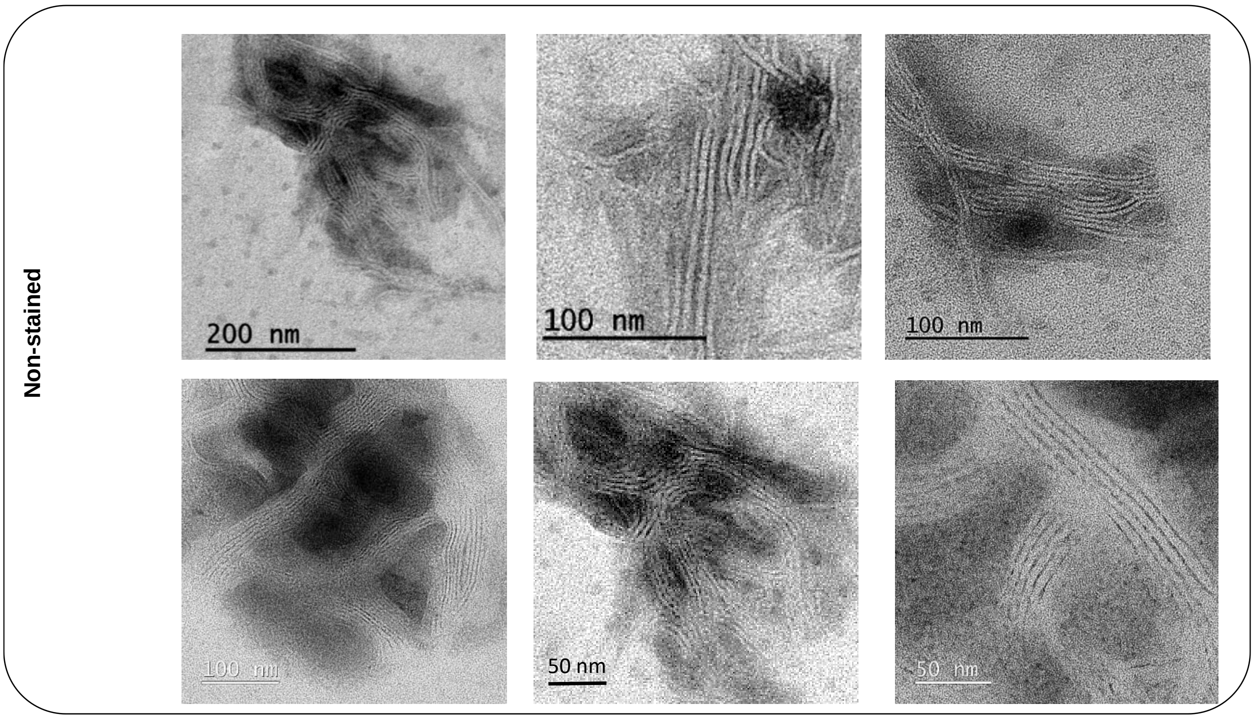

Figure S3C. TEM images at different scales from solutions of **GC** in $CHCl_3$/MCH 0.1:0.9 (stained with a 1% solution of uranyl acetate in water; top panel) or in $1.0{\cdot}10^{-5}$ M solutions in 0.01:0.99 THF-heptane (non-stained; bottom panel).

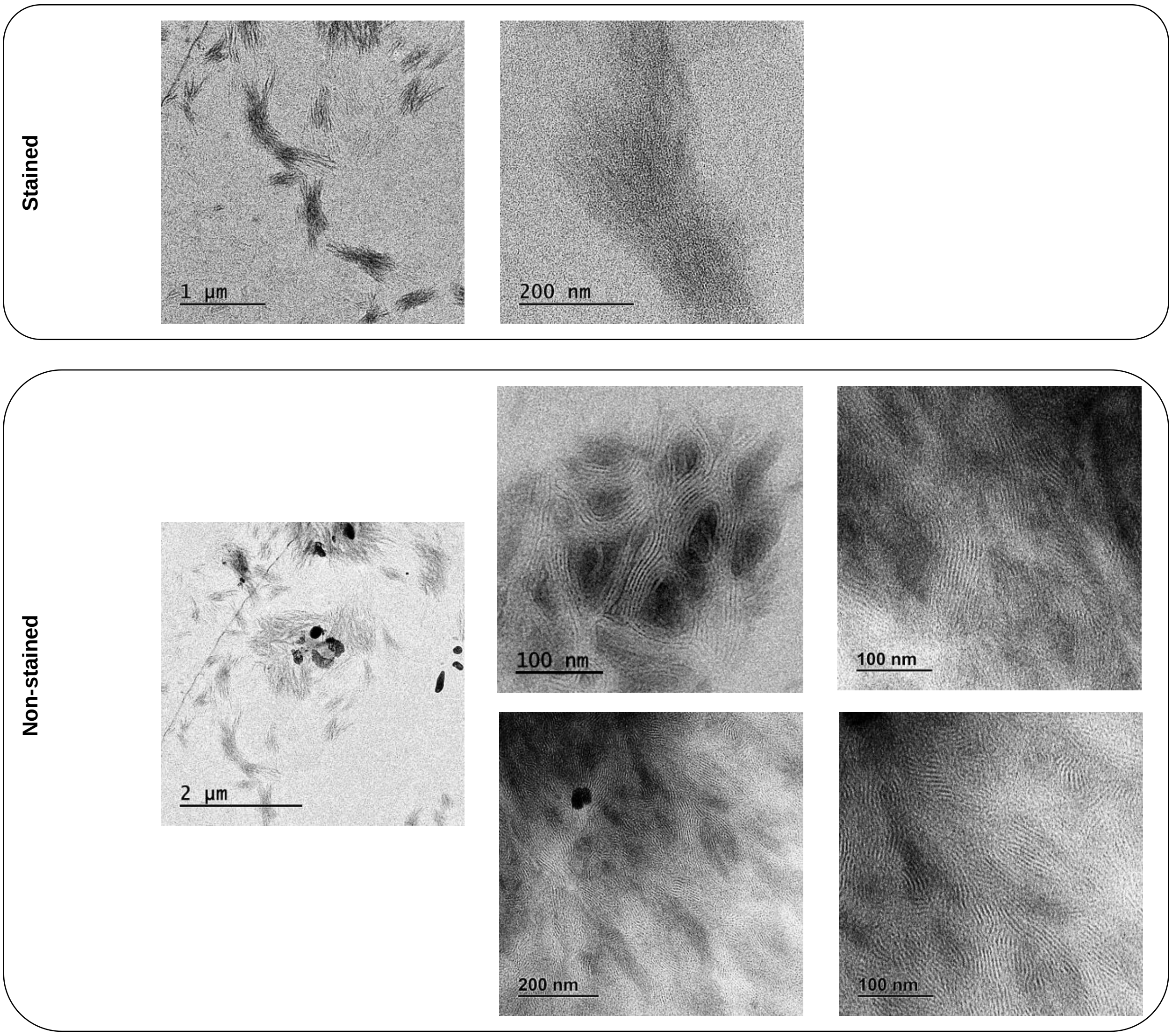

Figure S3D. TEM images at different scales from solutions of **AU** in 0.08:0.92 THF-heptane mixtures at $3.0\cdot10^{-5}$ M (stained with a 1% solution of uranyl acetate in water; top panel), or at $1.0\cdot10^{-5}$ M (non-stained; bottom panel).

S4. Self-sorting Experiments

Playing with relative **GC**/**AU** monomer concentration and solvent composition, we were able to find conditions in which we could: 1) scan the whole aggregation landscape and record consecutively **GC** cyclotetramerization, **AU** polymerization and *c*(**GC**)$_4$ polymerization; and 2) monitor **AU** polymerization in the presence of *c*(**GC**)$_4$ macrocycles.

The first case was realized by increasing V_{cy}/V_{hep} in THF: cyclohexane-D$_{12}$/heptane mixtures, and could be respectively monitored by ^{1}H NMR (Figure S4A) and CD (Figure S4B) spectroscopy due to the distinct signatures of *c*(**GC**)$_4$, (**GC**)$_n$ and (**AU**)$_n$. In the case of ^{1}H NMR measurements recorded at [**GC**] = [**AU**] = 2.0·10^{-4} M, our starting situation at V_{cy} = 0 and 2.0·10^{-3} M concentration in each compound (top spectrum) reveals a mixture of dissociated **AU** and **GC** monomers (2% DMSO-D$_6$ needed to be added to fully dissociate **GC**). Then, as V_{cy} was increased, aggregation was triggered fro both compounds, as shown schematically at the right of Figure S4A. We could monitor first **GC** cyclotetramerization in the presence of short **AU** open oligomers at V_{cy} < 0.25. Then, between 0.3 < V_{cy} < 0.7, the *c*(**GC**)$_4$ species is formed quantitatively while the **AU** oligomers keep growing, as evidenced by the downfield shift experienced by the H-bonded U-imide proton. At V_{cy} > 0.75, the signals of the **AU** oligomers disappear due to polymerization. Finally, the signals of the *c*(**GC**)$_4$ macrocycle broaden and disappear as well above V_{cy} > 0.85. For the CD experiments, a screening of multiple concentrations and [**GC**]/[**AU**] ratios allowed us to choose [**GC**] = [**AU**] = 1.5·10^{-4} M as the best conditions to record consecutively all self-assembly processes. In Figure S4Ba-c, the evolution of the CD spectra as V_{hep} is increased is shown for **GC**, **AU** and their 1:1 mixture. The trends recorded at a fixed wavelength from these set of spectra, as well as those obtained from the arithmetic sum of **GC** and **AU** spectra, are shown in Figure S4Bd. The cyclotetramerization process of **GC** is first monitored within the V_{hep} = 0-0.3 range. Then, the polymerization of each compound is monitored successively at higher V_{hep} values: **AU** polymerizes first at V_{hep} > 0.8, while (**GC**)$_n$ polymerization is activated just after V_{hep} > 0.9. Finally, the comparison of the CD spectra of **GC**, **AU**, their 1:1 mixture, and the arithmetic sum of **GC**+**AU** spectra, taken from 3 selected V_{hep} values, is shown in Figure S4Be.

In order to discard chiral self-sorting events, the same solvent-dependent experiments shown in Figure S4B by mixing *S*-**GC** and *S*-**AU** were now performed by mixing *R*-**GC**[3] and *S*-**AU** molecules under the same conditions. The results, shown in Figure S4C, reveal the same strong narcissistic self-sorting process with almost identical transitions for the heterochiral and the homochiral mixtures.

Secondly, we could explore several conditions in which **AU** polymerization can be recorded as a function of the temperature in the presence of the *c*(**GC**)$_4$ macrocycle. As shown in Figure S4D employing this time fluorescence emission changes, in a THF:heptane mixture set at V_{hep} = 0.9, the *c*(**GC**)$_4$ species at a 1.0·10^{-5} M concentration is sufficiently stable and does not dissociate or polymerize with temperature changes in the -10 – 80 ºC range. On the contrary, the whole polymerization process could be recorded for **AU** at a 3.0·10^{-5} M concentration. The same situation was observed in toluene at slightly higher concentrations (2.0·10^{-4} M), as shown in Figure S4E using the spectral changes monitored by fluorescence emission or CD. The **AU** polymerization trends are once again virtually the same in the presence or absence of *c*(**GC**)$_4$ macrocycles, which supports the notion that these two dinucleobase monomers do not mix in their assemblies.

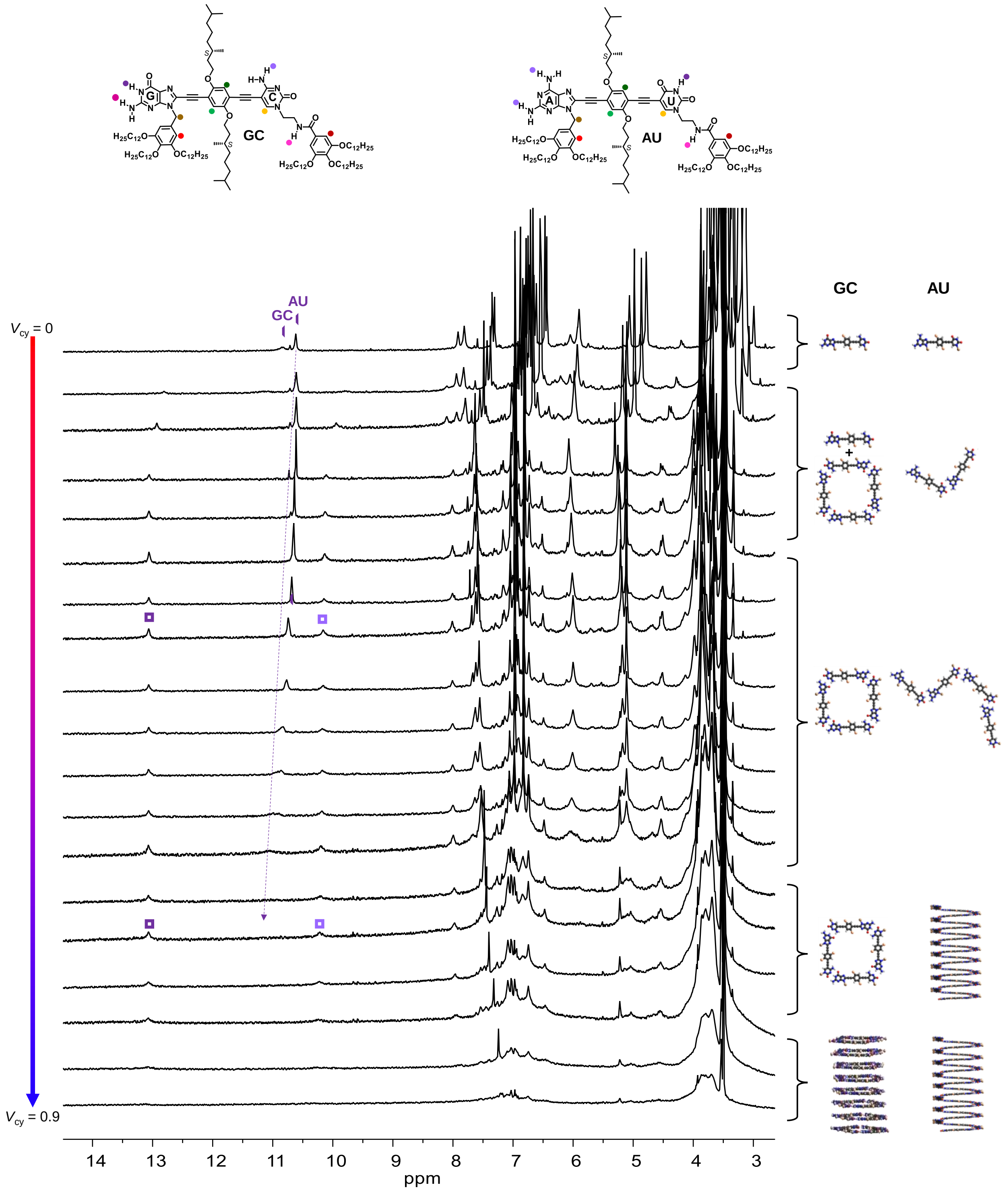

Figure S4A. Self-assembly of a 1:1 mixture of **GC** + **AU** by progressively increasing the volume fraction of cyclohexane-D_{12} (V_{cy}) in mixtures with THF-D_8 monitored by ^1H NMR. Rod-shaped marks correspond to monomeric or linear oligomeric species, while square-shaped marks correspond to cyclic tetramers. In all cases: [**GC**] = [**AU**] = $2.0 \cdot 10^{-3}$ M; T = 298 K. The pictures at the right indicate approximately the distribution of supramolecular **GC** and **AU** species as V_{cy} is increased. Please compare with Figure S1B, where the individual evolution of **GC** and **AU** is displayed.

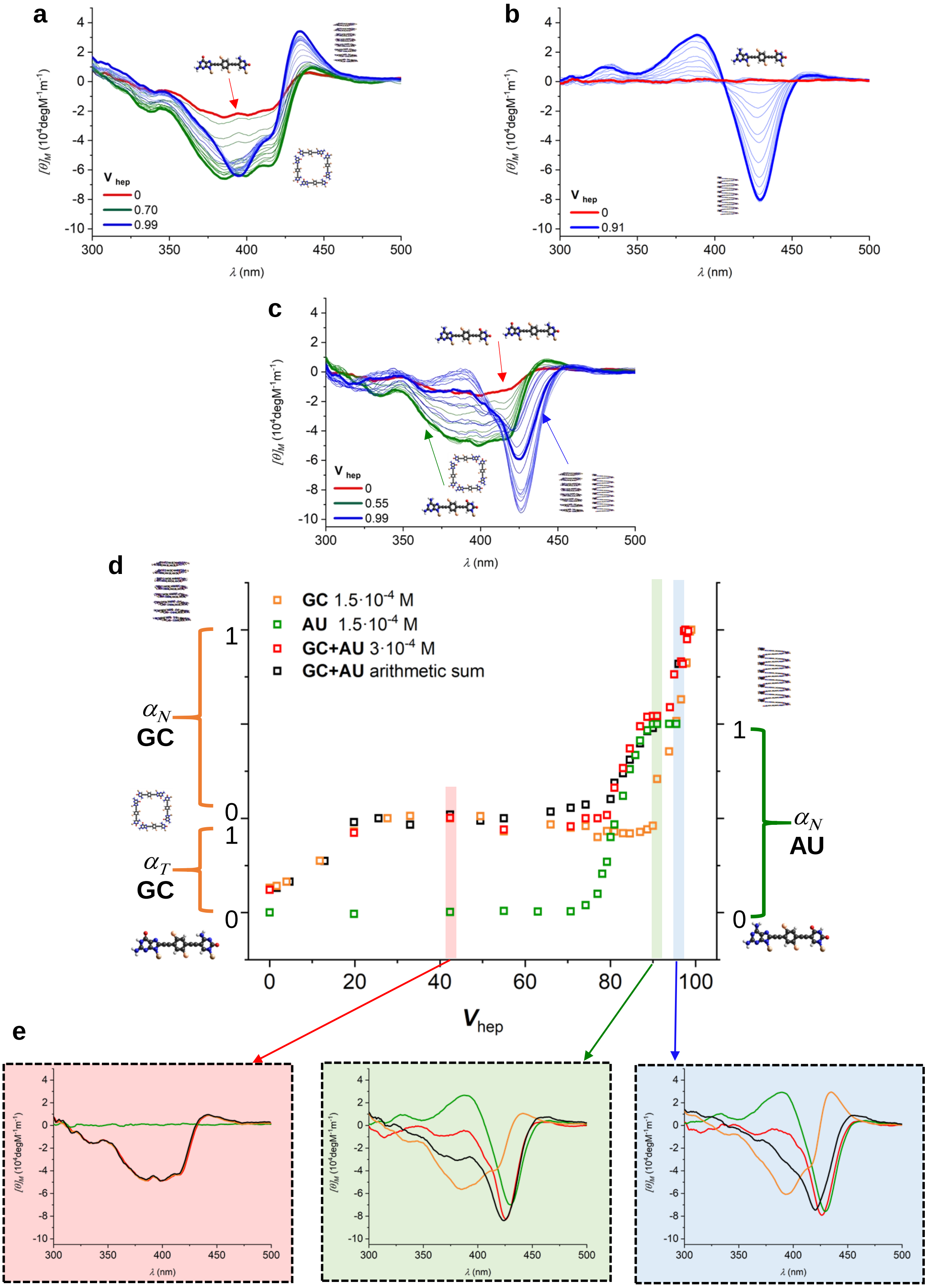

Figure S4B. Self-assembly of (**a**) *S*-**GC**, (**b**) *S*-**AU** and (**c**) their 1:1 mixture monitored by CD spectroscopy by progressively increasing the volume fraction of heptane (V_{hep}) in mixtures with THF. (**d**) Normalized CD changes at 435 nm as a function of V_{hep} for **GC**, **AU**, their mixture, and the arithmetic sum of **GC**+**AU** CD intensity taken from the isolated samples (α_T = fraction of cyclotetramers, α_N = fraction of nanotubes). (**e**) Comparison of the CD spectra of **GC**, **AU**, their mixture, and the arithmetic sum of **GC**+**AU** spectra taken from the isolated samples at 3 selected V_{hep} values. In all cases: [**GC**] = [**AU**] = 1.5·10^{-4} M; *T* = 298 K.

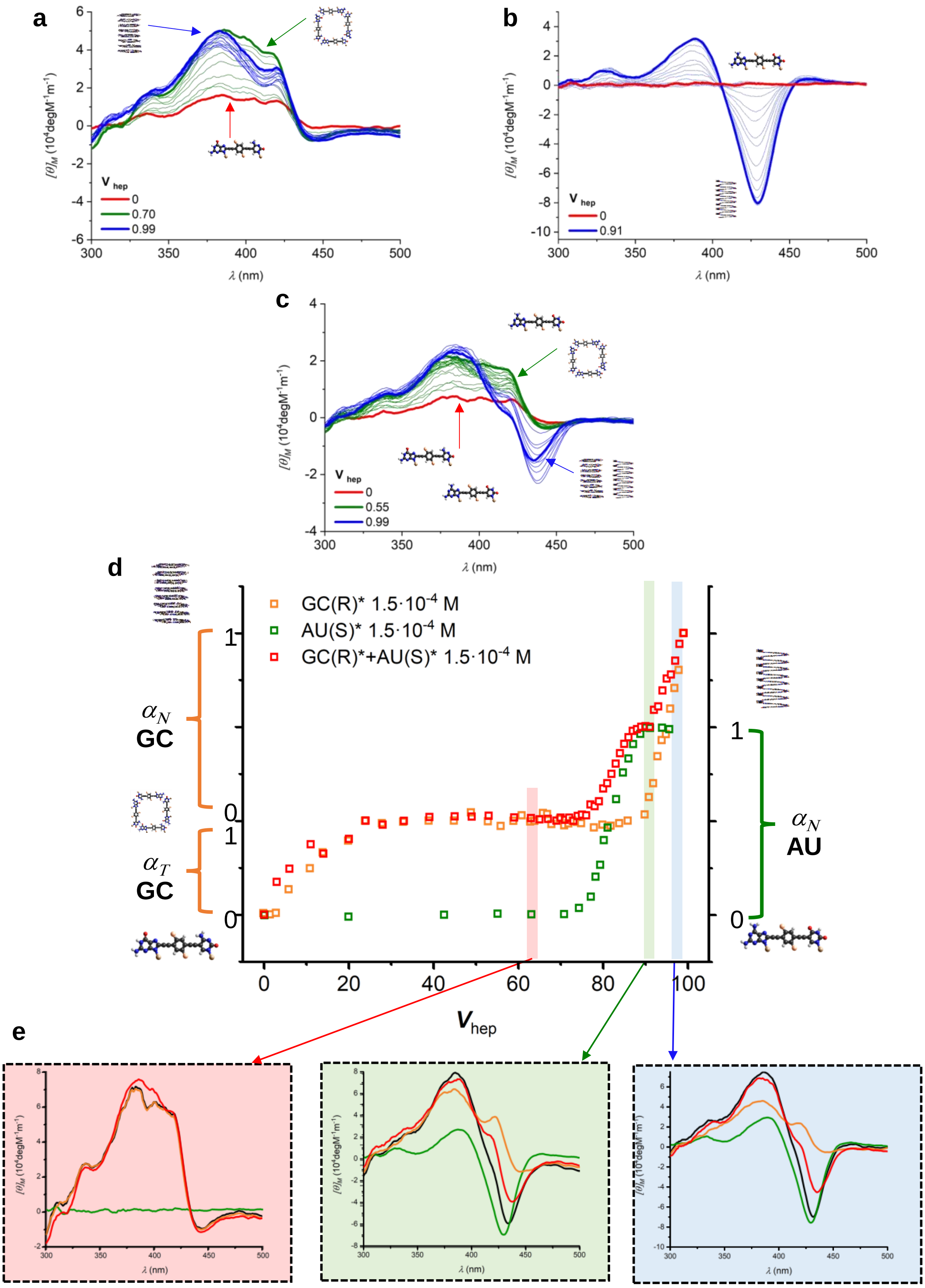

Figure S4C. Self-assembly of (**a**) *R*-**GC**, (**b**) *S*- **AU** and (**c**) their 1:1 mixture monitored by CD spectroscopy by progressively increasing the volume fraction of heptane (V_{hep}) in mixtures with THF. (**d**) Normalized CD changes at 435 nm as a function of V_{hep} for **GC**, **AU**, their mixture, and the arithmetic sum of **GC**+**AU** CD intensity taken from the isolated samples (α_T = fraction of cyclotetramers, α_N = fraction of nanotubes). (**e**) Comparison of the CD spectra of **GC**, **AU**, their mixture, and the arithmetic sum of **GC**+**AU** spectra taken from the isolated samples at 3 selected V_{hep} values. In all cases: [**GC**] = [**AU**] = 1.5·10^{-4} M; *T* = 298 K.

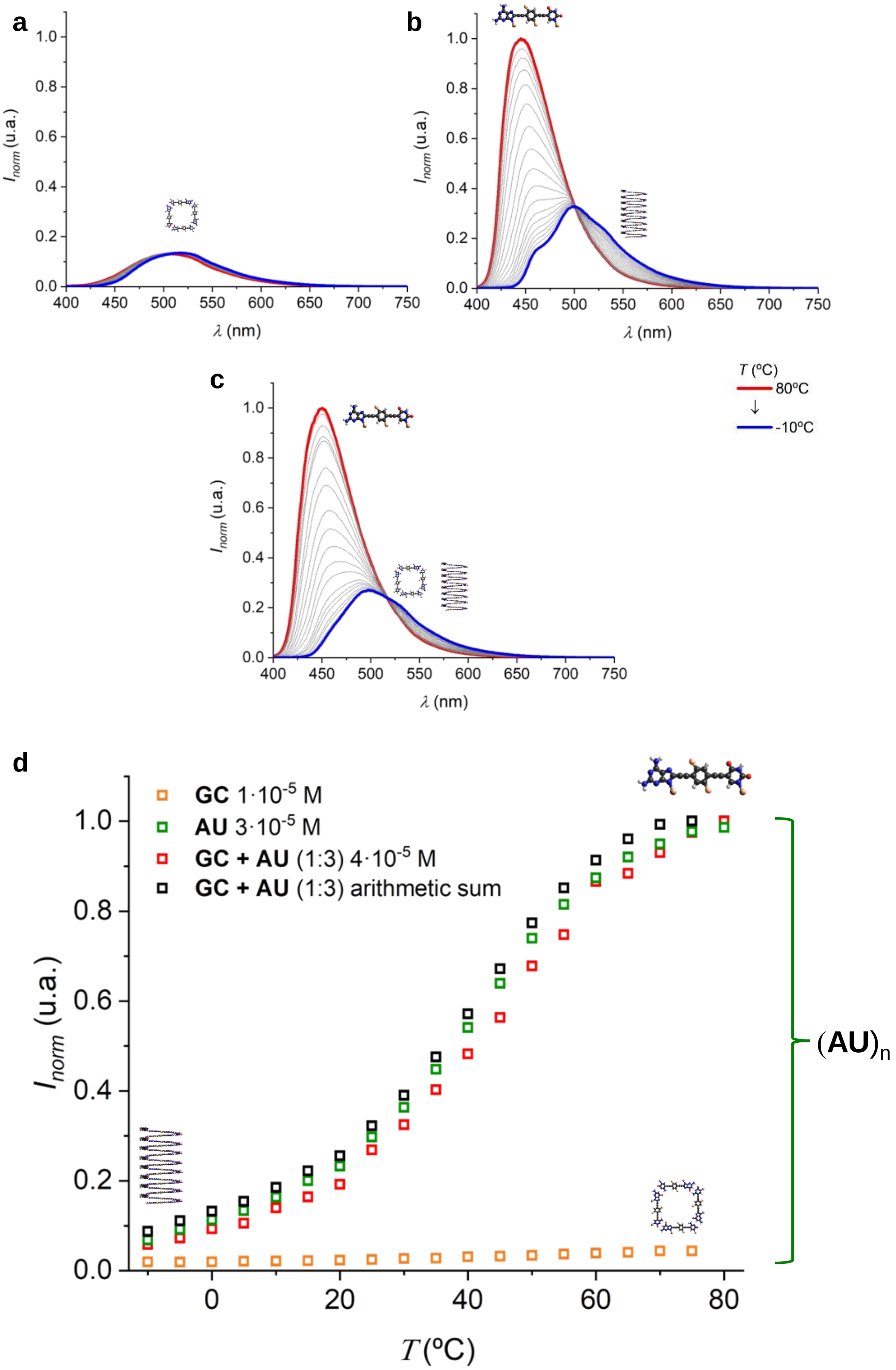

Figure S4D. Self-assembly of (**a**) **GC**, (**b**) **AU** and (**c**) their 1:3 mixture monitored by emission spectroscopy by progressively decreasing temperature in a THF:heptane mixture at V_{hep} = 0.9. (**d**) Normalized emission changes at 450 nm as a function of *T* for **GC**, **AU**, their mixture, and the arithmetic sum of **GC**+**AU** emission intensity taken from the isolated samples. In all cases: [**GC**] = 1.0·10^{-5} M and [**AU**] = 3.0·10^{-5} M.

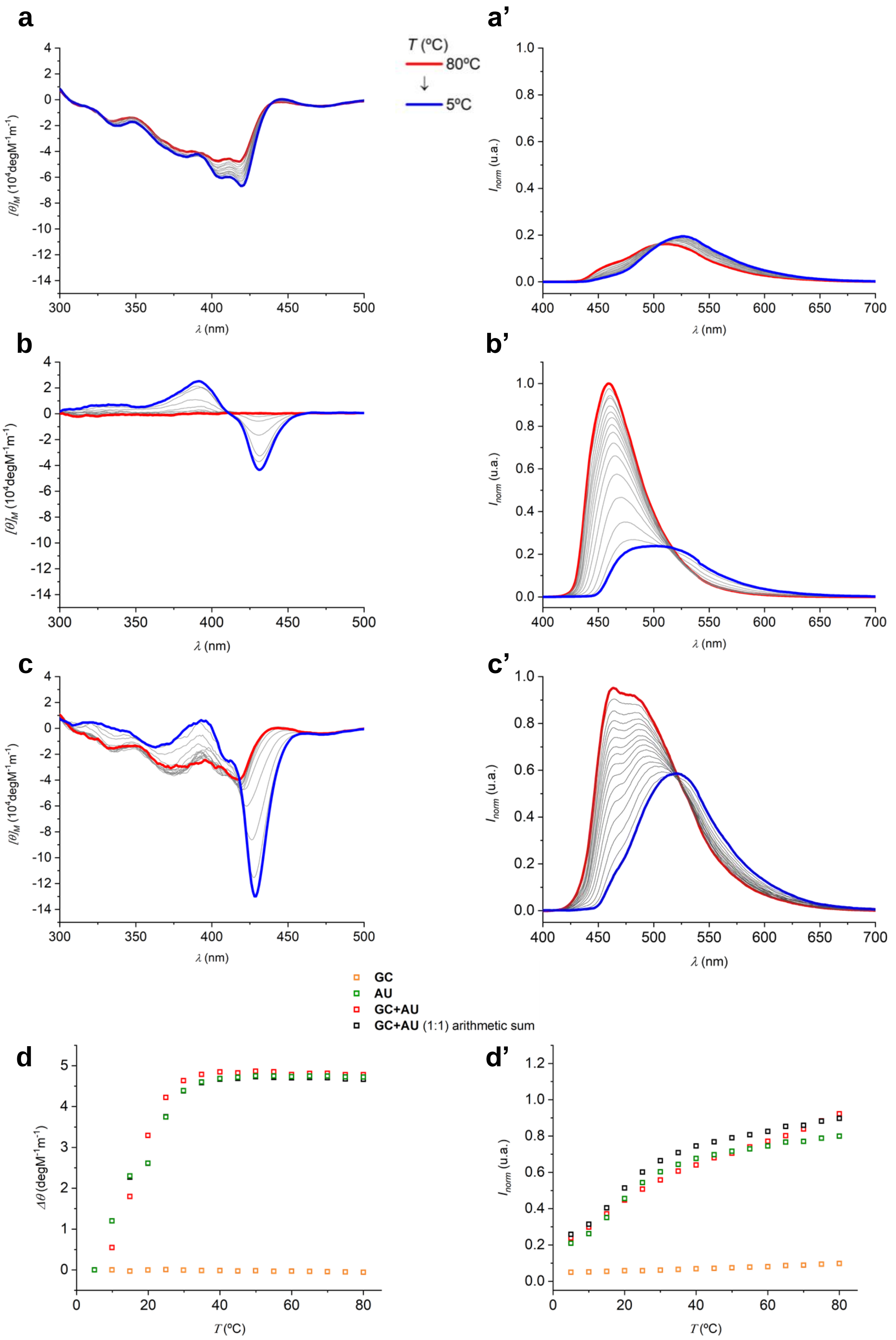

Figure S4E. Self-assembly of (**a**,**a'**) **GC**, (**b**,**b'**) **AU** and (**c**,**c'**) their 1:1 mixture monitored by (**a**-**d**) CD or (**a'**-**d'**) emission spectroscopy by progressively decreasing temperature in toluene. Normalized (**d**) CD changes at 440 nm or (**d'**) emission changes at 475 nm as a function of *T* for **GC**, **AU**, their mixture, and the arithmetic sum of **GC**+**AU** CD/emission intensity taken from the isolated samples. In all cases [**GC**] = [**AU**] = $2.0\cdot10^{-4}$ M.

S5. Theoretical Calculations

S5.1. Parameterization of GC and AU models

For molecular dynamics (MD) simulations of **GC**/**AU** (Figure S5A) a combination of AMBER14SB[17] and GAFF force fields[18] was used. The geometry of each molecule of interest was optimized on HF/6-31G* level of theory in Gaussian 16.[19] Then, the restrained electrostatic potential (RESP) partial charges[20] were obtained for all atoms in the molecule by antechamber from the AMBER package.[21] For nucleic bases, AMBER14SB atom types were used as in RNA residues in the force field database, for the rest of the atoms GAFF atom types were used. Bonds, angles and dihedrals were used from AMBER14SB for the nucleic bases, rest of the parameters were taken from GAFF force field as assigned by tleap.

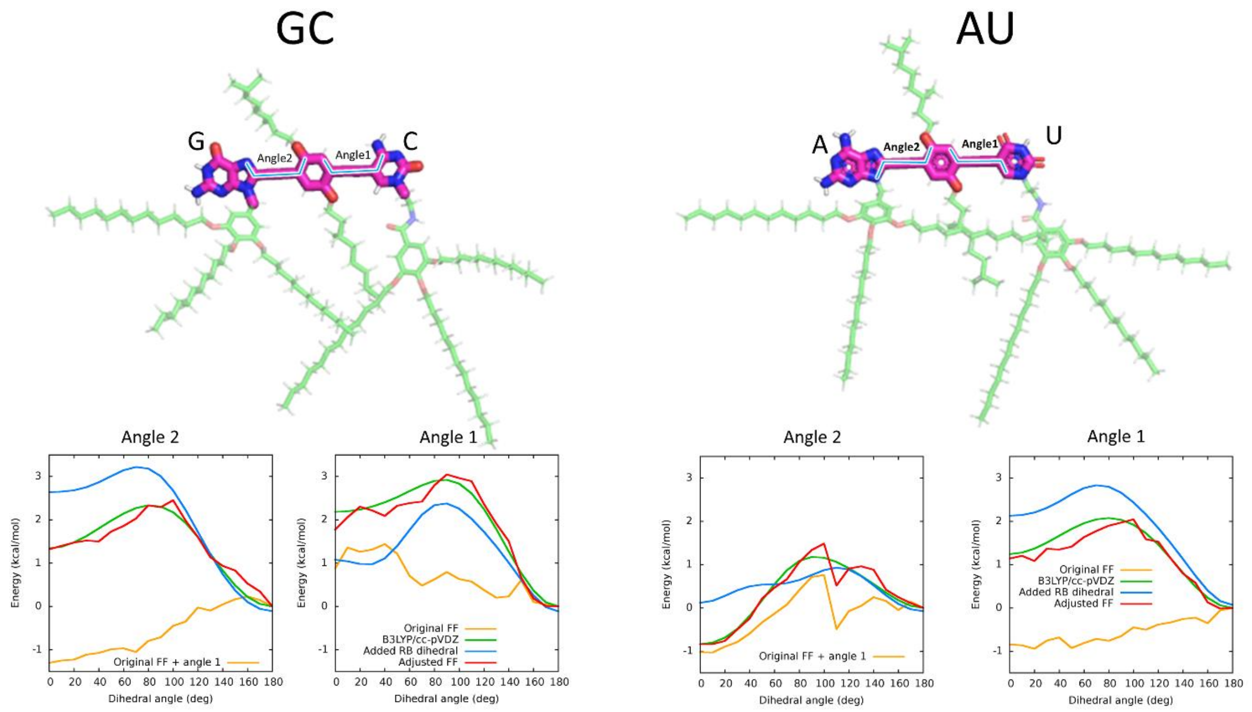

Figure S5A. Molecular structures (upper panel) with two highlighted dihedral angles that were monitored in PES, where angle 1 corresponds to dihedral near C and U and angle 2 corresponds to G and A. As shown in later figures – the magenta region is called 'core' in this text. Lower panel shows potential energy scans over two dihedral angles for each molecule – QM PES is depicted in green and adjusted FF PES is shown in red.

---

[17] Maier, J. A.; Martinez, C.; Kasavajhala, K.; Wickstrom, L.; Hauser, K. E.; Simmerling, C. ff14SB: Improving the Accuracy of Protein Side Chain and Backbone Parameters from ff99SB *J. Chem. Theory Comput.* **2015**, *11*, 3696-3713.

[18] Wang, J.; Wolf, R. M.; Caldwell, J. W.; Kollman, P. A.; Case, D. A. Development and testing of a general amber force field *J. Comput. Chem.* **2004**, *25*, 1157-1174.

[19] Frisch, M. J.; Trucks, G. W.; Schlegel, H. B.; Scuseria, G. E.; Robb, M. A.; Cheeseman, J. R.; Scalmani, G.; Barone, V.; Petersson, G. A.; Nakatsuji, H.; Li, X.; Caricato, M.; Marenich, A. V.; Bloino, J.; Janesko, B. G.; Gomperts, R.; Mennucci, B.; Hratchian, H. P.; Ortiz, J. V.; Izmaylov, A. F.; Sonnenberg, J. L.; Williams; Ding, F.; Lipparini, F.; Egidi, F.; Goings, J.; Peng, B.; Petrone, A.; Henderson, T.; Ranasinghe, D.; Zakrzewski, V. G.; Gao, J.; Rega, N.; Zheng, G.; Liang, W.; Hada, M.; Ehara, M.; Toyota, K.; Fukuda, R.; Hasegawa, J.; Ishida, M.; Nakajima, T.; Honda, Y.; Kitao, O.; Nakai, H.; Vreven, T.; Throssell, K.; Montgomery Jr., J. A.; Peralta, J. E.; Ogliaro, F.; Bearpark, M. J.; Heyd, J. J.; Brothers, E. N.; Kudin, K. N.; Staroverov, V. N.; Keith, T. A.; Kobayashi, R.; Normand, J.; Raghavachari, K.; Rendell, A. P.; Burant, J. C.; Iyengar, S. S.; Tomasi, J.; Cossi, M.; Millam, J. M.; Klene, M.; Adamo, C.; Cammi, R.; Ochterski, J. W.; Martin, R. L.; Morokuma, K.; Farkas, O.; Foresman, J. B.; Fox, D. J. Gaussian 16 Rev. C.01, Wallingford, CT, **2016**.

[20] Wang, J.; Cieplak, P.; Kollman, P. A. How well does a restrained electrostatic potential (RESP) model perform in calculating conformational energies of organic and biological molecules? *J. Comput. Chem.* **2000**, *21*, 1049-1074.

[21] Case, D.; Betz, R.; Cerutti, D. S.; Cheatham, T.; Darden, T.; Duke, R.; Giese, T. J.; Gohlke, H.; Götz, A.; Homeyer, N.; Izadi, S.; Janowski, P.; Kaus, J.; Kovalenko, A.; Lee, T.-S.; LeGrand, S.; Li, P.; Lin, C.; Luchko, T.; Kollman, P. *Amber 2016, University of California, San Francisco*, **2016**, 10.13140/RG.2.2.27958.70729.

In order to reproduce well the geometry of the molecules, two potential energy scans (PES) on B3LYP/cc-pVDZ level of theory[22] were performed for each molecule, rotating the nucleobase with respect to a central ring (see Figure S5A) by steps of 15°. On the MM level, a relaxed PES was performed for each of the molecules on one of the dihedral angles (by constraining the dihedral angle with steps of 10° and by energy minimization of the rest of the molecule). Then, the difference between the QM PES and MM PES was calculated and fitted to the Ryckaert-Bellemans potential. Afterwards, the same procedure was performed for the other angle (including the 'angle 1' parameters). For a check, final MM PES was calculated with all final parameters (Figure S5A, Table S3).

Table S3. Ryckaert-Belleman's dihedral parameters (kJ/mol).

	C0	C1	C2	C3	C4	C5
**GC**_angle1	9.94828	-1.10234	-11.47970	5.44371	3.53154	-6.83304
**GC**_angle2	12.57258	-6.14936	-10.35275	0.92771	3.06416	-0.53366
**AU_**angle1	11.13320	-4.47443	-5.71527	3.23559	-0.81595	-3.07483
**AU**_angle2	3.17066	2.98375	0.08098	-7.25853	-3.16430	3.87187

S5.2. MD simulations

S5.2.1. Preferential conformation of single monomer molecules

We adopted a bottom-up approach and started by performing a study of the conformational preferences of the monomers. We performed all MD simulations in GROMACS 5.1.4.[23] In order to investigate the conformational preferences of the **AU** and **GC** molecules, we performed unbiased simulations of the rod-like cores by replacing all long side chains by methyl groups in order to eliminate the effect of interactions between the tails. We solvated the cores in 1 % THF and 99 % heptane and simulated them for 50 ns. We defined the planarity as an angle between nucleobase planes and monitored the distribution of a dihedral angle defining those (Figure 5, Figure S5B).

[22] Jr., T. H. D. Gaussian basis sets for use in correlated molecular calculations. I. The atoms boron through neon and hydrogen *J. Chem. Phys.* **1989**, *90*, 1007-1023.
[23] Van Der Spoel, D.; Lindahl, E.; Hess, B.; Groenhof, G.; Mark, A. E.; Berendsen, H. J. C. GROMACS: Fast, flexible, and free *J. Comput. Chem.* **2005**, *26*, 1701-1718.

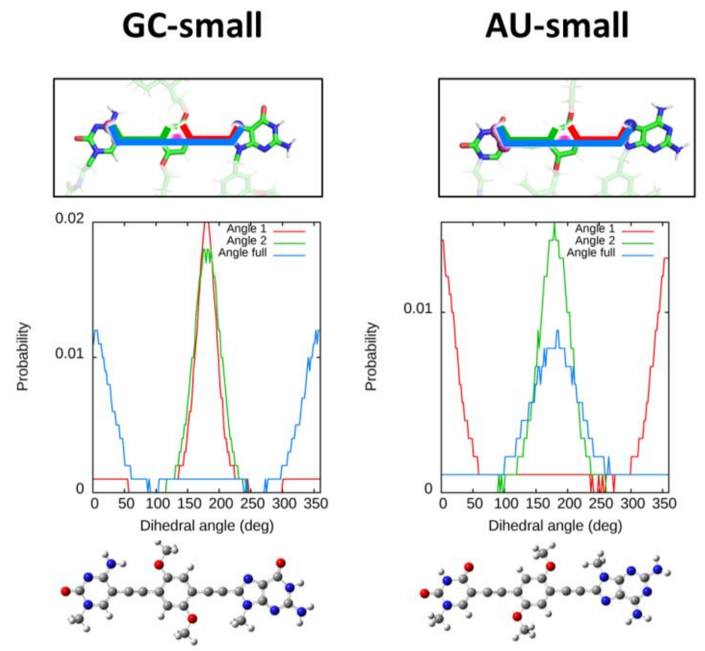

Figure S5B. Distribution of angle 1 and angle 2 and a 'full' angle (colors correspond to the dihedrals sketched in the upper panel) during MD simulation of a single molecule of **GC** and **AU** cores (methyls instead of alkyl tails). In the lower panel the most populated conformation is displayed – **GC** prefers side chains in the same orientation while **AU** prefers opposite orientation.

Our analysis showed that both molecules preferentially adopted planar conformations, but that the orientations of the side chains were different. While side chains at the nucleobases preferred to remain on the same side in the **GC** system (the Watson-Crick edges adopt a *syn* disposition), the opposite was true for the **AU** system (the Watson-Crick edges tend to adopt an *anti* relative arrangement), as made clear in the respective distributions of the dihedral angle in Figure S5B. This energetic difference is already in favor of the higher propensity shown by the **GC** molecule, when compared to **AU**, to assemble in cyclic tetramer systems, which demand a *syn* arrangement of the Watson-Crick pairs.

S5.2.2. MD simulations of layers of squares

For building of a nanotube started from smaller blocks, we prepared squares (in *xy* plane) from individual molecules manually and then we built structures for two (2SQ) and eight squares (8SQ) by multiplying and shifting of the initial structure by 0.5 nm per layer in *z*-direction. The structures were solvated by a mixture of 1 % THF and 99 % of heptane. We performed energy minimization of the system with position restraints applied on the molecular cores, followed by two 100 ps equilibration simulation with 1 fs time step (first one with applied position restraints, second one with the whole system free) and then we performed a production run with 2 fs time step, Parrinello-Rahman barostat[24] keeping 1 bar in isotropic conditions, Nose-Hoover thermostat[25] for 310 K. We simulated 2SQ for 10 ns and 8SQ for 50+ ns.

[24] Parrinello, M.; Rahman, A. Polymorphic transitions in single crystals: A new molecular dynamics method *J. Appl. Phys.* **1981**, *52*, 7182-7190.
[25] Stoyanov, S. D.; Groot, R. D. From molecular dynamics to hydrodynamics: A novel Galilean invariant thermostat *J. Chem. Phys.* **2005**, *122*, 114112.

- **Two Squares – 8 molecules**

Three simulations were performed for each system with two squares (2SQ) on top of each other (during the adjustment of the dihedral parameters etc.). In one simulation, **AU** ended up in a helix, in all other cases both systems stayed in two separate squares (Figure S5C).

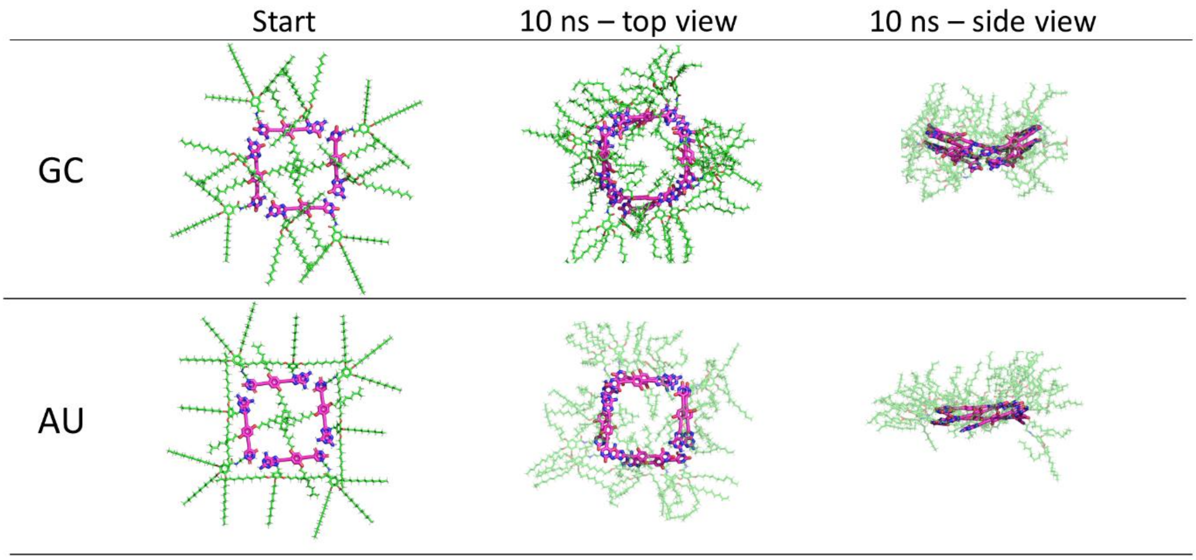

Figure S5C. Initial and final conformations of **GC** and **AU**. Molecular cores are depicted as magenta sticks, side chains as semi-transparent green sticks (colour corresponds to carbon atoms). Oxygen is shown in red, nitrogen in blue and hydrogen in white.

- **8 squares – 32 molecules**

With 32 molecules (*i.e.*, eight squares (8SQ) on top of each other), the differences between the systems are clearer. **AU** created a helix. Starting with eight layers (squares) we ended up in three layers still in square conformation (on the edges of the nanotube, Figure S5D) and the other five layers created a helix with ~2.5 nm radius of the core. Part of the helix was disordered, but *ca.* two layers (eight **AU** molecules) were ordered, connected with hydrogen bonds (Figure S5D). These layers were separated from the structure and used for building a new, more ordered nanotube. In sharp contrast, **GC** stayed in individual layers/squares stacked on top of each other with ~2.8 nm radius of the core. The squares were slightly rotated on top of each other and created a positive twist (Figure S5D).

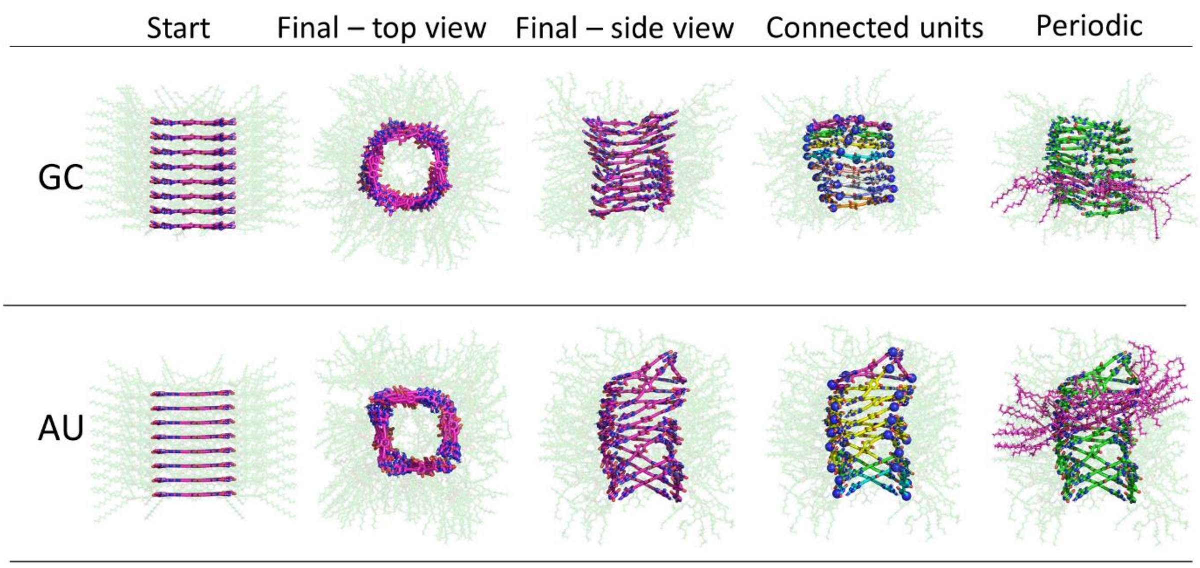

Figure S5D. Initial and final conformations of **GC** (top) and **AU** (bottom) starting with 8SQ separated by 0.5 nm. Connected units show the molecular cores with colours distinguishing between string of molecules – in **GC**, only squares are formed, in **AU** a long helix was observed (in yellow). The right panel shows the periodic unit used for further nanotube building.

Although both systems were allowed to evolve to an energetic minimum from the same stacked arrangement of cyclic tetramer entities, all the simulations performed clearly demonstrated a tendency of the **AU** molecules to develop spiral structures, and a strong resilience of the **GC** molecules to remain as intact cycles.

S5.2.3. Nanotube building

We used 8SQ structures as building block sources for building long and later periodic nanotubes. By visual inspection, we chose two layers from **GC** and **AU** structures, that seemed the most aligned over each other, oriented them in the *xy* plane, and searched for a proper spacing and rotation between individual layers. After identification of the most aligned structure (to the results of 8SQ systems), we built a tube with 20 layers (80 **AU**/**GC** molecules), simulated it for 50 ns, and compared its final and initial structures. We evaluated whether the rotation between individual layers is sufficient or if the system prefers a larger one. In several steps, we identified a setup for periodic nanotube and built it. The simulations were performed in the same way as in case of 2SQ and 8SQ (minimization, equilibration and production setup) and the production run was performed for 50 ns.

- **AU**. The **AU** nanotube was built in several ways. The successful way started with extracting one layer from 8SQ simulation (Figure S5D) and building a tube (20 layers) by keeping the structure similar to the final frame of 8SQ simulation (for details see Table S4, Step 1). Then ~50 ns MD simulation was performed. The final structure was oriented to keep the center-of-mass of the cores on the z-axis, in order to manipulate the building blocks just by z-shifting and z-rotation. By adjusting the z-spacing and rotation, another tube was built (Step 2) and again built in order to mimic the final structure of the 'Step 1' simulation. As the tube seemed to bend at the ends, a longer (40 layers) tube was built. After the simulation, the building block and

shifting/rotation was adjusted, and a ‘Step 3’ simulation was performed (Figure S5E). ‘Step 3’ simulation seemed to keep the initial conformation and was used further for building the periodic structure.

Table S4. Gradual adjustment of geometry of **AU** nanotube (Initial building block was taken from 8SQ simulation, then a building block from final frame of Step 1 was used for Step 2 building and final frame from Step 2 was used for building Step 3).

	Step 0	Step 1	Step 2	Step 3	Step 4
Rotation (°)	0	-2	-4.5	-5	-8
Z-spacing (nm)		0.34	0.40	0.42	0.42
X-spacing (nm)	0	-0.02	0	0	0
Y-spacing (nm)	0	0.05	0	0	0
#layer	8	20	40	periodic	periodic

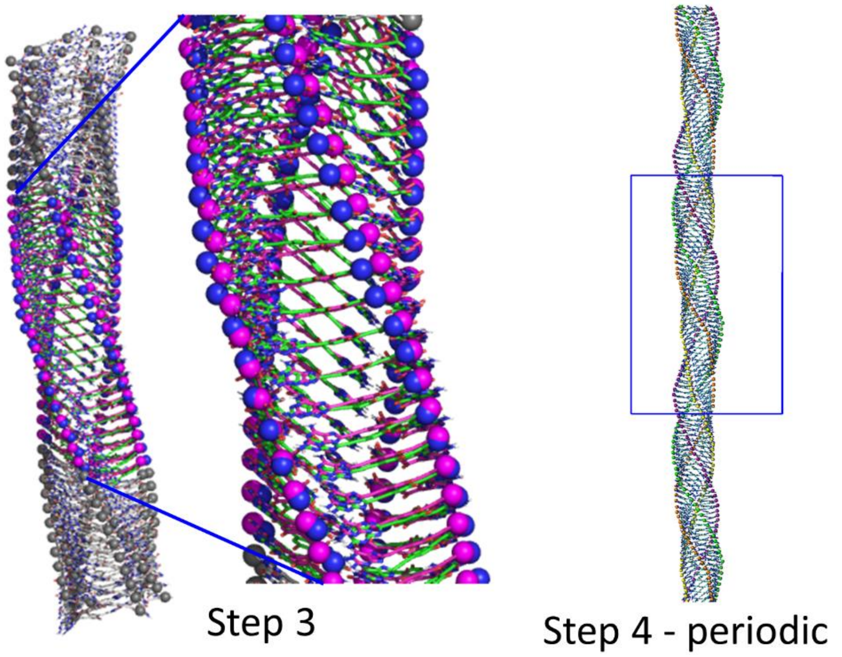

Figure S5E. Step 3 (left) simulation shown as a nanotube core comparing the initial (green, blue) and final (magenta) structure with the focus on the middle part. The side chains are omitted for clarity. The ends of the tube are depicted in grey. The detail of the middle part of the tube not affected by bending at the end is shown at the right side of the figure. The right panel shows the simulation box (blue) and the structure of the core of **AU** nanotube with its two periodic images (-8°, 0.42 nm in lower panel).

Periodic structure. Building a periodic structure was more complicated in **AU** than in **GC**. When the nanotube was built and simulated, the nanotube did not create a straight structure, but a bended one. The period of this bending is directly proportional to the length of the simulation box. However, by investigating a smaller part of the structure, very similar spacing/rotation is observed. Due to the simulations cost (smaller rotation needs more layers for the 360° and therefore larger simulation box with more atoms), further focus is on the setup with 0.42 nm z-spacing and -8° rotation (Figures S5E and S5F).

Hydrogen bonds. The adopted structure is based on H-bonds; therefore, we analysed their amount during the simulation (Figure S5J). For the analysis, 0.35 nm between donor and acceptor and 30° angle was taken as cut-off and we analysed them within **AU** molecules, between the **AU** cores (Watson–Crick

bonds) and within side chains. Per one layer (4 **AU** molecules) we observed 14.3 hydrogen bonds, of which 11.86 are within the cores and 2.29 are coming from the tails, mostly from the amide group between layers.

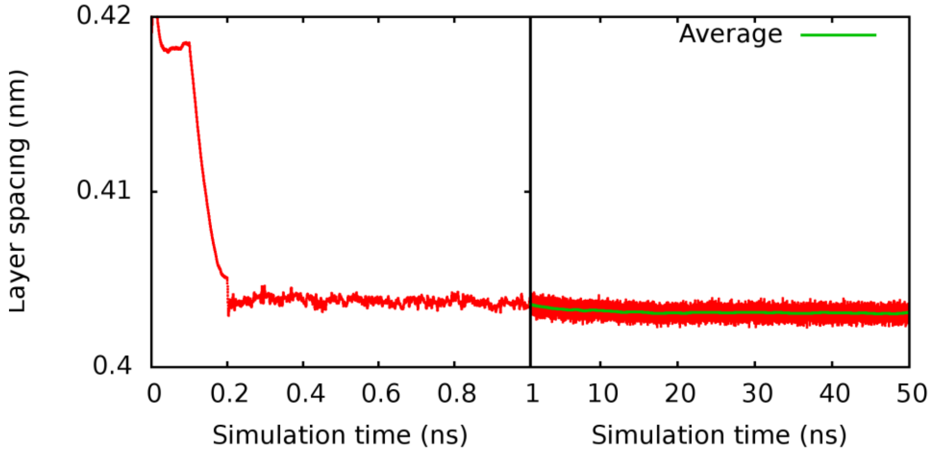

Figure S5F. Layer spacing in MD simulation merging all simulations (including equilibration steps). First 100 ps the position restraints were applied on the **AU** cores, during the other, unrestrained equilibration simulation (0.1 – 0.2 ns) the box size decreased, and the layers came closer to each other and further in MD simulation (for 0.2 ns) the box size did not decrease significantly (the average over each ns is shown in green).

- **<u>GC</u>**. In case of **GC**, the starting point for nanotube building was the simulation of 32 molecules in 8SQ on top of each other (Figure S5D). For this simulation, one square was chosen as a further building block. The square was chosen visually from a part with a higher ordering. The nanotube was built with followingly:
    - The initial building block was oriented in *xy* plane and centered to 0,0,0
    - With each layer, the initial building block was shifted in a *z*-direction by a '*z*-spacing'.
    - The initial building block was rotated around the around the *z*-axis.
    - The structure was visualized and compared to the final structure of previous MD simulation (in this case with simulation of 8 squares).

The *z*-shifting and rotation was performed by GROMACS tool editconf. It should be noted that in ordered regions of the nanotube the tails are also ordered enough to allow a nanotube building. The energy minimization is able to remove some artificial contacts between the tails, they just must not go through a ring (the simulation explodes) or be between the 'core' layers (the structure of the core is then disturbed).

First, we used *z*-spacing of 0.4 nm and rotation of 10° (Step 1). After 50 ns MD simulation, the nanotube tends to a larger rotation (Figure S5G) and smaller z-spacing, therefore we extracted another layer from this simulation and prepared a new tube with 0.38 nm z-spacing and rotated by 12° per layer (Step 2). Here the structure after 50 ns of MD simulation is almost identical to the initial one (Figure S5G). This setup was then used for building a periodic nanotube that seemed stable for 50 ns (Step 3), though the layer spacing decreased to 0.3577 nm (Figure S5G).

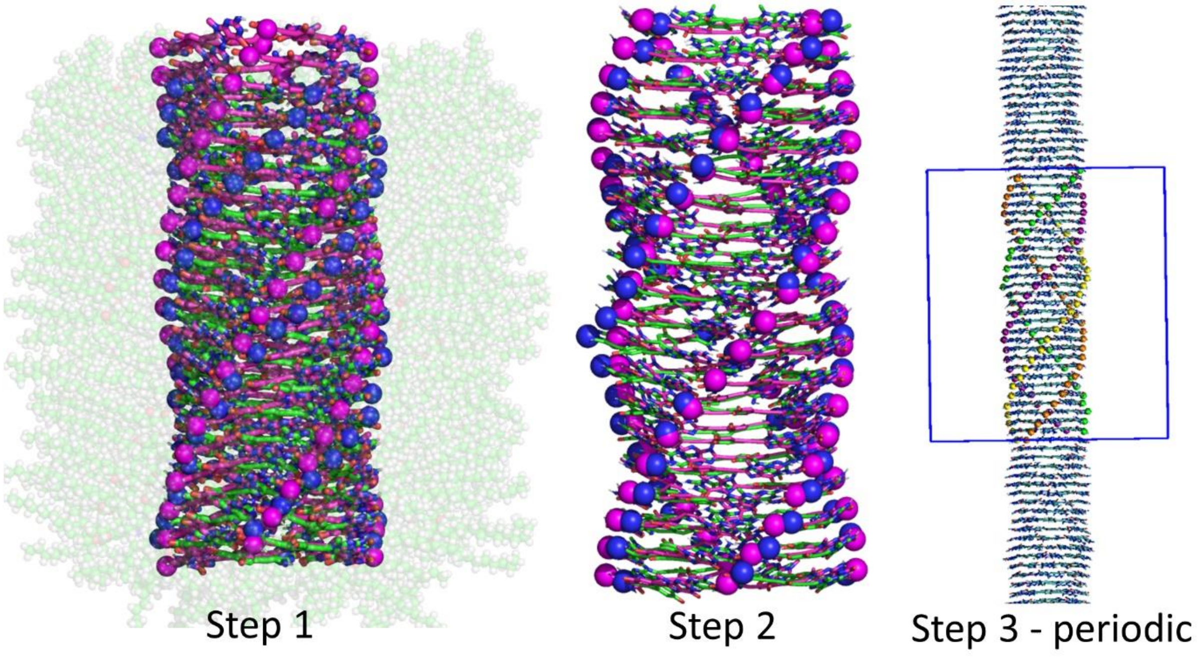

Figure S5G. Initial (magenta) and final (green+blue) structure of the nanotube in the first building step. During MD simulation, layers seemed to rotate more on each other than in 8SQ simulation. Middle: Initial (magenta) and final (green+blue) structures are almost identical (top left) and the same setup (0.38 nm between layers, 12° rotation of each layer) was used for building a periodic tube that is stable in MD as well (right).

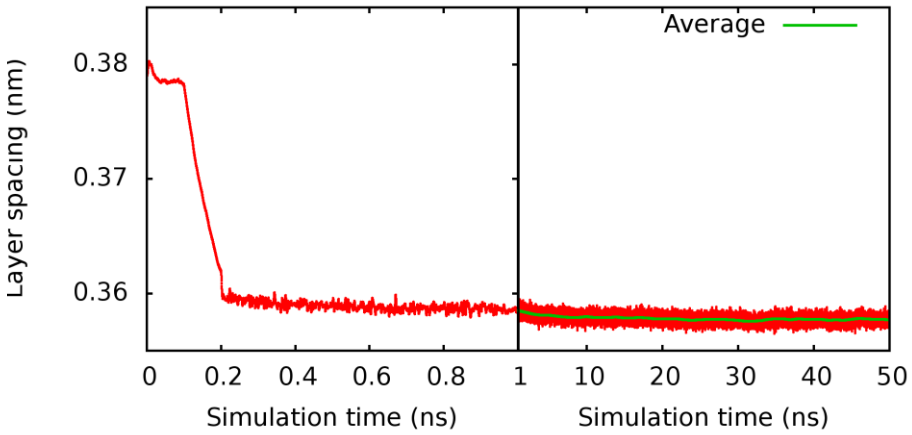

Figure S5H. Layer spacing in MD simulation merging all simulations (including equilibration steps). First 100 ps the position restraints were applied on the **GC** cores, during the other, unrestrained equilibration simulation (0.1 – 0.2 ns) the box size decreased, and the layers came closer to each other and further in MD simulation (for 0.2 ns) the box size did not decrease significantly (the average over each ns is shown in green).

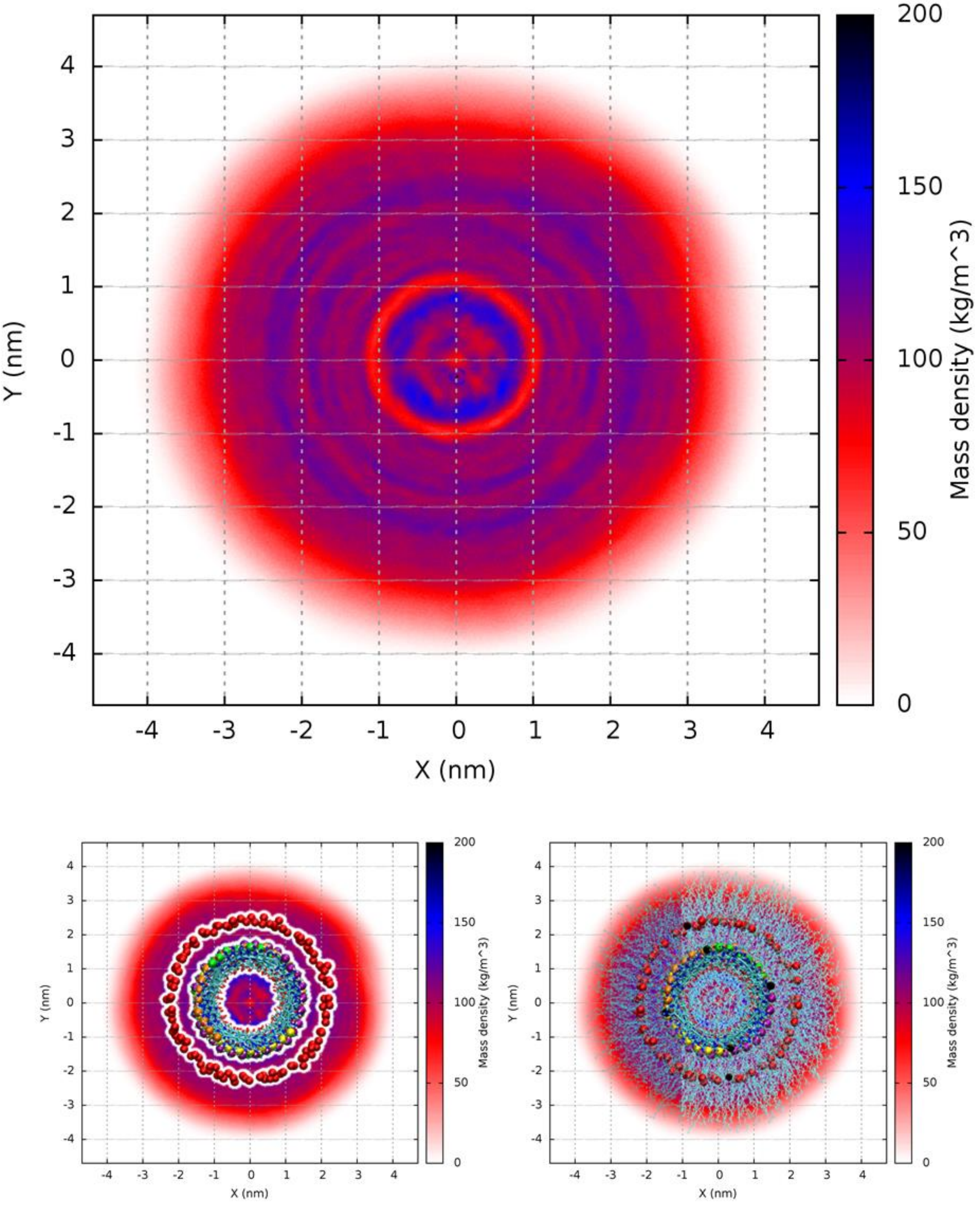

Figure S5I. Upper panel: Density map of a **GC** tube. Mass is averaged over the nanotube axis. Lower panel: density map and the **GC** tube structure. The **GC** core is shown in sticks, one of the phenyl ring oxygens is shown in red (left). Alkyl tails are shown in cyan lines (right).

H-bonding network. The **GC** square hold together with a H-bonding network. On average, there are 15.6 H-bonds coming (H-bonds donor involved in H-bond) from each layer (4 molecules, 3.9 per molecule). Of these, 11.7 H-bonds are connecting the molecular cores by the Watson–Crick bonding (2.9 H-bonds per molecule). Apart from that, there is 1.9 amide H-bond per layer in the side chains, increasing the stability of the tube, and another 2.0 bonds are established between the molecular cores and side chains (both inside the layer and connecting nearby layers). For the analysis, 0.35 nm between donor and acceptor and 30° angle was taken as cutoff.

- **Differences between GC and AU**

The MD simulations showed several differences in the internal organization of the nanotubes:

- The layer spacing in **GC** nanotube is smaller (0.358 nm) than in AU (0.403 nm).
- The total amount of H-bonds among the nanotube is higher in **GC** (15.6 per four molecules, see Table S5) than in **AU** (14.3). The difference is coming mostly from H-bonds between side chains oxygens and the exocyclic amine group of guanine in the core (Figure S5J).
- Differences in the internal structure were observed. While **GC** stayed mostly in a square-like shape (~90° between the residues), **AU** edges were not so regular (Figure S5L, panel a). Notice that in the case of **AU** the sum of the most populated angles is not 360° – **AU** does not form a square, but a helix.
- Further, we observed a much higher tendency to twisting for the **AU** molecule than for the **GC** molecule (Figure S5L, panel c; Figure S5B).

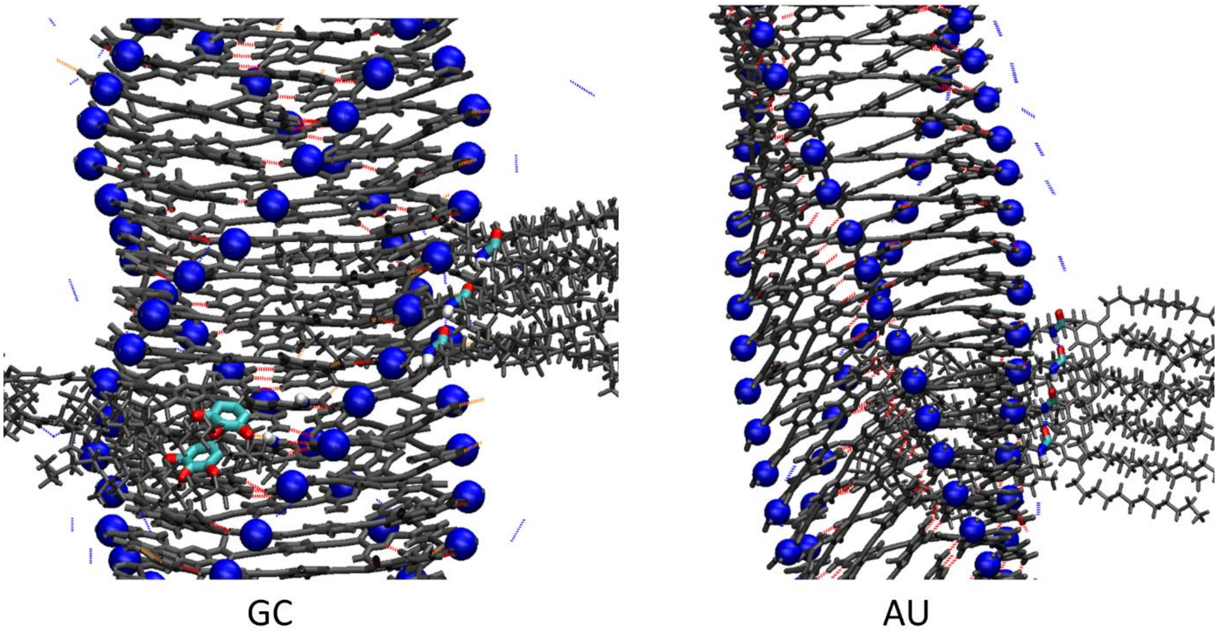

Figure S5J. H-bonds in **GC** and **AU** nanotubes – Watson–Crick pairs are depicted in red, amide H-bonds in blue (highlighted in right center part of the figure) and the H-bonds between side chains and cores are in orange (just in the case of **GC**, exocyclic amine group of guanine to one of the oxygens of the peripheral benzyl group).

Table S5. Number of H-bonds within **AU**/**GC** molecules (Total), Watson-Crick interactions within their cores (Core), Interlayer amide-amide H-bonds within side chains (Side) and the side chains – core H-bonds (Core-Side) and the interaction energies within cores, side chains and between them.

**H-bonds** (# per 4 molecules)	**AU**	**GC**
Total	14.34	15.58
Core	11.86	11.73
Side	2.29	1.86
Core-Side	0.19	1.99
**Interaction energies** (kJ/mol per 4 molecules)		
Core-Core	-6407	-7764
Core-Side	-1117	-885
Side-Side	-3874	-3384

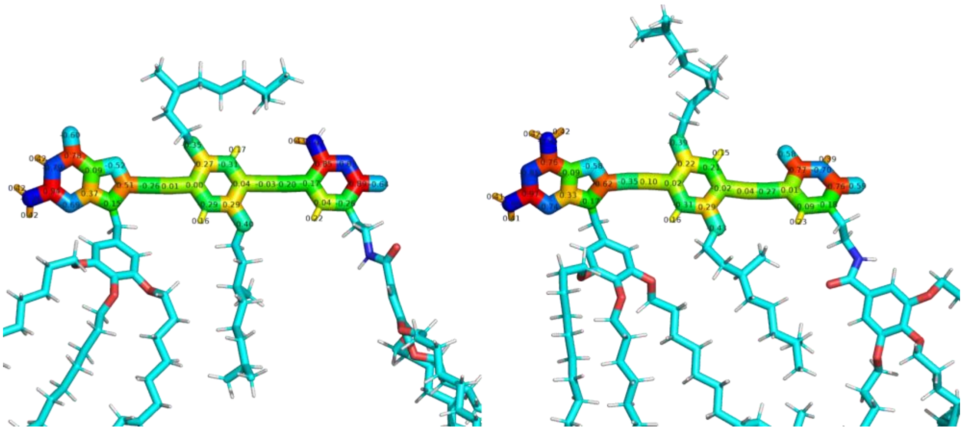

Figure S5K. Partial charges on the core in **GC** (left) and **AU** (right).

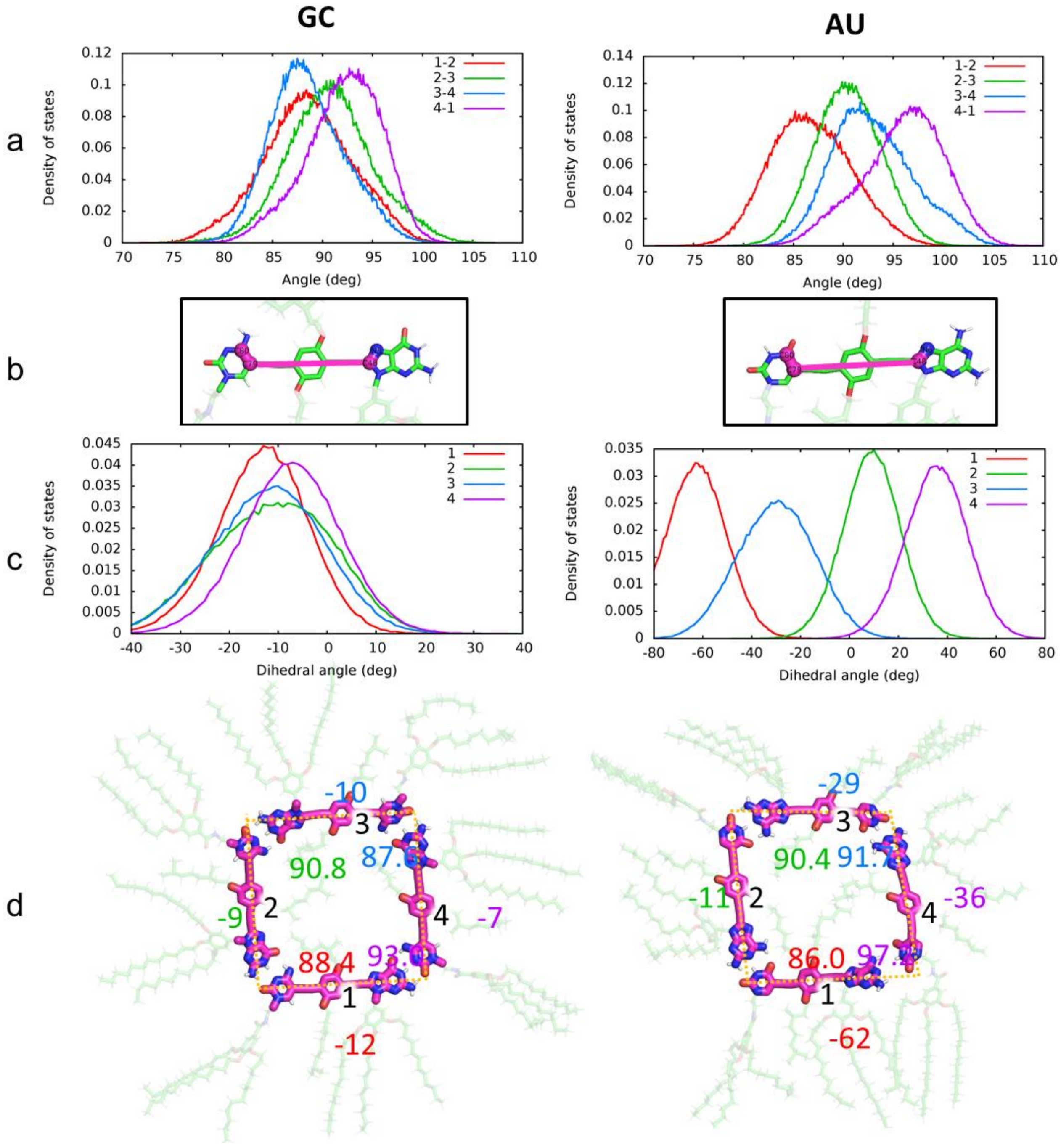

Figure S5L. (**a**) The distribution of the angles between individual molecules for **GC** and **AU** nanotubes. The angles are separated for different edges of the stacked tubes. (**b**) The structure of one **GC** and **AU** molecule, highlighting the dihedral angle of a monomeric unit monitored in (c) for the individual four edges of the nanotube. Used numbers for the edges correspond to structures in (**d**). (**d**) Structure of one **GC** and **AU** layer. The number inside correspond to the most populated angles between the individual residues (vectors shown by yellow dashed lines, corresponds to graph in (**a**)). Numbers outside correspond to the most populated dihedral angle in residues at that specific edge (from (**c**)).

5.3. Calculation of absorption and CD spectra

For calculation of CD spectra, we extracted conformations from MD simulations. We used VeloxChem[26] with exciton coupling module and calculated absorption and CD spectra with B3LYP/cc-pVDZ level of theory, taking into account five excited states per molecule.

We extracted ten layers of **GC** or **AU** from MD simulations (just the core was used, side chains substituted by methyls, no further geometry optimization was performed) and calculated absorption and CD spectra with B3LYP/cc-pVDZ level of theory, five excited states per molecule were considered, calculated by VeloxChem with exciton coupling model with 2eV broadening.

In order to save computational time, we tried to reduce the number of layers for the calculation. We compared results from 10 layers (40 molecules) to 4 layers (16 molecules) and observed a slight blue shift with increasing the size of the system (consistent with experimental results of tube growing, Figure S5M). Also in the case of **AU**, the excitonic band was clearer in the case of the larger system, but generally, the shape was preserved even for the reduced system.

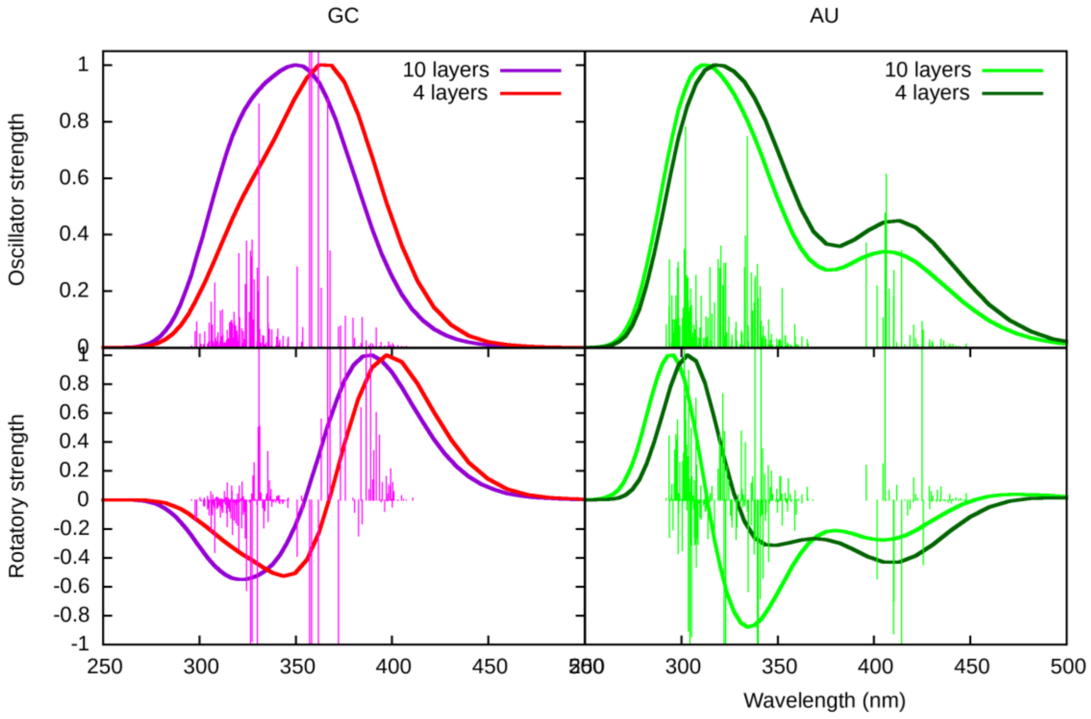

Figure S5M. Normalized absorption and CD spectra calculated on 10- and 4-layers systems with 0.2 eV broadening.

We calculated CD spectra of four and ten layers extracted from a final snapshot of the MD simulation. As the resulting spectra were very similar, we calculated ensemble average spectra by extracting ten frames over the simulation and from each we extracted four consecutive layers at random position. We calculated CD spectra with 0.2 eV broadening of calculated impulses and averaging the calculated ten spectra to the final average spectrum. While in case of **GC** (Figure S5N), all calculated spectra were very

[26] Rinkevicius, Z.; Li, X.; Vahtras, O.; Ahmadzadeh, K.; Brand, M.; Ringholm, M.; List, N. H.; Scheurer, M.; Scott, M.; Dreuw, A.; Norman, P. VeloxChem: A Python-driven density-functional theory program for spectroscopy simulations in high-performance computing environments *WIREs Computational Molecular Science* **2020**, *10*, e1457.

similar, in case of **AU** (Figure S5O), some of the individual spectra are not in agreement with experimental data and their ensemble average is necessary of a reliable prediction of CD spectrum.

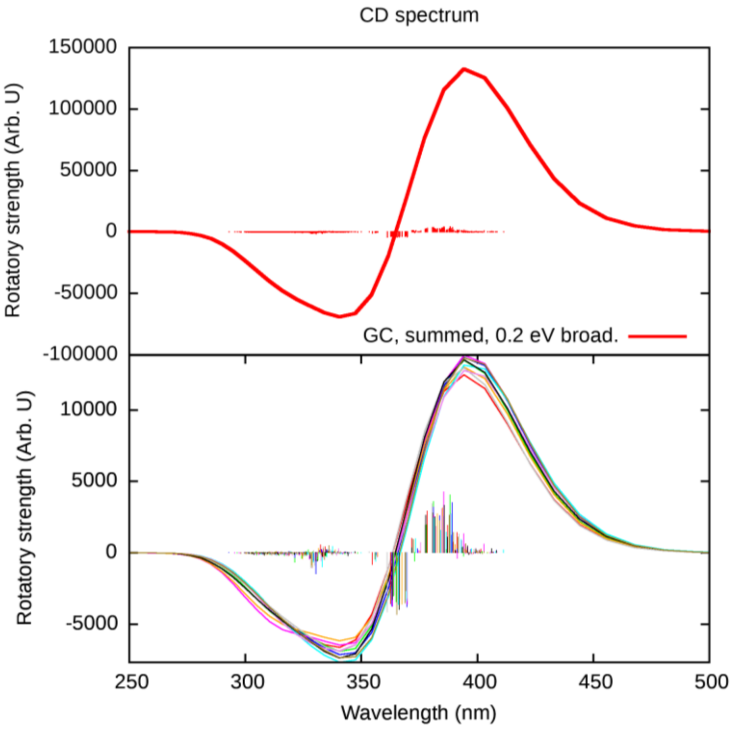

Figure S5N. Ten different snapshots were extracted for the MD simulations of **GC** nanotubes and from each snapshot a different set of four layers were extracted. Individual CD spectra are shown in the bottom panel, while a summed spectrum is shown in the upper panel.

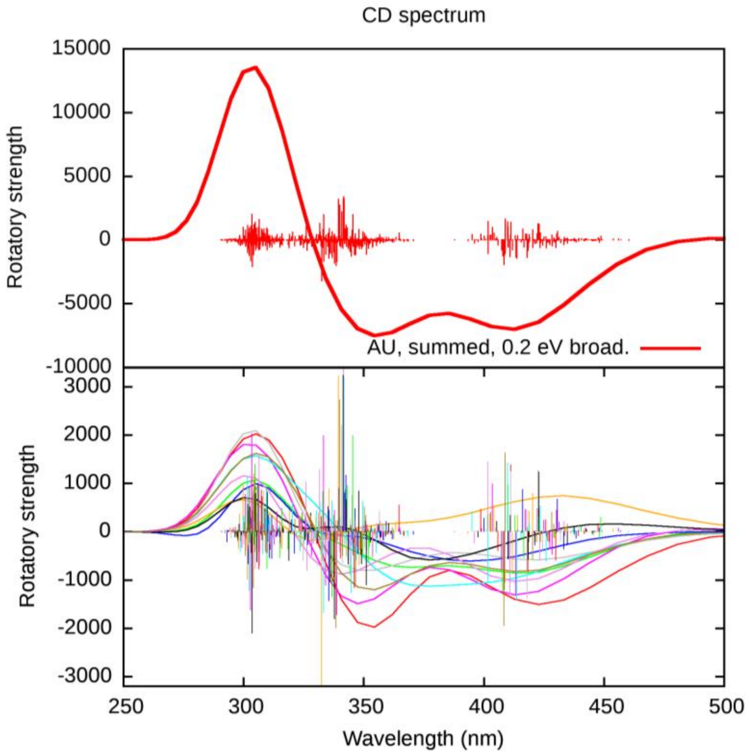

Figure S5O. Ten different snapshots were extracted for the MD simulations of **AU** nanotubes and from each snapshot a different set of four layers were extracted (16 consecutive **AU** molecules with a random starting number). Individual CD spectra are shown in the bottom panel, while a summed spectrum is shown in the upper panel.

Further, we wanted to analyse where do individual bands come from. Therefore, we prepared much more simplified models, taking into account just the linkers between nucleobases (Figure S5P) or even just the linker between rings (excluding phenyl ring in the middle). However, we cannot easily take the reduced fragments and compare their spectra with the original ones due to their blue shift with reducing size (Figure S5Q). This is consistent with a red shift observed in prolonging conjugated chains.

The shape of CD spectra is preserved showing positive or negative twist in all cases (Figure S5Q). But while the linker including phenyl ring had a simple clear CD spectrum, short linkers had a very small positive peak and very large negative one. This could be also a result of methylation, capping the ends of linkers with hydrogens, oriented in one direction.

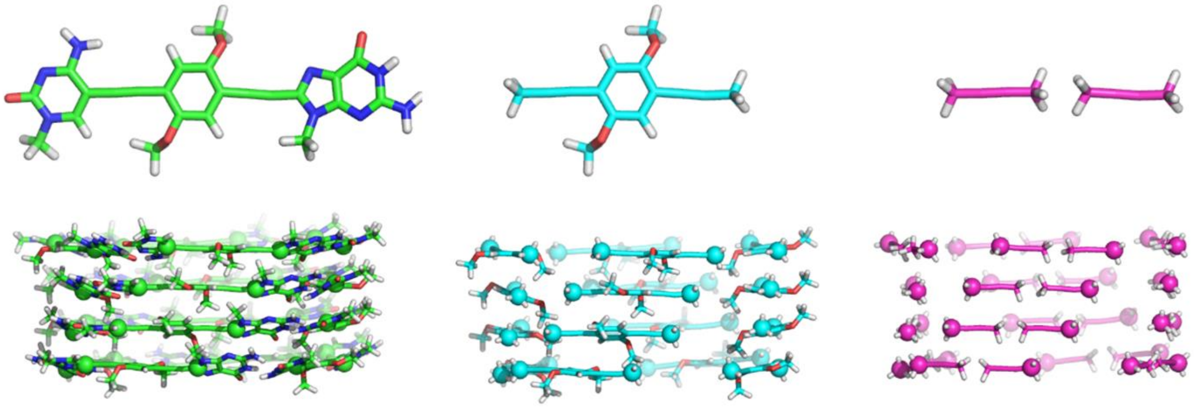

Figure S5P. Structures used for spectra calculation as used in Figure 4: Full **GC** (green, left), Linker + phenyl (cyan, middle) and Linker (magenta, right) shown as single molecules (upper panel) and in four layers system (lower panel).

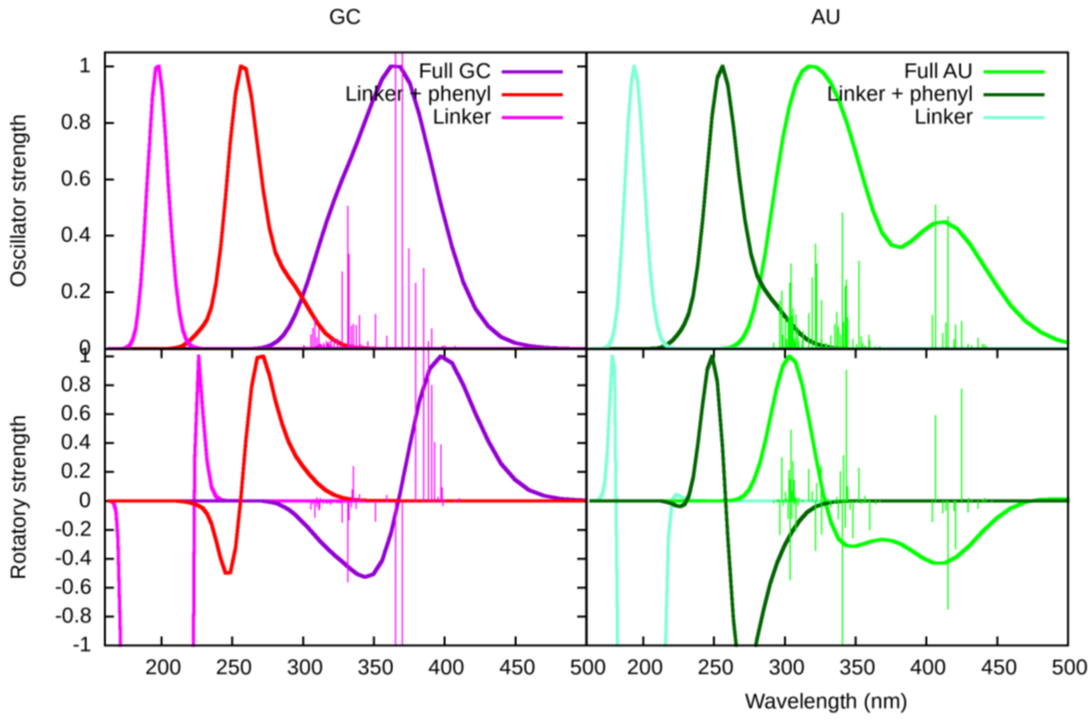

Figure S5Q. Normalized absorption (upper panel) and CD (lower panel) spectra of **GC** and **AU** calculated in 4 layers systems for the full molecule, linker between nucleobases including middle phenyl ring (linker + phenyl) and linker just between ring structures with 0.2 eV broadening.
